# Comprehensive Analysis of Chemical Ingredients of Waiganfengsha Granule and Absorbed Components in Rat Plasma Based on UHPLC-Q-TOF-MS

**DOI:** 10.2174/0113892002299899240515092703

**Published:** 2024-05-23

**Authors:** Wei Wei, Liyuan Huang, Jun Huang, Jinhua Li, Yingying Qing, Xiaotao Hou, Wen Liu

**Affiliations:** 1 Guangxi Key Laboratory of Efficacy Study on Chinese Materia Medica, Guangxi University of Chinese Medicine, Nanning, Guangxi, 530200, China;; 2 Faculty of Pharmacy, Guangxi University of Chinese Medicine, Nanning, Guangxi, 530200, China

**Keywords:** Waiganfengsha granule, UHPLC-Q-TOF-MS, component library, integrated strategy, chemical investigation, metabolites profile

## Abstract

**Objective:**

Waiganfengsha Granule, an over-the-counter drug, is commonly used for treating wind-heat cold and sore throat in clinical settings. However, its material basis of medicinal efficacy is still unclear. In this study, an efficient integrated analytical strategy was established for its chemical and metabolite profiles study.

**Methods:**

Firstly, to avoid the possible false-positive results of structural elucidation, an in-house component library that contains chemical constituents reported in the literature from the six individual medicines of Waiganfengsha Granule was established. Secondary, mass data post-processing techniques, including precursor ion list and neutral loss filtering, were applied to enhance the identification accuracy. Thirdly, for the rapid characterization of those absorbed components after oral administration in rats, the identified chemical constituents were used as candidate components for the serum analysis. By comparing the retention time and analyzing mass data, the metabolites in rat plasma were identified.

**Results:**

As a result, 57 chemical ingredients were identified, including 21 phenolic acids, 9 alkaloids, 2 flavonoids, 5 lignins, 13 saponins, and 7 other compounds. Among these, 12 compounds were unambiguously identified by comparison with reference standards, and 45 were tentatively characterized by analyzing their accurate MS data, MS/MS fragmentation patterns, and also by comparison with those data reported in the literature. Additionally, 46 metabolites were detected and identified in rat plasma.

**Conclusion:**

This study is beneficial for understanding the chemical composition and metabolic profiles of Waiganfengsha Granule, and the results obtained might provide a solid basis for further studies on its functional mechanism.

## 1 INTRODUCTION

Traditional Chinese medicine (TCM) prescription, the main dosage form of TCM, has long been used to diagnose, treat, and prevent human disease in China and other Asia countries for thousands of years. Its excellent efficacy and related low toxicity have been proved by long-term clinical applications [1, 2]. In the past decades, research on the pharmacodynamics of substance basis has been considered the key and core of safe, effective and quality control of TCM [3]. Generally, TCM prescription consists of two or more than two single herbal medicines in accordance with the theory of TCM [4]. Due to the structural complexity of the plant secondary metabolites in each individual herbal medicine, a complex component system is generated after the compound compatibility, which makes the comprehensive characterization of the chemical constituents of TCM greatly challenging [5]. It has been adequately demonstrated that after the oral administration of TCM prescription, the metabolites in blood might have better bioavailability and bioactivity [6]. From this perspective, the chemical investigation *in vivo* metabolism study of TCM prescription is of vital importance for the interpretation of its pharmacodynamics substance basis.

Chemical analysis serves as the foundation for elucidating the effects and action mechanisms of TCM prescriptions. Traditionally, the investigation of secondary metabolites of herbal medicines involves laborious processes of separation, purification, and structural elucidation. In recent years, with the development of analytical technology, significant advancements have been made in characterizing chemical components. Among these technologies, hyphenated techniques such as liquid chromatography-tandem mass spectrometry (LC-MS/MS) are considered the most powerful analytical tools due to their various advantages, including high speed, wide measurable mass range, and high ratio of resolution [7]. Following MS and MS/MS data acquisition, the challenging task lies in fragment assignment and structural elucidation [8]. To facilitate the post-processing of MS data, various techniques such as precursor ion list (PIL) [9], mass defect filtering (MDF) [10], neutral loss filtering (NLF) [11], diagnostic ion filtering (DIF) [12], database matching (DM) [13], high resolution extracted ion chromatogram (HREIC) [14], and integrated analytical strategies [5, 15], have been developed and implemented to enhance the efficiency of component identification.

Waiganfengsha Granule (WGFSG) is a unique product from the Pharmaceutical Factory Affiliated with the Guangxi University of Chinese Medicine, manufactured in compliance with Chinese national drug standards. The formula for WGFSG is based on a traditional prescription from the Zhuang ethnic group in Guangxi Province, China, containing six medicinal herbs: *Xanthium sibiricum* (Cangercao in Chinese, CEC), *Herba vernoniae patulae* (Xianxiahua, XXH), *Zanthoxylum nitidum* (Liangmianzhen, LMZ), *Radix et caulis ilicis asprellae* (Gangmei, GM), *Helicteres angustifolia* (Shanzhima, SZM), and *Streptocaulon griffithii* (Tengkusen, TKS). WGFSG, available as an over-the-counter (OTC) drug, is commonly used for treating wind-heat cold and sore throat in clinical settings. Modern pharmacological studies have demonstrated its significant anti-bacterial, anti-pyretic, analgesic, anti-inflammatory and immune-enhancing properties [16]. Some researchers have focused on the preparation process and quality control of WGFSG [17-19]. Our previous studies have established the HPLC fingerprint of WGFSG and evaluated its anti-inflammatory effect [20-23]. However, the comprehensive chemical investigation and *in vivo* absorption of components of WGFSG have not yet been fully elucidated.

In this study, an efficient research strategy was established for the rapid chemical investigation of WGFSG and the characterization of those metabolites in rat plasma after oral administration. Constituents of each individual medicinal herb in WGFSG from the reported literature were collected to construct the in-house component library. Subsequently, raw MS and MS/MS data were acquired in information-dependent acquisition (IDA) mode by the LC-MS instrument. All components of the in-house library were imported into the SCIEX OS software to generate the theoretical precursor ion list (PIL). Then, the primary screening of candidate constituents was performed automatically by applying set filtering parameters. Finally, the structural elucidation of the candidates was carried out through detailed analysis of the MS and MS/MS fragmentation pathways, such as NLs, along with comparisons of retention time and MS/MS behaviors of the reference standards and those data reported in the literature. Following the chemical investigation of WGFSG, the retention time, MS, and MS/MS data of the identified components were obtained and utilized in the study of metabolites *in vivo*. To minimize the matrix interferences from endogenous substances, HREIC function of SCIEX OS software was employed to rapidly screen and confirm those absorbed constituents. This study might provide a useful analytical strategy for elucidating the material basis of WGFSG.

## 2 EXPERIMENTAL

### 2.1 Materials and Reagents

WGFSG was provided by Guangxi University of Chinese Medicine Pharmaceutical Factory (Lot No. 20190401). Twelve reference standards (HPLC, purity ≥ 98%), including protocatechuic acid, neochlorogenic acid, chlorogenic acid, cryptochlorogenic acid, caffeic acid, syringic acid, 1, 3-dicaffeoylquinic acid, isochlorogenic acid A, isochlorogenic acid B, isochlorogenic acid C, hesperidin, and rosmarinic acid, were all purchased from Chengdu Maidesheng Technology Ltd (Sichuan, China). Acetonitrile and formic acid used in the elution solvent were all LC–MS grades acetonitrile, methanol, and formic acid were purchased from Merck (Darmstadt, Germany). A Milli-Q 89 water purification system (Millipore, Billerica, MA, USA) was applied for the purification of deionized water. Other reagents used were of analytical grade.

### 2.2 Animals Treatment

Male SD rats (6-8 weeks, weighing 180-220 g) for the *in vivo* metabolic study were purchased from Hunan Slake Jingda Experimental Animal Co., Ltd with certificate number SYXK (Xiang) 2019-0001. Prior to the experiment, the rats were acclimated to the environment in an animal room for one week before oral administration. The room conditions were the same as those reported in our previous paper [15]: temperature was set at 24–26 °C, and the relative humidity was at 40–60%, respectively. All experiment procedures were carried out in accordance with the Regulations of Experimental Animal Administration issued by the State Commission of Science and Technology of the People’s Republic of China. The experimental protocols involving animals were approved by the Animal Ethics Committee of Guangxi University of Chinese Medicine, and all procedures were conducted in compliance with the relevant regulations and guidelines.

### 2.3 Drug Administration and Plasma Samples Preparation

Prior to the experiment, twelve male SD rats were fasted for twelve hours with access only to water. They were then randomly separated into two groups: an experiment group (n=6) and a blank group (n=6). The experiment group was intragastrically administered with a water solution containing WGFSG at a dose of 0.45 g/kg/d twice a day for 3 days. The blank group received the same volume of distilled water. One hour after the final oral administration, 3 mL of whole blood was collected from the abdominal aorta in heparinized tubes and centrifuged at 5000 r/min for 10 min. The resulting supernatants were transferred into new tubes and serum from the same group was mixed to eliminate individual differences and stored at -20 °C before use. A certain amount of each reference standard was dissolved in methanol to obtain the corresponding standard solution, respectively. Before LC–MS analysis, they were mixed together and then filtered through Millipore filters to give the mixed standard solution for qualitative analysis.

### 2.4 UHPLC-Q-TOF-MS and MS/MS Conditions

The analysis of the chemical components of WGFSG and absorbed components in rat plasma was conducted on an ACQUITY UPLC BEH C18 column (2.1 mm × 100 mm, 1.7 μm, Waters Corporation, USA). Shimadzu Nexera Prominence liquid chromatogram system was applied for UHPLC analysis. The column temperature was maintained at 40 °C throughout the separation and the flow rate for all the samples was kept at 0.4 mL/min. High-resolution mass data was acquired in both negative and positive ion modes using an electrospray ionization (ESI) ion source on the AB SCIEX X500R quadrupole-time of flight (QTOF) coupled with high resolution mass spectrometry (HRMS) (Applied Biosystems SCIEX, US).

Acetonitrile (solvent B) and 0.1% formic acid aqueous solution (solvent A) were used in the mobile phase. The elution gradient for UHPLC was optimized as follows: 0–2 min, 5–10% B; 2–5 min, 10–16% B; 5–10 min, 16–45% B; 10–13 min, 45–60% B; and 13–17 min, 60-100% B. 3 μL of each sample was injected into the LC-MS system for analysis, and the MS and MS/MS data acquisition was performed under the information-dependent acquisition (IDA) technology mode. The analytical parameters were similar to our previous report [15]. The MS mass range for both negative and positive ion modes was *m/z* 100-1500 Da. As for the MS/MS acquisition, the corresponding mass range was *m/z* 100-1000 Da. The other instrument parameters for MS data acquisition were set as follows: ion source gas 1 (GS1): 55 psi, ion source gas 2 (GS2): 55 psi, curtain gas: 35 psi, temperature: 600 °C, and CAD gas: 7. For TOF MS: declustering potential (DP): ± 80 V, collision energy (CE): ± 35 V, and CE spread: 0 V. For TOF MS/MS: declustering potential (DP): 80 V, collision energy (CE):± 35 V, and CE spread: 15 V, and accumulation time: 0.05 s. After data acquisition, SCIEX OS software (Ver. 1.3.1, AB SCIEX Co.) was utilized for the data post-processing.

## 3 RESULT AND DISCUSSION

### 3.1 Establishment of the in House Component Library

After the acquisition of the MS and MS/MS data, the chemical structure elucidation is a tough and time-consuming task, not only because the massive and informative MS dataset obtained but also the possible false-positive results generated by those components that possessed the same chemical formula but different chemical structures. To enhance the accuracy of compound identification, an in-house components library was established. Relevant literature on compound purification and identification, chemical investigation using LC-MS methods, quality control studies, fingerprint studies, *etc*., was reviewed and selected as the component sources for the in-house component library. By searching online databases, for example, Web of Science, CNKI, Google Scholar, SciFinder database, and so on, information on each component, including compound name, chemical formulas, and chemical structures, was recorded in the in house component library and listed in an Excel table for further structural elucidation. As a result, a total of 562 compounds from the six individual herbal medicines were collected. Among these, 30 from XXH, 148 from LMZ, 90 from TKS, 38 from SZM, 185 from GM, and 71 from CEC. Detailed information on each compound, including name, formula, and chemical structure, was recorded in an Excel table and presented in Table (**S1**).

### 3.2 Data Acquirement and Post Data Processing Strategy

Upon acquisition of data, the processing of extensive and informative MS and MS/MS data poses a significant challenge. To efficiently identify the chemical components in WGFSG, those components contained in the in-house library were used for the the generation of precursor ion list (PIL). The Excel table containing the collected compounds from the 6 individual medicines of WGFSG was imported into the SCIEX OS software to create the theoretical PIL, and the primary candidate precursor ions screening was performed automatically by using the 'Analytics' section of the software. In negative ion mode, adducts including HCOO^+^, and H^−^ were selected for PIL, while in positive ion mode, adducts including H^+^, Na^+^, and K^+^ were chosen. A mass tolerance of ± 10 ppm was set, and elements for the chemical formula were chosen as C, H, O, N, and S. Following the primary screening, peaks without corresponding MS/MS data were excluded manually, and by this way the identification range of targeted components was narrowed. Subsequently, manual analysis of the MS and MS/MS data was conducted by detailed analysis of their first-order accurate mass data, MS/MS fragment behaviors including NLs and DIs, and also by comparison with reference standards and those data reported in the literature. The workflow of the integrated research strategy is shown in the Graphical Abstract.

As a result, a total of 57 components were unambiguously or tentatively characterized. Among these, 12 compounds, including protocatechuic acid, neochlorogenic acid, chlorogenic acid, cryptochlorogenic acid, caffeic acid, syringic acid, 1, 3-dicaffeoylquinic acid, isochlorogenic acid A, isochlorogenic acid B, isochlorogenic acid C, hesperidin, and rosmarinic acid were identified by comparison the MS data with those of reference standards. And the component types of the constituents in WGFSG could be mainly categorized into phenolic acids, saponins and their sulfur-containing derivatives, alkaloids, flavonoids, lignins, long chain fatty acids, and more. The base peak chromatograms (BPC) of WGFSG in both positive and negative ion modes are displayed in Fig. (**1**), and the chemical structures of the identified components are displayed in Fig. (**2**). Detailed MS and MS/MS information of the main components is displayed in Table **1** and pages 108-160 of the Supporting information.

The complexity of the total ion chromatogram (TIC) of the bio-sample was attributed to both background interferences from endogenous substances and the low concentrations of metabolites. The mass signals of the targeted compounds were often obscured by other non-targeted peaks, posing a challenge in characterizing *in vivo* metabolites. In this study, a more precise and controlled HREIC method was utilized for the rapid screening of metabolites in rat plasma following oral administration of WGFSG. After the aforementioned structural identification of WGFSG, detailed information on each identified constituent, including chemical formulas, retention times, MS, and MS/MS data, was obtained. These identified ones were suggested as the possible absorbed compounds in rat plasma. Subsequently, the presence of these constituents in rat plasma was confirmed using the HREIC method and manual confirmation. The chemical formulas of the 57 identified components in WGFSG were inputted into the 'Extract ion chromatogram' feature of the SCIEX OS software, and the corresponding 'Peak Width' was set at 0.02 ppm. This resulted in the extraction and characterization of 46 compounds. The BPC in positive and negative modes of rat plasma after WGFSG administration, as well as the HREICs of the identified compounds in rat plasma, are depicted in Figs. (**3** and **4**), respectively.

### 3.3 Identification of the Typical Components of WGFSG

#### 3.3.1 Phenolic Compounds

Phenolic compounds identified in the WGFSG could be categorized into monomer and polymer phenolic acids based on their structural characteristics. Normally, phenolic compounds contain OH and/or COOH groups in their structures, and negative ion mode is more suitable for detection. The characteristic MS/MS fragment pathway of phenolic compounds is the NL of small molecules such as CO_2_ (44 Da), H_2_O (18 Da), and others. Additionally, due to the presence of CH_3_ or OCH_3_ groups in the structure, NL values of 15 Da and 32 Da were also observed. In some cases, a combination of NLs of the aforementioned molecules was observed. Compound **26** was used as a representative example of the structural elucidation of such components. The precursor ion base peak of compound **26** was observed at *m/z* 515.1185 in negative ion mode, which helped us to calculate the chemical formula of it as C_28_H_34_O_15_. Seven main fragment ions could be detected in its MS/MS spectrum. The fragment ion at *m/z* 353.0875 was yielded by losing the caffeoyl substituent. After that, it further generated the next product ion at *m/z* 335.0783 by NL of one H_2_O (18 Da). Other product ions such as *m/z* 191.05660 were deduced by losing two caffeoyl substituent groups from mother nuclear. Through comparison of its MS/MS fragment pathways and retention time with those of the reference standard, compound **26** was unambiguously identified as isochlorogenic acid B. The proposed fragment pathway of compound **26** is illustrated in Fig. (**5**).

#### 3.3.2 Flavonoids

Flavonoids are commonly found in many herbal medicines with high content, and their structures are defined by a diphenylpropane (C6-C3-C6) skeleton [24]. The typical NLs of flavone aglycones were 15 Da (CH_3_), 18 Da (H_2_O), 28 Da (CO), 30 Da (CH_2_O), and 44 Da (CO_2_), according to their different substituents. The most characteristic MS/MS fragments of flavonoids were those generated by the Retro-Diels-Alder (RDA) reaction occurring in the C ring, including *m/z* 137, 135, and 119, respectively. For the flavonoid glycosides, glycosyl side chains connected to the hydroxyl group at C-3 or C-7 position are prone to be expelled from the parent nucleus, and thus simultaneous or successive losses of 162 Da (Glc), 146 Da (Rha), and 132 Da (Xyl) were observed in their MS/MS spectrum [25]. Compound **28** was taken as a representative example of the structural elucidation of such components. Compound **28** displayed a deprotonated precursor ion ([M−H]^–^) at 609.1828. The subsequential losses of one Rha and one Glc residue from the side chain generated the main fragment ions at *m/z* 301.0716. By comparison with the MS and MS/MS behaviors with those of the reference standard, compound **28** was identified as hesperidin.

#### 3.3.3 Saponins and their Sulfur-containing Derivatives

Saponins are a structurally and biologically diverse class of glycosides of steroids and triterpenes. Due to the existence of polyhydroxy substitutions in the structure, saponins usually show intense deprotonated ion [M-H]^-^ or solvent adduct ion [M+HCOO]^-^ in the first-order mass spectrum under negative ion mode. The cleavage of sugar moiety was utilized to characterize saponins in WGFSG. The corresponding NLs for the dissociation of the sugar group were 162 Da (Glc), 146 Da (Rha), 132 Da (Xyl), and 176 Da (GluA), respectively. Other NLs, such as 18 Da (H_2_O), 36 Da (2H_2_O), 44 Da (CO_2_) and 62 Da (CO_2_+H_2_O) were also frequently observed in their MS/MS spectrum. For the sulfur-containing derivatives of saponins, similar fragmentation pathways were detected in their MS/MS spectra. Characteristic NL of 80 (SO_3_) was obvious according to the corresponding high intensive production ion yielded. Compound **44** was selected as a representative for structural elucidation. The MS/MS fragmentation pathway of compound **44** is illustrated in Fig. (**6**). Compound **44** gave the precursor ion of [M-H]^-^ at *m/z* 889.3897, indicating the molecular formula to be C_42_H_66_O_18_S. In the MS/MS spectrum, after the loss of one molecule of SO_3_ (80 Da), the fragment ion peak at *m/z* 809.4339 was observed. Due to the existence of the glycosyl group in the structure, product ions at *m/z* 727.3385 and 647.3825 were yielded from the precursor ion at *m/z* 889.3897 and [M-H-SO_3_]^-^ ion at *m/z* 809.4339, respectively. Fragment ion at *m/z* 254.9816 was formed by the substituted group located at C-3 position. Based on the above-mentioned evidence, compound **44** was tentatively identified as 3-methoxy-4,5-dihydroxybenzoic acid, a saponin from GM. The analysis of the fragmentation pathway of compound **44** suggested that the NL of 80 Da could be utilized for characterizing of the sulfur-containing saponin derivatives. The characteristic NL of SO_3_ (80 Da) in negative ion mode for four representative saponins (**44**, **46**, **52**, and **54**) with HSO_3_ in their structures is depicted in Fig. (**7**).

## CONCLUSION

It is well accepted that the chemical constituents of TCM are responsible for its excellent therapeutic effects [26]. In this study, a reliable and efficient research strategy adopting UHPLC-Q-TOF-MS technology combined with in-house component library and HREIC was established for the chemical investigation of WGFSG and absorbed compounds in plasma *in vivo*. As a result, a total of 57 ingredients were identified, including 21 phenolic acids, 9 alkaloids, 2 flavonoids, 5 lignins, 13 saponins, and 7 other compounds. Among these, 46 metabolites were detected in the rat plasma by HREIC. This study could provide the fundamental data on the chemical components of WGFSG for the first time and expand the understanding of its therapeutic material basis.

## Figures and Tables

**Fig. (1):**
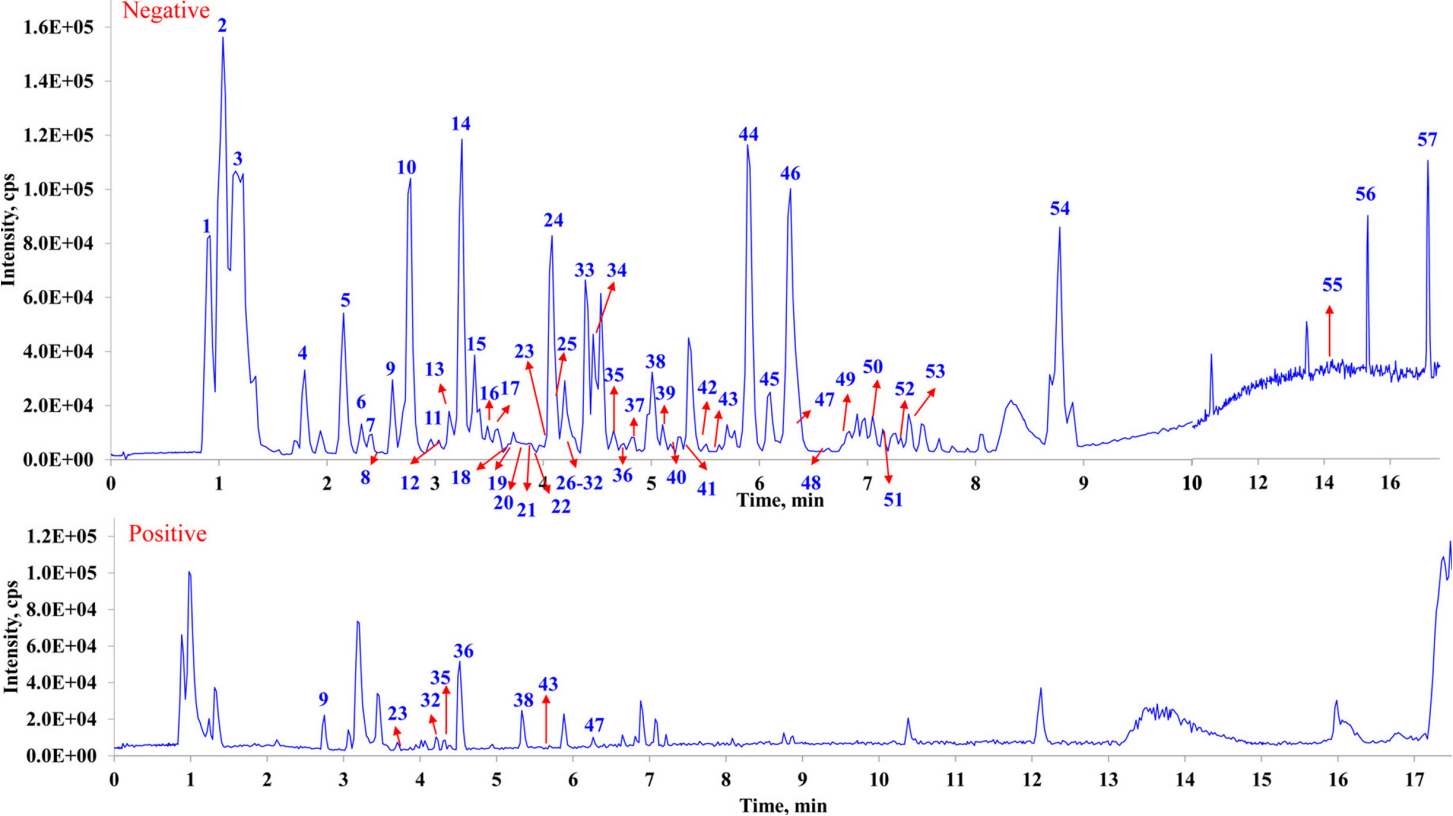
The BPCs of WGFSG in both negative (above) and positive (below) ESI ion modes.

**Fig. (2):**
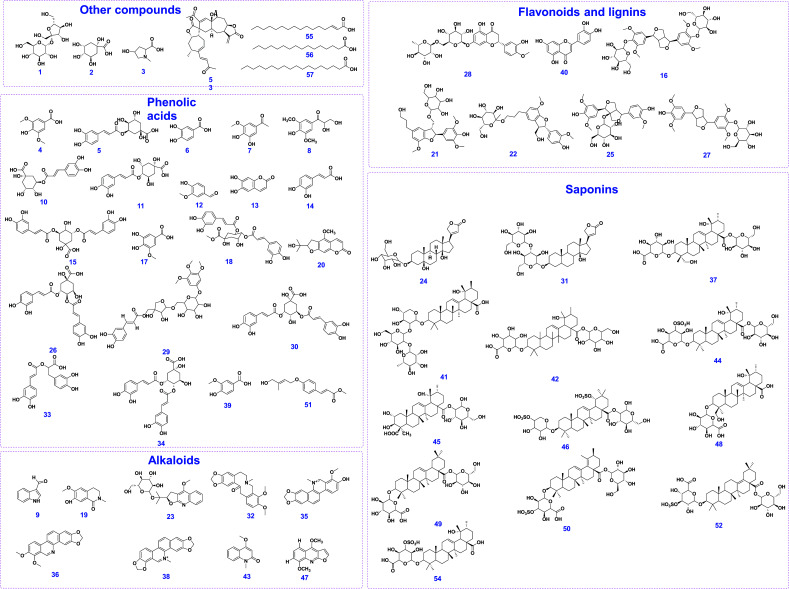
Chemical structures of the identified compounds in WGFSG.

**Fig. (3):**
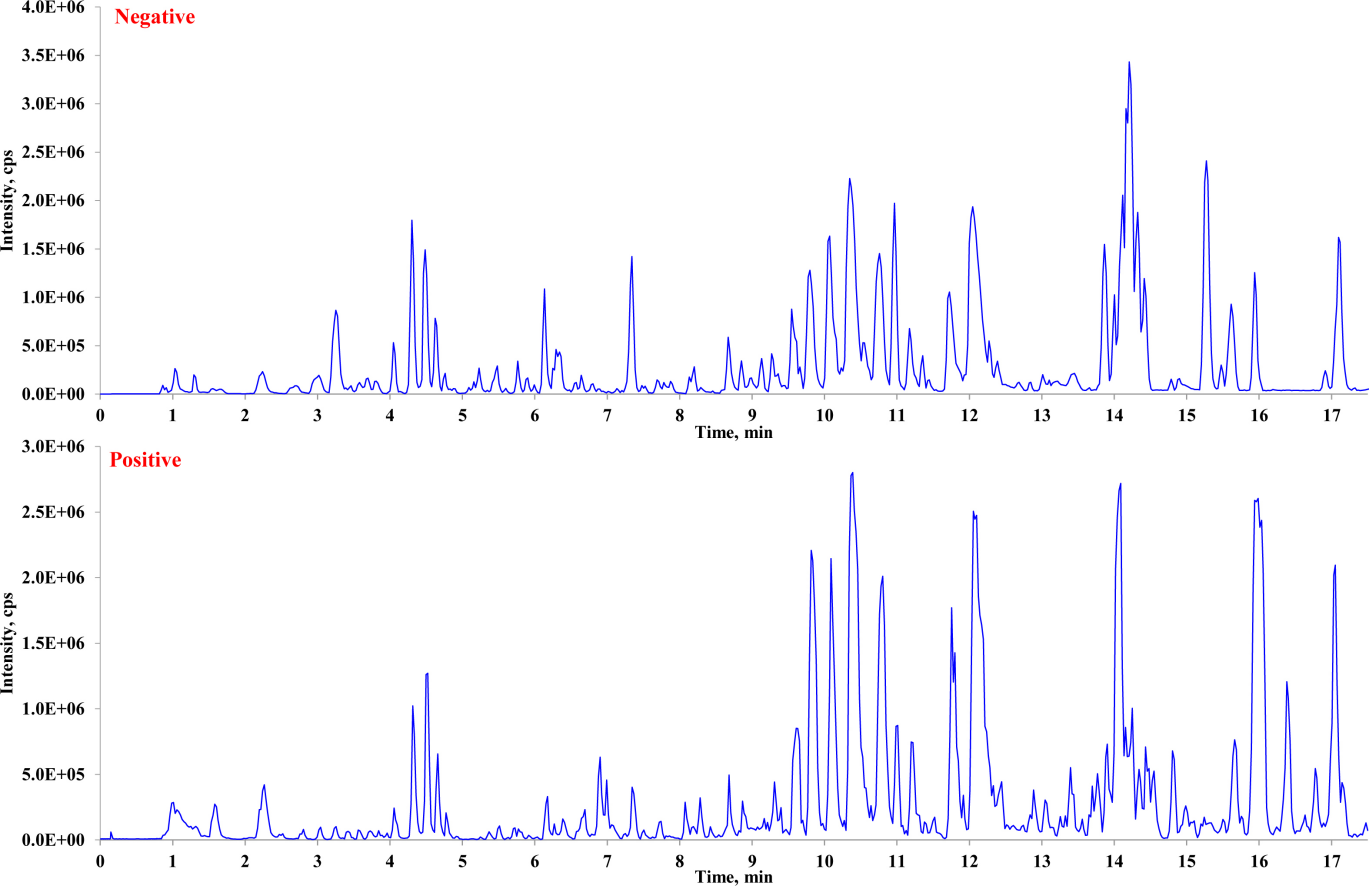
The BPCs of drugged rat plasma in the negative (above) and positive (below) ions modes.

**Fig. (4):**
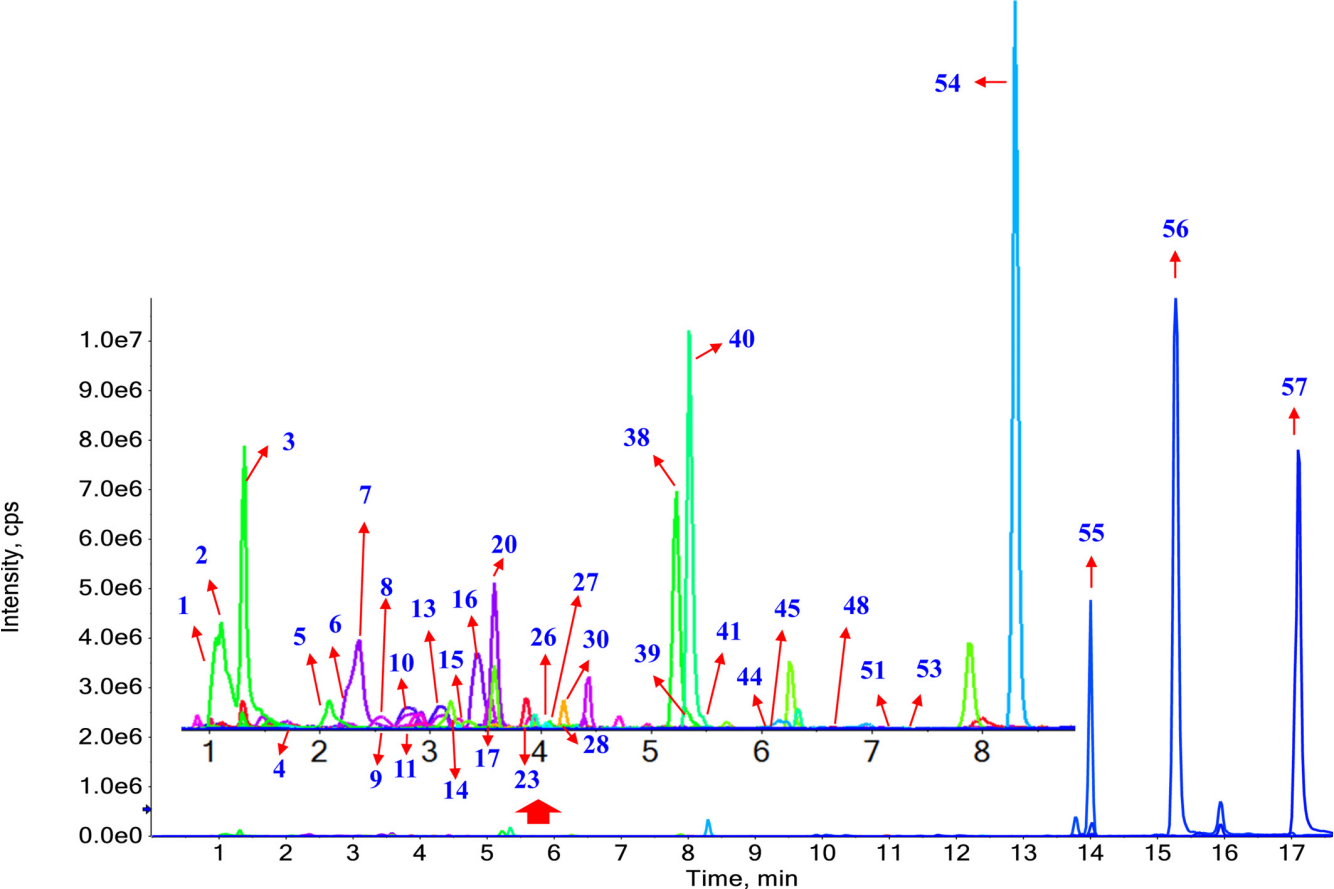
The HREIC of absorbed prototypes in rat plasma in negative ion mode.

**Fig. (5):**
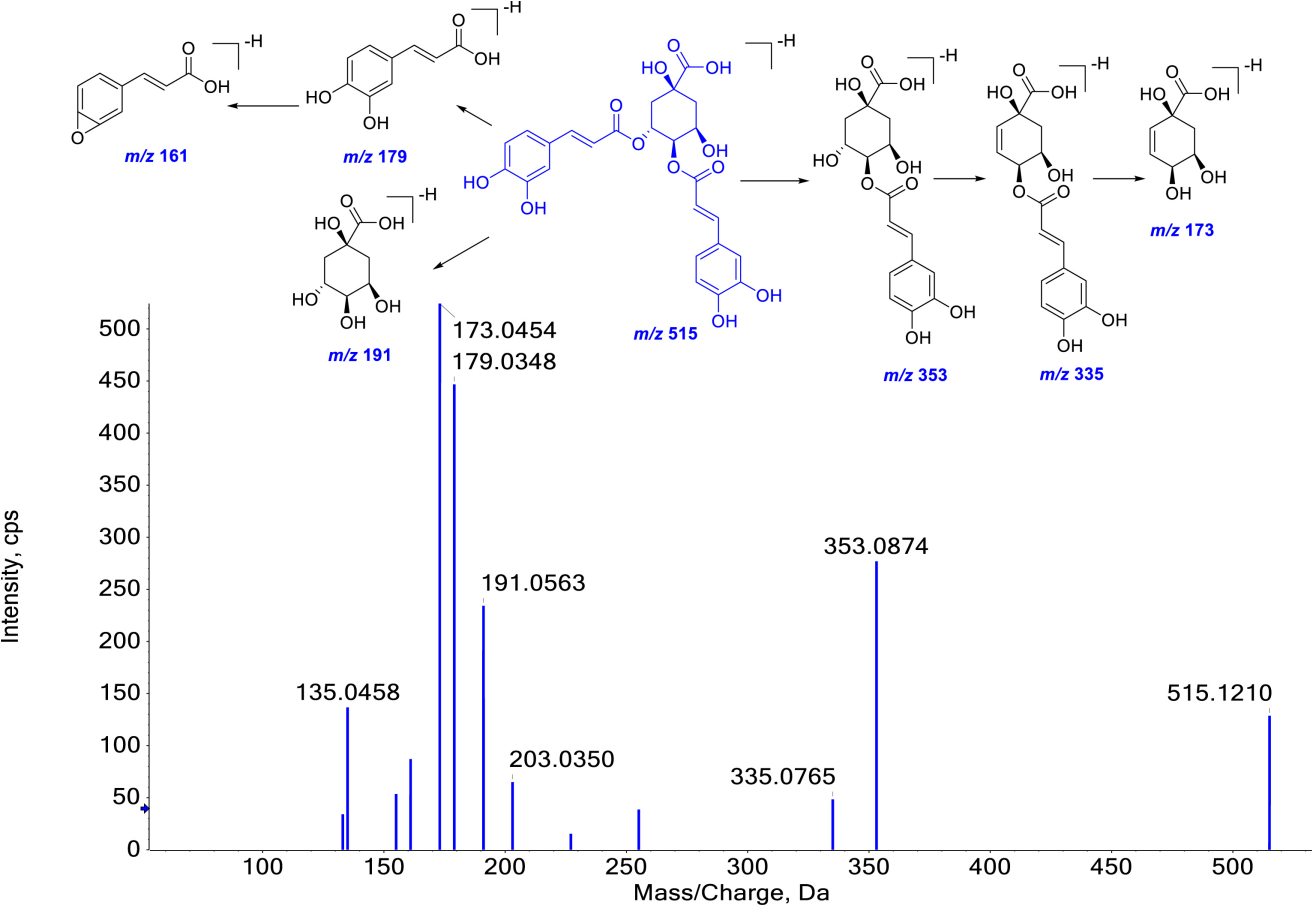
The possible MS/MS fragment pathway of the representative phenolic compound (26^*^) in negative ion mode.

**Fig. (6):**
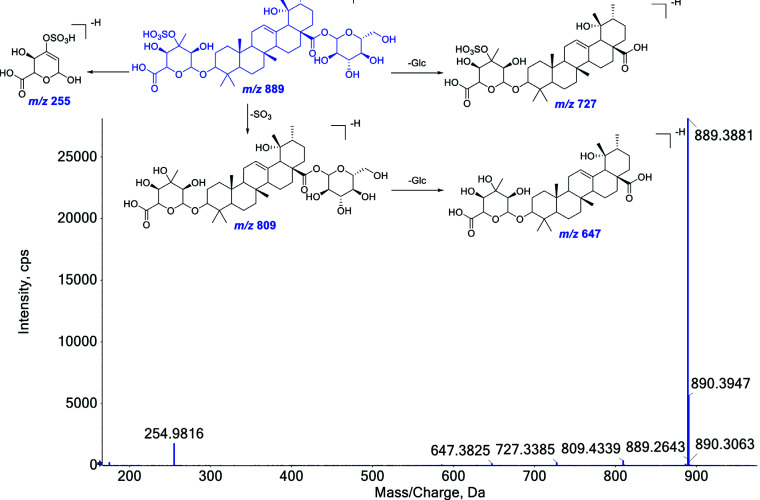
The possible MS/MS fragment pathway of the representative saponin compound (**44**) in negative ion mode.

**Fig. (7):**
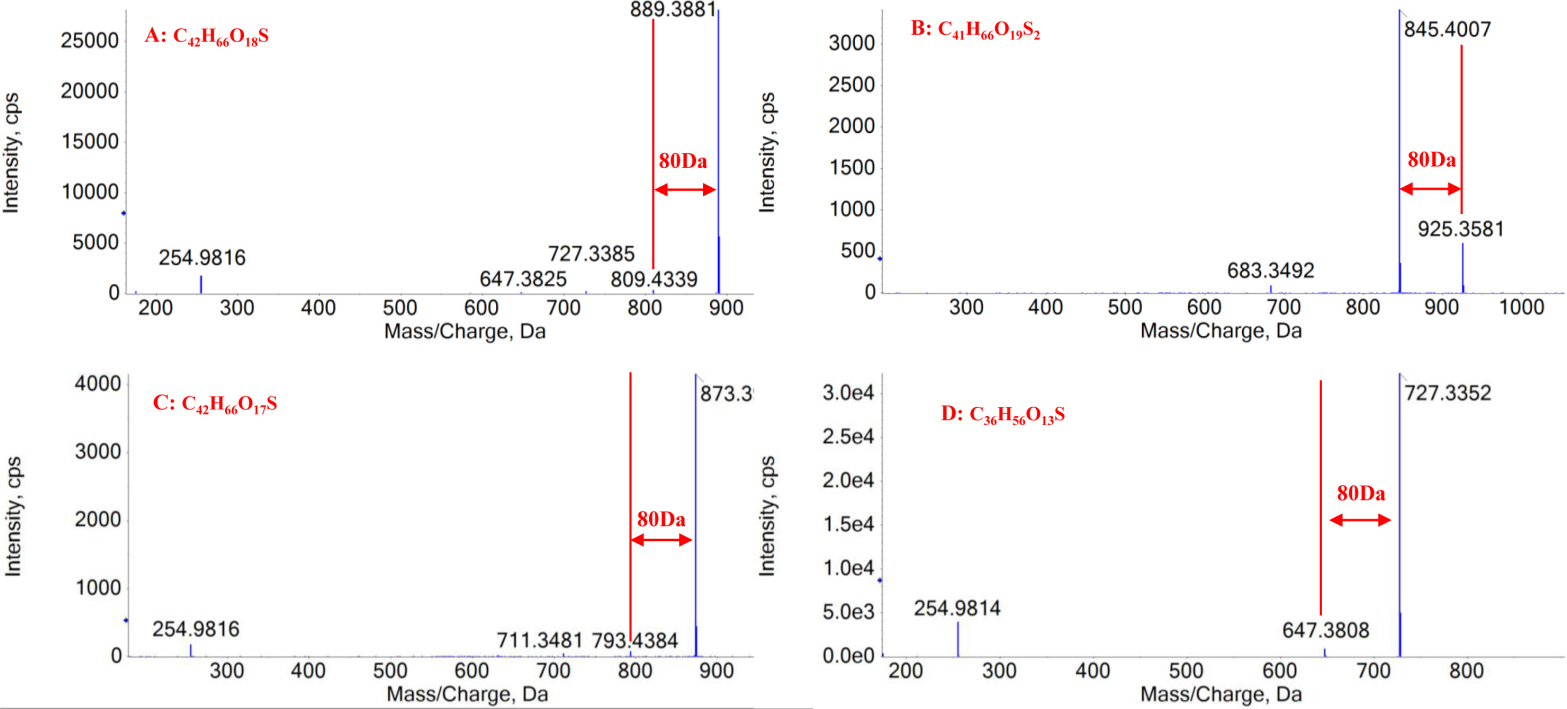
The characteristic NL of SO3 (80 Da) of the four representative saponins with HSO3 in the structures in negative ion mode. A: **44**, B: **46**, C: **52**, D: **54**.

**Table 1:** The detail information of the identified components in WGFSG by UHPLC-Q-TOF-MS and the absorbed ones in rat plasma (P).

**NO.**	**Retention Time (min)**	**Adduct / Charge**	**Precursor Mass**	**Mass Error (ppm)**	**Formula**	**Name**	**MS/MS data**	**Source**	**Absorbed prototypes**
**1**	0.99	[M-H]-	341.1089	0.9	C_12_H_22_O_11_	Sucrose	341.1103, 256.8490, 221.0687, 179.0561, 161.0458, 119.0350	CEC	P
**2**	1.04	[M-H]-	191.0561	0.5	C_7_H_12_O_6_	Quinic acid	191.0559, 171.0304, 155.0345, 137.0241, 127.0402, 109.0297	SZM	P
**3**	1.27	[M-H]-	144.0666	1.0	C_6_H_11_NO_3_	4-Hydroxy-N-methylproline	144.0668, 102.0557, 100.0743	LMZ	P
4*	1.79	[M-H]-	197.0455	-0.7	C_9_H_10_O_5_	Syringic acid	179.0352, 151.0339, 135.0450, 123.0451, 122.0373	LMZ TKS GM	P
5*	2.13	[M-H]-	353.0871	2.3	C_16_H_18_O_9_	Neochlorogenic acid	353.0885, 191.0556, 179.0345, 161.0246, 135.0447	XXH SZM CEC	P
6*	2.19	[M-H]-	153.0193	1.3	C_7_H_6_O_4_	Protocatechuic acid	153.0205, 135.0453, 109.0295	GM CEC	P
**7**	2.39	[M-H]-	181.0506	1.3	C_9_H_10_O_4_	4, 5-Dihydroxy-3-methoxyacet-ophenone	181.0495, 163.0411, 135.0458, 119.0515	GM	P
**8**	2.53	[M-H]-	241.0718	2.0	C_11_H_14_O_6_	7-O-ethylguaiacylglycerol	241.0745, 222.8601, 194.9065, 150.9155, 132.9046, 122.0381, 107.0142	GM	P
**9**	2.54	[M+H]+	146.06	1.2	C_9_H_7_NO	Indole-3-carboxaldehyde	146.0610, 128.0480, 117.0559	LMZ	P
10*	2.76	[M-H]-	353.0868	-1.0	C_16_H_18_O_9_	Chlorogenic acid	191.0547, 173.0456, 161.0245, 135.0451	GM	P
11*	2.77	[M-H]-	353.0873	-1.4	C_16_H_18_O_9_	Cryptochlorogenic acid	353.0879, 191.0555, 173.0447, 135.0446	SZM	P
**12**	3.08	[M-H]-	151.0401	1.6	C_8_H_8_O_3_	Vanillin	151.0394, 136.01787, 124.0164, 109.0297, 108.0216	TKS CEC	/
**13**	3.09	[M-H]-	177.0193	0.6	C_9_H_6_O_4_	Aeculetin	177.0195, 149.0241, 133.0301, 105.0351	GM	P
14*	3.14	[M-H]-	179.035	-0.2	C_9_H_8_O_4_	Caffeic acid	179.0351, 135.0448, 117.0346, 107.0497	XXH GM CEC	P
15*	3.24	[M-H]-	515.1195	-1.2	C_25_H_24_O_12_	1, 5-Di-O-caffeoylquinic acid	515.1197, 353.0873, 335.0779, 191.0554, 179.0345, 135.0448	CEC	P
**16**	3.4	[M+FA-H]-	787.2666	1.3	C_34_H_46_O_18_	(+)-Syringaresinol-4'-O-β-D-monoglucoside	787.1018, 579.2080, 417.1570	GM	P
**17**	3.61	[M-H]-	183.0299	0.2	C_8_H_8_O_5_	Syringicacid-4-O-α-L-rhamnopyranoside	183.0703, 139.0762	GM	P
**18**	3.67	[M-H]-	529.1351	1.2	C_26_H_26_O_12_	3, 4-di-O-caffeoyl quinic acid methyl ester or 4, 5-di-O-caffeoyl quinic acid	529.2671, 481.2477, 353.0891, 191.0563, 179.0352, 135.0454, 134.0375	XXH GM	/
**19**	3.69	[M-H]-	206.0823	1.5	C_11_H_13_NO_3_	Thalifoline	164.0873, 164.0731, 147.0463, 103.0563	LMZ	/
**20**	3.69	[M-H]-	291.0874	-5.2	C_15_H_16_O_6_	5-Methoxymarmesin	291.0850, 247.0942, 226.8779, 208.8666, 189.0557, 208.8666, 173.0213, 153.0163, 123.0820	LMZ	P
**21**	3.7	[M-H]-	551.2134	1.4	C_27_H_36_O_12_	Salicifoneoliganol	551.2166, 389.1613, 343.1165, 341.1054, 203.0724, 179.0344, 135.0447	GM	/
**22**	3.73	[M-H]-	521.2028	1.0	C_26_H_34_O_11_	(7S,8R)-dihydrodehydrodiconiferylalcohol-9'-β-D-glucopyranoside	521.2059, 359.1521, 323.0765, 197.0461, 179.0353, 161.0245, 135.0456	GM	/
**23**	3.96	[M+H]+	422.1809	1.5	C_21_H_27_NO_8_	Zanthonitiside A	422.1833, 260.1282, 242.1183, 188.0719	LMZ	P
**24**	4.03	[M+FA-H]-	597.2917	-1.5	C_29_H_44_O_10_	Periplogenin glucoside	597.1475, 551.2147, 417.1379, 389.1614	TKS18	/
**25**	4.11	[M+FA-H]-	611.1981	1.4	C_27_H_34_O_13_	(+)-Fraxinresinol-1-O-β-D-glucopyranoside	611.1985, 565.1901, 385.1309, 325.1072	GM	/
26*	4.12	[M-H]-	515.1185	2.1	C_25_H_24_O_12_	Isochlorogenic acid B	515.1192, 353.0875, 335.0783, 191.0560, 179.0343, 173.0450, 135.0449	SZM CEC	P
**27**	4.16	[M-H]-	579.2083	1.7	C_28_H_36_O_13_	Clove fat o-β-D-glucoside or (+)-cycloolivil	579.2109, 511.2540, 417.1558, 402.1319, 387.1088, 181.0510, 166.0270, 137.0252	GM	P
28*	4.24	[M-H]-	609.1825	0.5	C_28_H_34_O_15_	Hesperidin	609.1828, 301.0716	LMZ TK	P
**29**	4.26	[M-H]-	639.1931	1.4	C_29_H_36_O_16_	3,4,5-Trimethoxyphenol-β-D-5-O-caffeoyl-apiofuranosyl-(16)-β-D-glucopyranoside	639.1949, 455.1209, 395.0984, 353.0887, 179.0352, 135.0451	GM	/
30*	4.29	[M-H]-	515.1185	3.5	C_25_H_24_O_12_	Isochlorogenic acid A	515.1210, 353.0868, 335.0789, 191.0551, 179.0340, 135.0445	SZM CEC	P
**31**	4.31	[M+FA-H]-	743.3496	2.3	C_35_H_54_O_14_	Digitoxigenin sophoroside Digitoxigenin gentiobioside	743.3633, 697.3461, 563.1172, 535.2914, 383.0757	TKS	/
**32**	4.32	[M+H]+	370.1649	-0.6	C_21_H_23_NO_5_	α-allocryptopine	370.1651, 352.1542, 290.0942, 206.0819, 188.0714	LMZ	/
33*	4.4	[M-H]-	359.0772	-0.7	C_18_H_16_O_8_	Rosmarinic Acid	197.0456, 179.0351, 161.0243, 135.0452, 123.0454	SZM	/
34*	4.42	[M-H]-	515.1167	-2.8	C_25_H_24_O_12_	Isochlorogenic acid C	515.1176, 353.0866, 335.0776, 299.0565, 255.0665, 191.0546, 179.0332, 173.0438, 135.0439	SZM CEC	/
**35**	4.74	[M+H]+	335.1152	-10.0	C_20_H_16_NO_4_	Isofagaridine	335.1132, 320.0890, 292.0923	LMZ	/
**36**	4.84	[M+H]+	334.1074	0.5	C_20_H_15_NO_4_	Des-N-methyl-chelerythrine	334.1081, 319.0848, 291.0901, 276.0669	LMZ	/
**37**	4.88	[M-H]-	825.4278	2.2	C_42_H_66_O_16_	Ilexpernoside D	825.4288, 663.3785, 601.3721, 487.3417, 340.0961, 173.0463	GM	/
**38**	5.19	[M+H]+	333.0996	-8.4	C_20_H_14_NO_4_	Sanguinarine	333.0987, 279.2324	LMZ	P
**39**	5.26	[M-H]-	167.035	0.6	C_8_H_8_O_4_	3, 5-O-dicaffeoyl quinic acid ethyl ester	167.0357, 123.0445, 108.0214	GM	P
**40**	5.3	[M-H]-	285.0405	2.4	C_15_H_10_O_6_	Luteolin or Kaempferol	285.0401, 267.1263, 201.0206, 175.0396, 151.0043, 133.0288, 121.0285	XXH GM	P
**41**	5.31	[M-H]-	911.501	0.8	C_47_H_76_O_17_	Oblonganoside M or Asprelloside H or Asprenol F	911.5017, 765.4459, 603.3880	GM	P
**42**	5.5	[M-H]-	809.4329	1.4	C_42_H_66_O_15_	Ilexside II	809.4326, 647.3808, 585.3844	GM	/
**43**	5.6	[M+H]+	190.0863	1.1	C_11_H_11_NO_2_	4-Methoxy-1-methyl-2-quinolone	190.0876, 175.0635, 147.0686, 130..0666	LMZ	/
**44**	5.9	[M-H]-	889.3897	-0.3	C_42_H_66_O_18_S	3-Methoxy-4,5-dihydroxybenzoic acid	889.3882, 809.4331, 254.9814	GM	P
**45**	6.02	[M+FA-H]-	725.3754	1.2	C_36_H_56_O_12_	2α,3β,19α-trihydroxy-urs-12-ene-24,28-dioic-28-O-β-D-glucopyranoside Rubusoside R1 or Ilexasprellanoside J	725.3753, 679.3680, 517.3127	GM	P
**46**	6.3	[M-H]-	925.3567	1.5	C_41_H_66_O_19_S_2_	Asprellcoside A or Asprellinoid B or Asprellinoid C or Ilexasprellanoside K	925.3581, 845.4007, 683.3492	GM	/
**47**	6.3	[M+H]+	230.0812	1.6	C_13_H_11_NO_3_	γ-fagarine	230.0825, 215.0582, 200.0343, 186.0558, 172.0394	LMZ	/
**48**	6.63	[M-H]-	663.375	0.6	C_36_H_56_O_11_	Ilexpublesnin E or Ilexoside XLV	663.3762, 587.3624, 543.3252, 487.3434, 437.3054, 393.3182, 175.0245, 129.0167	GM	P
**49**	6.66	[M-H]-	793.438	1.5	C_42_H_66_O_14_	Asprellanoside A	793.4402, 747.3409, 631.3838, 569.3862	GM	/
**50**	7	[M-H]-	871.3791	1.8	C_42_H_64_O_17_S	Asprelloside C	871.3822, 791.4209, 709.3269, 559.7910, 254.9832, 175.0243	GM	/
**51**	7.15	[M-H]-	261.1132	0.3	C_15_H_18_O_4_	(E)-methyl 3-(4-((E)-4-hydroxy-3-methylbut-2-enyloxy)phenyl)acryl or (Z)-methyl 3-(4-((E)-4-hydroxy-3-methylbut-2-enyloxy)phenyl)acrylate ate	261.1155, 217.1236, 188.1214, 173.1326, 158.9257, 105.0702	LMZ	P
**52**	7.23	[M-H]-	873.3948	-0.6	C_42_H_66_O_17_S	Ethyl chlorogenate	873.3967, 254.9816	GM	/
**53**	7.25	[M-H]-	507.2388	-0.1	C_30_H_36_O_7_	pungiolide A	507.2402 463.2488, 445.2392, 401.2510, 333.1349, 297.1132, 269.1181, 227.1431, 191.1073, 111.0452	CEC	P
**54**	8.77	[M-H]-	727.3369	-0.3	C_36_H_56_O_13_S	Vanilic acid or Asprelloside G or Ilexpuson F Asprelloside G, Ilexpuson	727.3352, 647.3811, 254.9814	GM	P
**55**	14.03	[M-H]-	253.2173	0.8	C_16_H_30_O_2_	Hexadecenoic acid	253.2172, 235.2073, 117.9311,	CEC	P
**56**	15.32	[M-H]-	255.233	-1.3	C_16_H_32_O_2_	Palmitic acid	255.2325, 237.2208, 116.9279	LMZ SZM CEC	P
**57**	17.15	[M-H]-	283.2643	-0.6	C_18_H_36_O_2_	Stearic acid	283.2633, 265.2531, 225.5383, 207.2494, 183.0136	CEC	P

**Note:** *Identified by comparison with reference standards. P: Prototypes detected in rat plasma. /: Not detected.

## Data Availability

The original contributions presented in the study are included in the article/supplementary material; further inquiries can be directed to the corresponding authors [X.H. and W.L.].

## LIST OF ABBREVIATIONS

ESI-MS: Electrospray Ionization-mass Spectrometer
LC–MS: Liquid Chromatography Paired with Mass Spectrometry
UHPLC-Q-TOF-MS: Ultra-high Performance Liquid Chromatography Quadrupole Time of Flight Mass Spectrometer
BPC: Base Peak Chromatograms
EIC: Extracted Ion Chromatogram
Glc: Glucose
Rha: Rhamnose
DI: Diagnostic Ion
NL: Neutral Loss
A: Aglycone
BPC: Base Peak Chromatogram
ESI-MS: Electro-spray Ionization-mass Spectrometry
IDA: Information-dependent Acquirement
HREIC: High Resolution Extracted Ion Chromatograms
