# Supplementary material for: Comprehensive Analysis of Chemical Ingredients of Waiganfengsha Granule and Absorbed Components in Rat Plasma Based on UHPLC-Q-TOF-MS
Source: Curr Drug Metab. 2024 May 23;25(3):205–19. doi: 10.2174/0113892002299899240515092703 (PMC11475105; doi:10.2174/0113892002299899240515092703)
Supplement: Supplementary file 1 [file CDM-25-205_SD1.pdf]

Supplementary Material

Comprehensive Analysis of Chemical Ingredients of Waiganfengsha Granule and Absorbed Components in Rat Plasma Based on UHPLC-Q-TOF-MS

Wei Wei[1,#,*], Liyuan Huang[2,#], Jun Huang[2], Jinhua Li[2], Yingying Qing[2], Xiaotao Hou[2,*] and Wen Liu[2,*]

[1]Guangxi Key Laboratory of Efficacy Study on Chinese Materia Medica, Guangxi University of Chinese Medicine, Nanning, Guangxi 530200, China; [2]Faculty of Pharmacy, Guangxi University of Chinese Medicine, Nanning, Guangxi, 530200, China

Table S1. The detail information of the components of WGFSG collected from literatures.

Compounds from XXH			
**NO**	**Compound Name**	**Formula**	**Chemical structure**
XXH1	Caffeic acid	$C_9H_8O_4$	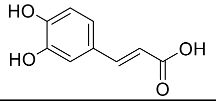
XXH2	Caffeic acid methyl ester	$C_{10}H_{10}O_4$	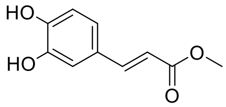
XXH3	3,4-di-0-caffeoyl quinic acid methyl ester	$C_{26}H_{26}O_{12}$	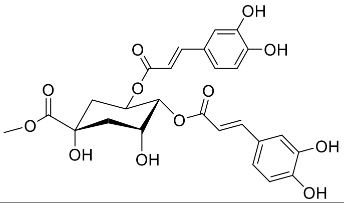
XXH4	3-0-caffeoyl quinic acid	$C_{16}H_{18}O_9$	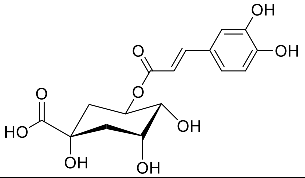
XXH5	3-0-caffeoyl quinic acid methyl ester	$C_{17}H_{20}O_9$	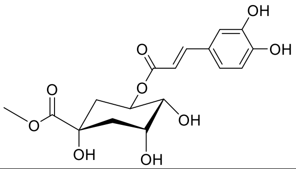
XXH6	5-0-caffeoyl quinic acid methyl ester	$C_{17}H_{20}O_9$	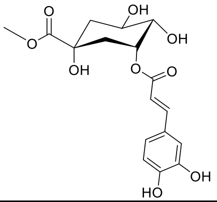
XXH7	4,5-di-0-caffeoyl quinic acid	$C_{26}H_{26}O_{12}$	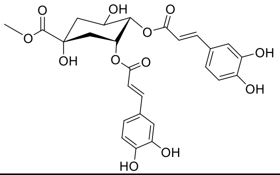

XXH8	(2S,3S,4R,10E)-2-((2R)-2-hydroxytetradecanoylamino]-10-octadecane-3,4-triol IV)	$C_{42}H_{83}NO_5$	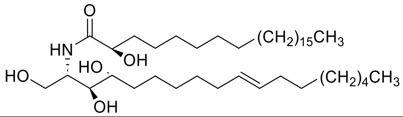
XXH9	indole-3-carboxylic acid	$C_9H_7NO_2$	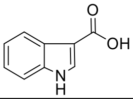
XXH10	diosmetin	$C_{16}H_{12}O_6$	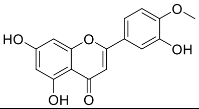
XXH11	apigenin	$C_{15}H_{10}O_5$	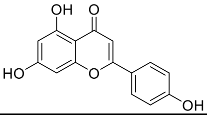
XXH12	luteolin	$C_{15}H_{10}O_6$	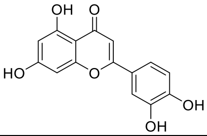
XXH13	(S)-N-benzoylalanine-(S)–2-benzamido-3-phenylpropyl ester	$C_{32}H_{30}O_4N_2$	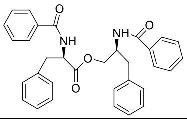
XXH14	stigmasterol	$C_{29}H_{48}O$	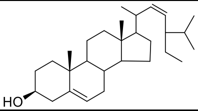
XXH15	α-spinasterol	$C_{29}H_{48}O$	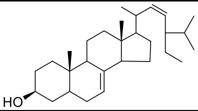
XXH16	n-heptadecanol	$C_{17}H_{36}O$	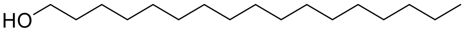
XXH17	n-tetratriacontanoic acid	$C_{34}H_{68}O_2$	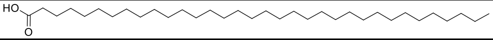
XXH18	n-te-tratriacontanoic	$C_{26}H_{52}O_4$	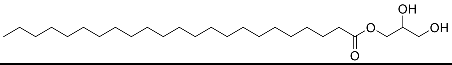
XXH19	stigmasterol-3-O-glucoside	$C_{35}H_{58}O_6$	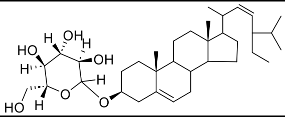
XXH20	baurenyl acetate	$C_{32}H_{52}O_2$	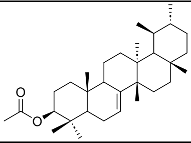
XXH21	epif riedelanol	$C_{30}H_{52}O$	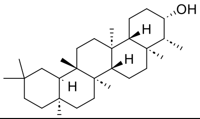
XXH22	friedelin	$C_{30}H_{50}O$	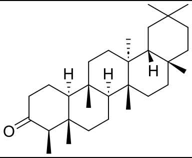
XXH23	20(30)-taraxastene-3β,21α-diol	$C_{30}H_{50}O_2$	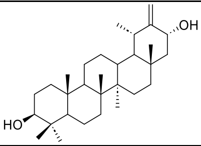

XXH24	3,5-dicaffeoylquinic acid	$C_{25}H_{24}O_{12}$	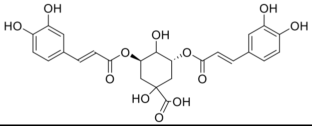
XXH25	3,4-dicaffeoylquinic acid	$C_{25}H_{24}O_{12}$	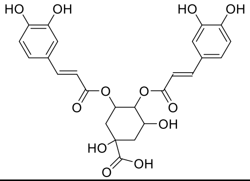
XXH26	4,5-dicaffeoylquinic acid	$C_{25}H_{24}O_{12}$	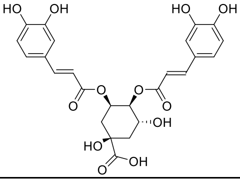
XXH27	cryptochlorogenic acid	$C_{16}H_{18}O_{9}$	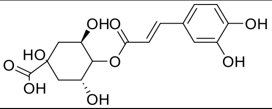
XXH28	chlorogenic acid	$C_{16}H_{18}O_{9}$	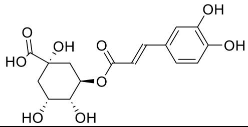
XXH29	neochlorogenic acid	$C_{16}H_{18}O_{9}$	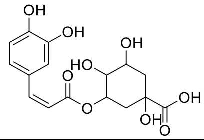
XXH30	isoquercetin	$C_{21}H_{20}O_{12}$	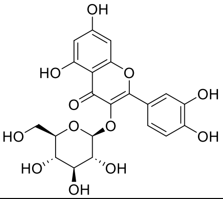
Compounds from LMZ			
NO	Compound Name	Formula	Chemical structure
LMZ1	oxynitidine	$C_{21}H_{17}NO_{5}$	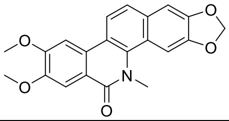
LMZ2	oxychelerythrine	$C_{21}H_{17}NO_{5}$	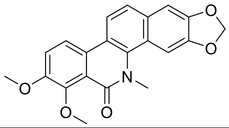
LMZ3	6-ethoxychelerythrine	$C_{23}H_{23}NO_{5}$	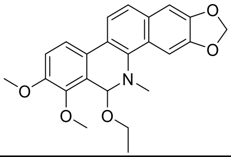
LMZ4	dihydronitidine	$C_{21}H_{19}NO_{4}$	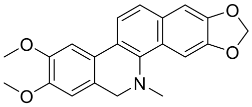
LMZ5	7-demethyl-6-methoxy-5, 6-dihydrochelerythrine	$C_{21}H_{19}NO_{5}$	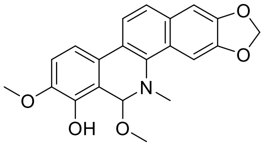

LMZ6	dihydrochelerythrine	$C_{21}H_{19}NO_4$	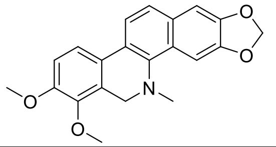
LMZ7	8-(1′-hydroxy)ethyldihydrochelerythrine	$C_{23}H_{23}NO_5$	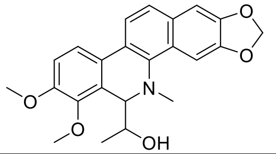
LMZ8	oxyavicine	$C_{20}H_{13}NO_5$	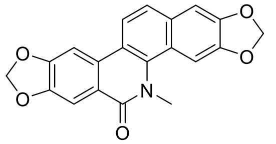
LMZ9	8-methoxydihydrochelerythrine	$C_{22}H_{21}NO_5$	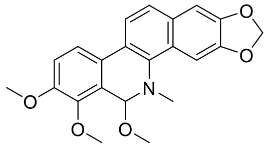
LMZ10	8-hydroxydihydrochelerythrine	$C_{21}H_{19}NO_5$	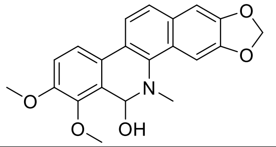
LMZ11	dihydrochelerythrinyl-8-acetaldehyde	$C_{23}H_{21}NO_5$	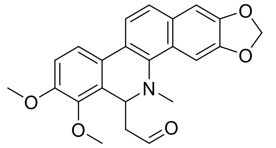
LMZ12	oxyterihanine	$C_{20}H_{15}NO_5$	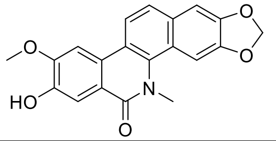
LMZ13	8-acetonyldihydrofagaridine	$C_{23}H_{21}NO_5$	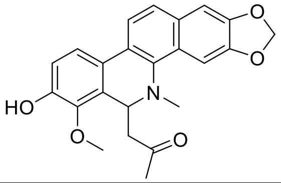
LMZ14	8-acetonyldihydronitidine	$C_{24}H_{23}NO_5$	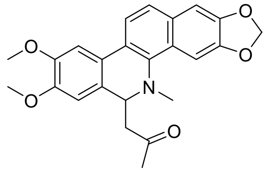
LMZ15	8-acetonyldihydrochelerythrine	$C_{24}H_{23}NO_5$	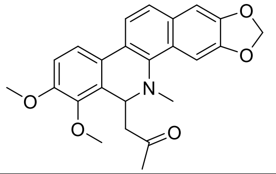
LMZ16	8-acetonyldihydroavicine	$C_{23}H_{19}NO_5$	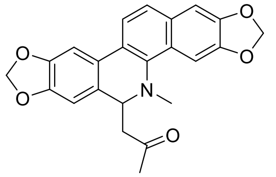

LMZ17	6-methoxy-5,6-dihydronitidine	$C_{22}H_{21}NO_5$	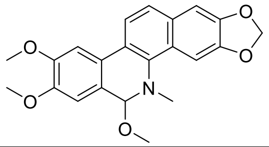
LMZ18	bocconoline	$C_{22}H_{21}NO_5$	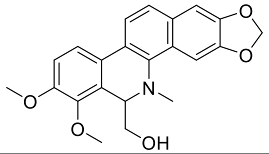
LMZ19	6β-hydroxymethyldihydronitidine	$C_{22}H_{21}NO_5$	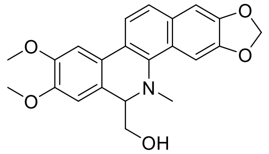
LMZ20	8-demethyloxychelerythrine	$C_{20}H_{15}NO_5$	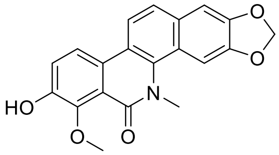
LMZ21	6-carboxymethyldihydrochelerythrine	$C_{23}H_{21}NO_6$	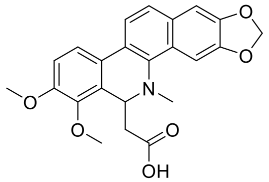
LMZ22	nitidine	$C_{21}H_{18}NO_4^+$	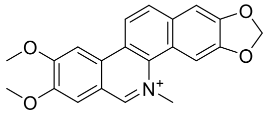
LMZ23	chelerythrine	$C_{21}H_{18}NO_4^+$	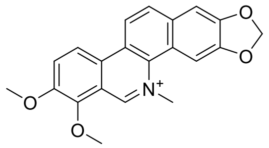
LMZ24	isofagaridine	$C_{20}H_{16}NO_4^+$	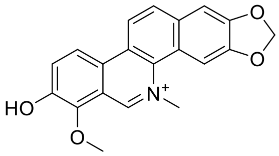
LMZ25	8-methoxynorchelerythrine	$C_{22}H_{20}NO_5^+$	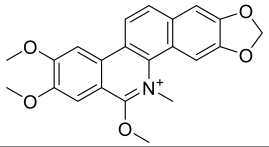
LMZ26	sanguinarine	$C_{20}H_{14}NO_4^+$	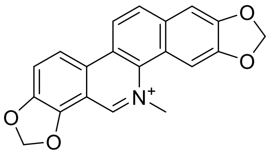
LMZ27	8-methoxysanguinarine	$C_{21}H_{16}NO_5^+$	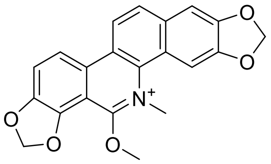
LMZ28	avicine	$C_{20}H_{14}NO_4^+$	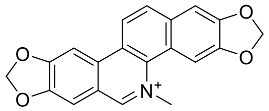

LMZ29	7-methoxy-8-demethoxynitidine	$C_{21}H_{18}NO_4^+$	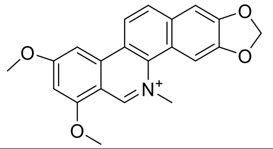
LMZ30	des-N-methyl-chelerythrine	$C_{20}H_{15}NO_4$	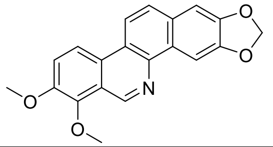
LMZ31	decarine	$C_{19}H_{13}NO_4$	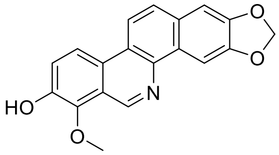
LMZ32	6,7,8-trimethoxy-2,3-methylendioxybenzophenantridine	$C_{21}H_{17}NO_5$	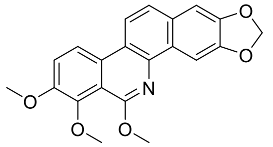
LMZ33	rhoifoline A	$C_{20}H_{13}NO_5$	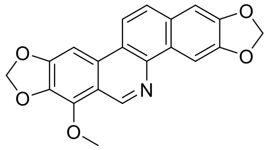
LMZ34	des-N-methylnitidine	$C_{20}H_{15}NO_4$	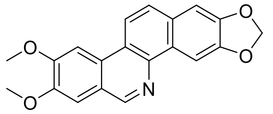
LMZ35	8-methoxyisodecarine	$C_{20}H_{15}NO_5$	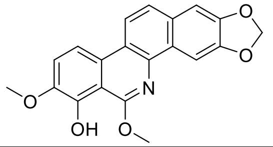
LMZ36	zanthoxyline	$C_{19}H_{13}NO_4$	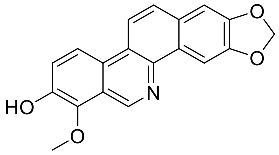
LMZ37	rhoifoline B	$C_{21}H_{17}NO_5$	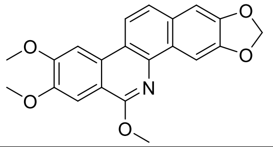
LMZ38	arnottianamide	$C_{21}H_{19}NO_6$	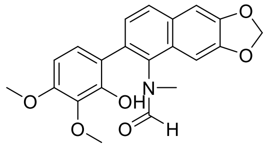
LMZ39	isoarnottianamide	$C_{21}H_{19}NO_6$	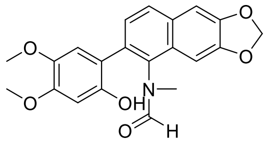
LMZ40	integriamide	$C_{20}H_{15}NO_6$	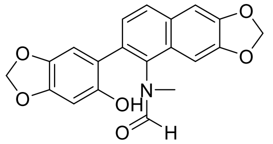

LMZ41	nitidumtone A	$C_{45}H_{40}N_2O_9$	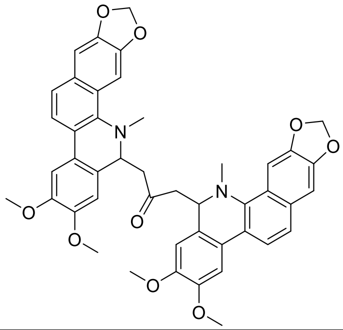
LMZ42	zanthomuurolanine	$C_{37}H_{45}NO_5$	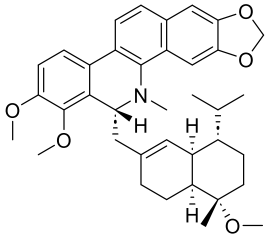
LMZ43	epi-zanthomuurolanine	$C_{37}H_{45}NO_5$	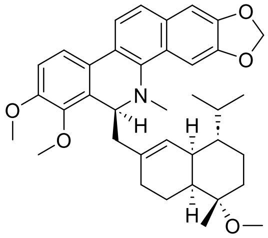
LMZ44	zanthocadinanine A	$C_{37}H_{45}NO_5$	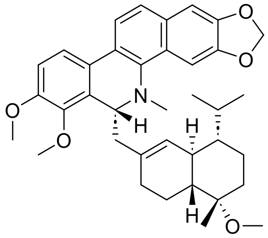
LMZ45	zanthocadinanine B	$C_{37}H_{45}NO_5$	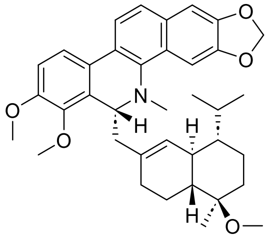
LMZ46	epizanthocadinanine B	$C_{37}H_{45}NO_5$	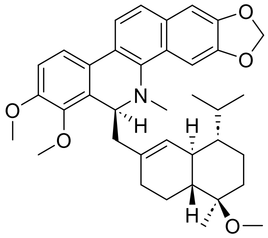
LMZ47	epizanthocadinanine A	$C_{37}H_{45}NO_5$	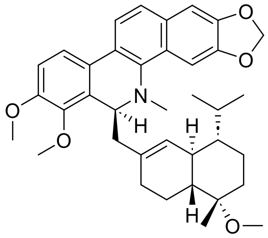

LMZ48	8-(2′-cyclohexanone)-7,8-dihydrochelerythrine	$C_{27}H_{27}NO_5$	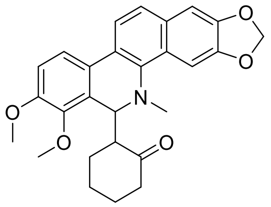
LMZ49	turraeanthin A	$C_{21}H_{19}NO_6$	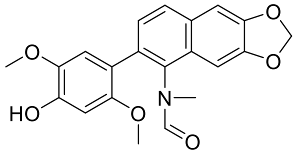
LMZ50	zanthonitiside C	$C_{27}H_{31}NO_{11}$	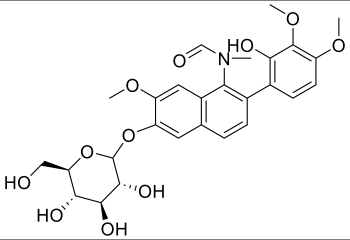
LMZ51	zanthonitiside D	$C_{26}H_{29}NO_{11}$	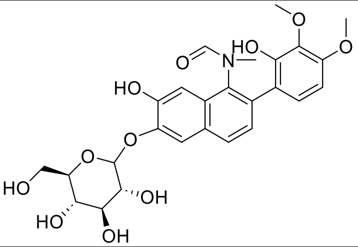
LMZ52	zanthocadinanine C	$C_{36}H_{41}NO_4$	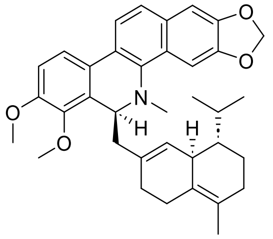
LMZ53	skimmianine	$C_{14}H_{13}NO_4$	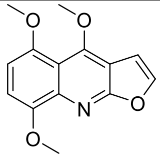
LMZ54	dictamnine	$C_{12}H_9NO_2$	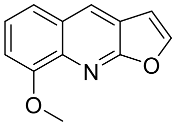
LMZ55	haplopine	$C_{13}H_{11}NO_4$	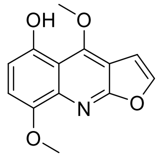
LMZ56	γ-fagarine	$C_{13}H_{11}NO_3$	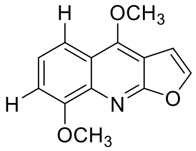

LMZ57	5-methoxydictamnine	$C_{13}H_{11}NO_3$	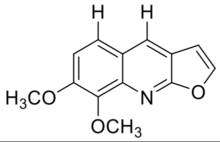
LMZ58	robustine	$C_{12}H_9NO_3$	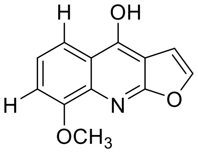
LMZ59	4-hydroxy-7,8-dimethoxyfuranquinoline	$C_{13}H_{11}NO_4$	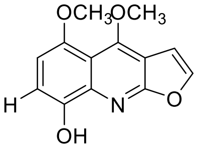
LMZ60	flindersine	$C_{14}H_{13}NO_2$	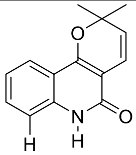
LMZ61	N-Methylflindersine	$C_{15}H_{15}NO_2$	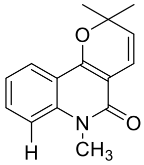
LMZ62	zanthobungeanine	$C_{16}H_{17}NO_3$	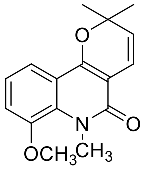
LMZ63	edulinine	$C_{16}H_{21}NO_4$	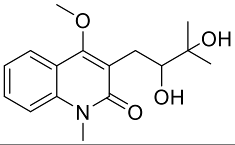
LMZ64	4-methoxy-1-methyl-2-quinolone	$C_{11}H_{11}NO_2$	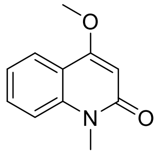
LMZ65	zanthodioline	$C_{16}H_{19}NO_5$	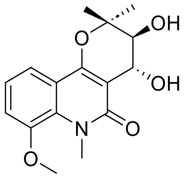
LMZ66	(+)-zanthonitidine A	$C_{23}H_{21}NO_8$	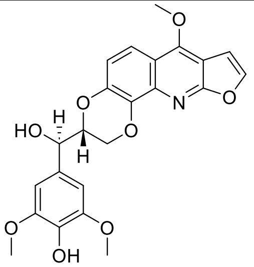

LMZ67	(−)-zanthonitidine A	$C_{23}H_{21}NO_8$	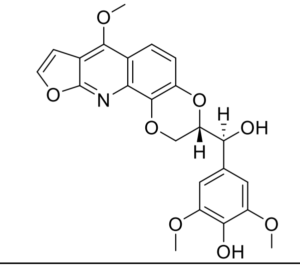
LMZ68	zanthonitidine B	$C_{27}H_{26}N_2O_5$	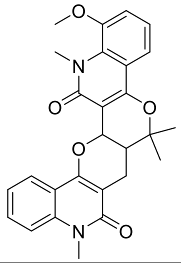
LMZ69	isoplatydesmine	$C_{15}H_{17}NO_3$	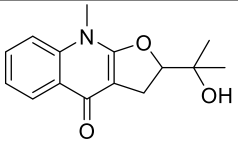
LMZ70	zanthonitiside A	$C_{21}H_{27}NO_8$	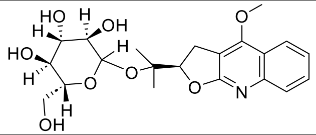
LMZ71	zanthonitiside B	$C_{21}H_{27}NO_8$	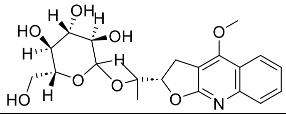
LMZ72	zanthonitiside I	$C_{21}H_{27}NO_8$	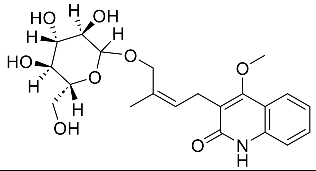
LMZ73	ribalinine	$C_{15}H_{17}NO_3$	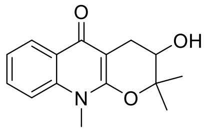
LMZ74	jatrorrhizine	$C_{20}H_{20}NO_4^+$	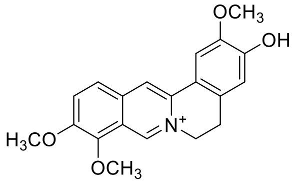
LMZ75	columbamine	$C_{20}H_{20}NO_4^+$	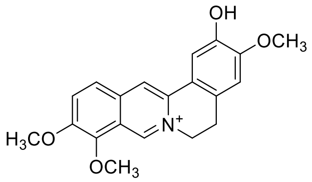
LMZ76	palmatine	$C_{21}H_{22}NO_4^+$	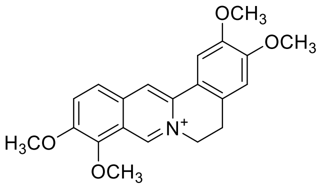

LMZ77	epiberberine	$C_{20}H_{18}NO_4^+$	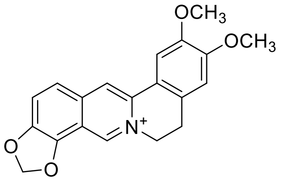
LMZ78	liriodenine	$C_{17}H_9NO_3$	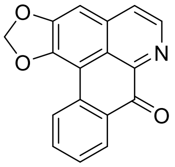
LMZ79	N-acetyldehydroanonaine	$C_{19}H_{15}NO_3$	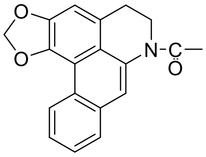
LMZ80	N-acetylanonaine	$C_{19}H_{17}NO_3$	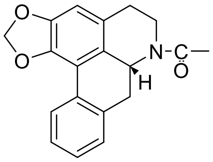
LMZ81	magnoflorine	$C_{20}H_{24}NO_4^+$	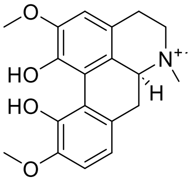
LMZ82	4,5-dihydroxy-1-methyl-3-oxo-2-(trichloromethyl)-3H-indolium	$C_{10}H_7Cl_3NO_3^+$	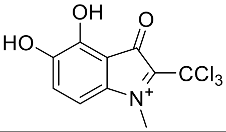
LMZ83	Indole-3-carboxaldehyde	C_9H_7NO	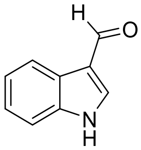
LMZ84	methyl 7-(b-d-mannopyranosyloxy)-1H-indole-2-carboxylate	$C_{13}H_{13}NO_4$	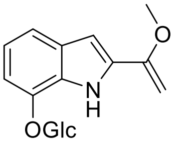
LMZ85	2-methyl-1H-indol-7-yl b-d-mannopyranoside	$C_{15}H_{19}NO_6$	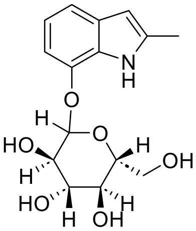
LMZ86	amaroridine	$C_{15}H_{10}N_2O_2$	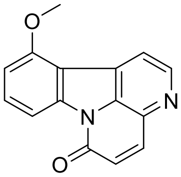

LMZ87	thalifoline	$C_{11}H_{13}NO_3$	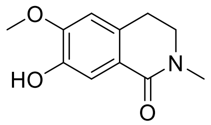
LMZ88	α-allocryptopine	$C_{21}H_{23}NO_5$	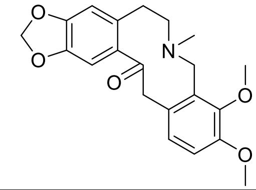
LMZ89	5,7-dimethoxycoumarin	$C_{11}H_{10}O_4$	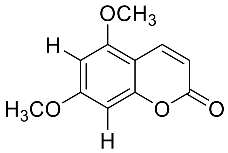
LMZ90	5,7,8-trimethoxycoumarin	$C_{12}H_{12}O_5$	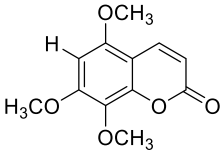
LMZ91	5,7-dimethoxy-8-prenyloxycoumarin	$C_{16}H_{18}O_5$	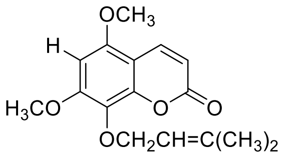
LMZ92	5,6,7-trimethoxycoumarin	$C_{12}H_{12}O_5$	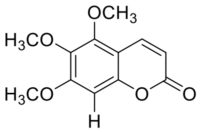
LMZ93	aesculetin dimethyl ether	$C_{11}H_{10}O_4$	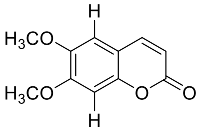
LMZ94	6,7,8-trimethoxycoumarin	$C_{12}H_{12}O_5$	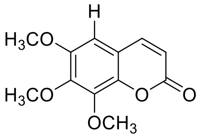
LMZ95	scopoletin	$C_{10}H_8O_4$	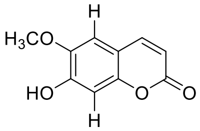
LMZ96	5-geranyloxy-7-methoxycoumarin	$C_{20}H_{24}O_4$	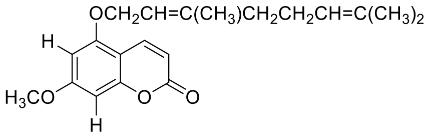
LMZ97	toddalolactone	$C_{16}H_{20}O_6$	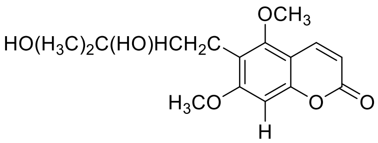
LMZ98	toddanone	$C_{16}H_{18}O_5$	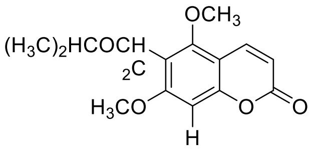

LMZ99	toddaculine	$C_{16}H_{18}O_4$	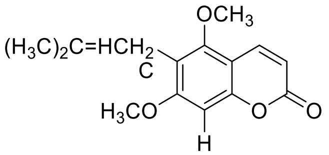
LMZ100	7,8-dimethoxycoumarin	$C_{11}H_{10}O_4$	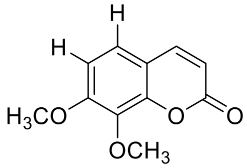
LMZ101	mexoticin	$C_{16}H_{21}O_7$	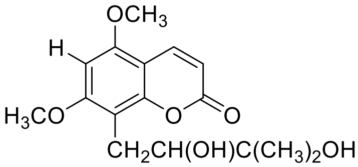
LMZ102	7-hydroxycoumarin	$C_9H_6O_3$	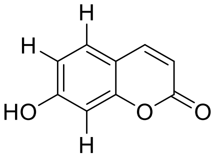
LMZ103	isopranferin	$C_{19}H_{24}O_6$	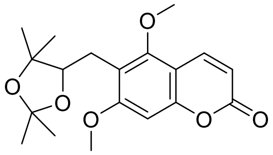
LMZ104	isopimpinellin	$C_{13}H_{10}O_5$	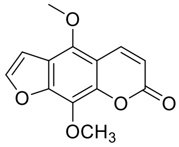
LMZ105	phellopterin	$C_{17}H_{16}O_5$	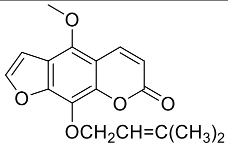
LMZ106	5-methoxymarmesin	$C_{15}H_{16}O_6$	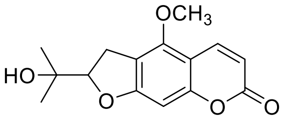
LMZ107	marmesin	$C_{14}H_{14}O_4$	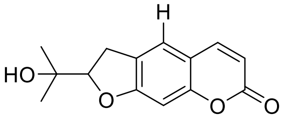
LMZ108	l-asarinin	$C_{20}H_{18}O_6$	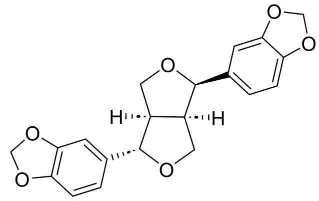
LMZ109	l-sesamin	$C_{20}H_{18}O_6$	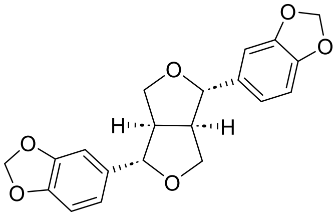

LMZ110	horsfieldin	$C_{20}H_{20}O_6$	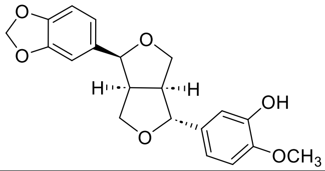
LMZ111	d-episesamin	$C_{20}H_{18}O_6$	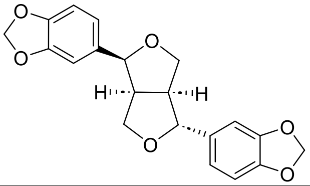
LMZ112	piperitol-3,3-dimethylallylether	$C_{24}H_{28}O_6$	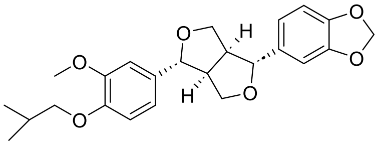
LMZ113	xanthoxylol-3,3-dimethylallylether	$C_{24}H_{28}O_6$	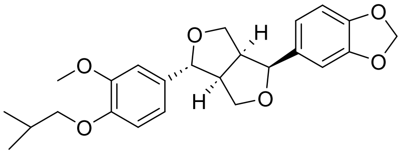
LMZ114	savinin	$C_{20}H_{16}O_6$	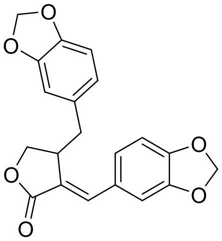
LMZ115	2,3-bis(3,4-methylenedioxybenzyl)but-2-en-4-olide	$C_{20}H_{16}O_6$	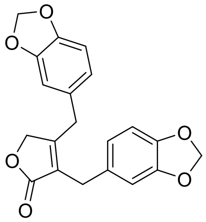
LMZ116	l-syringaresinol	$C_{22}H_{26}O_8$	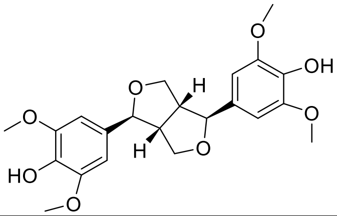
LMZ117	Prestegane B	$C_{20}H_{22}O_6$	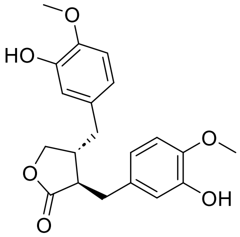
LMZ118	5,5′-dimethoxylariciresinol	$C_{22}H_{28}O_8$	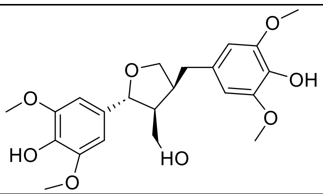

LMZ119	episyringaresinol	$C_{24}H_{30}O_8$	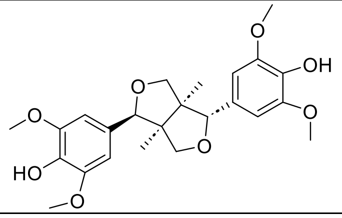
LMZ120	(7R,8S)-4,4′-dihydroxy-3,7,3′-trimethoxy-8,1′–7′,8′,9′-trinor-neolignan-9-olethanol	$C_{18}H_{22}O_6$	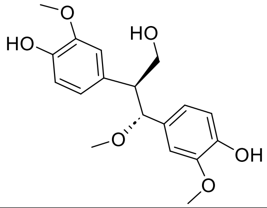
LMZ121	(7S,8R)-4,4′-dihydroxy-3,7,3′-trimethoxy-8,1′–7′,8′,9′-trinor-neolignan-9-olethanol	$C_{18}H_{22}O_6$	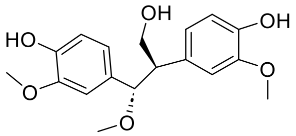
LMZ122	xylobuxin	$C_{21}H_{22}O_7$	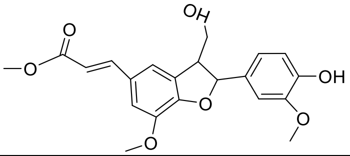
LMZ123	hesperidin	$C_{28}H_{34}O_{15}$	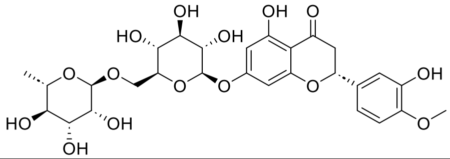
LMZ124	diosmin	$C_{28}H_{32}O_{15}$	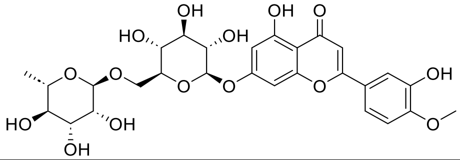
LMZ125	vitexin	$C_{21}H_{20}O_{10}$	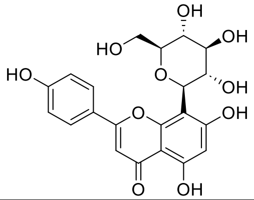
LMZ126	apigenin	$C_{15}H_{10}O_5$	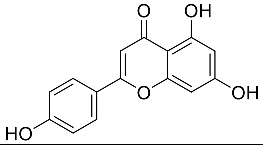
LMZ127	2,6-dimethoxy-1,4-benzoquinone	$C_8H_8O_4$	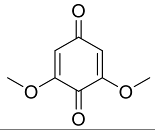
LMZ128	6-diisopropyl-2,5-piperazinedione	$C_{10}H_{18}N_2O_2$	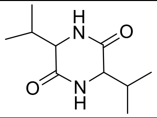

LMZ129	cyclo-(Leu-Leu-Leu-Leu-Ile)	$C_{30}H_{55}N_5O_5$	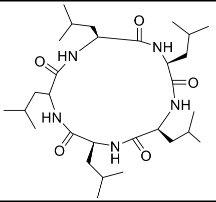
LMZ130	β-sitosterol	$C_{29}H_{50}O$	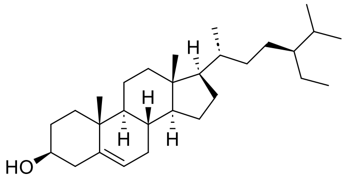
LMZ131	stigmast-9(11)-en-3-ol	$C_{29}H_{50}O$	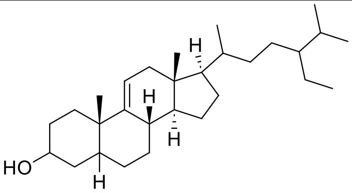
LMZ132	β-daucosterol	$C_{35}H_{60}O_6$	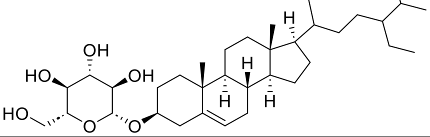
LMZ133	syringic acid	$C_9H_{10}O_5$	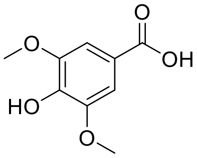
LMZ134	4-hydroxybenzoic acid	$C_7H_6O_3$	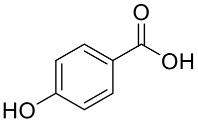
LMZ135	4-(3-methylbut-2-enyloxy)benzoic acid	$C_{12}H_{14}O_3$	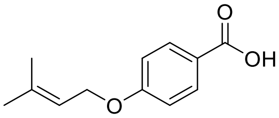
LMZ136	(Z)-3-(2, 3, 4-trimethoxyphenyl)acrylic acid	$C_{12}H_{14}O_5$	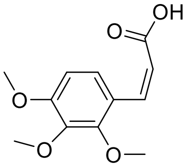
LMZ137	4-hydroxy-N-methylproline	$C_6H_{11}NO_3$	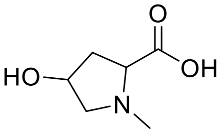
LMZ138	palmitic acid	$C_{16}H_{32}O_2$	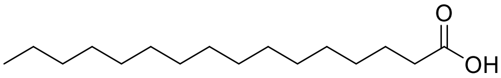
LMZ139	neoherculin	$C_{16}H_{25}NO$	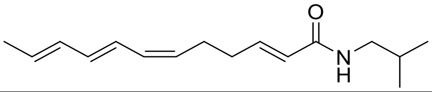
LMZ140	zanthoxylumamide A	$C_{16}H_{25}NO_3$	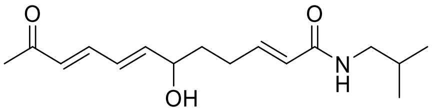

LMZ141	zanthoxylumamide B	$C_{16}H_{25}NO_3$	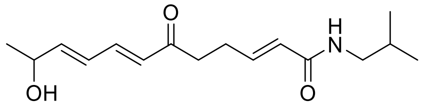
LMZ142	zanthoxylumamide C	$C_{16}H_{27}NO_3$	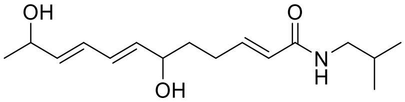
LMZ143	zanthoxylumamide D	$C_{18}H_{29}NO_3$	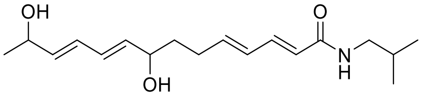
LMZ144	(2E,6E,8E)-N-(2-methylpropyl)-10-oxo-2,6,8-decatrienamide	$C_{14}H_{21}NO_2$	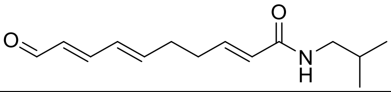
LMZ145	benzamide	C_7H_7NO	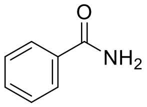
LMZ146	(E)-4-(4-hydroxy-3-methylbut-2-enyloxy)benzaldehyde	$C_{12}H_{14}O_3$	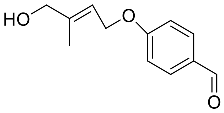
LMZ147	(E)-methyl 3-(4-((E)-4-hydroxy-3-methylbut-2-enyloxy)phenyl)acrylate	$C_{15}H_{18}O_4$	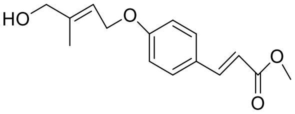
LMZ148	(Z)-methyl 3-(4-((E)-4-hydroxy-3-methylbut-2-enyloxy)phenyl)acrylate	$C_{15}H_{18}O_4$	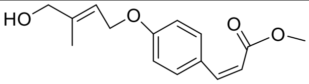
Compounds from TKS			
NO	Compound Name	Formula	Chemical structure
TKS1	α-amyrin acetate	$C_{32}H_{52}O_2$	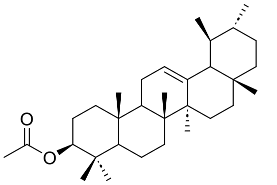
TKS2	lupeol acetate	$C_{32}H_{52}O_2$	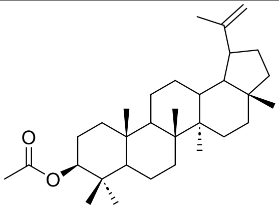
TKS3	11-ethoxy-3-acetyl-12-ursene-3-ol	$C_{34}H_{56}O_3$	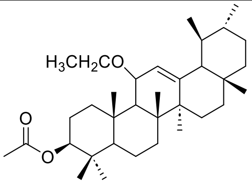
TKS4	11-oxo-α-amyrin acetate	$C_{32}H_{50}O_3$	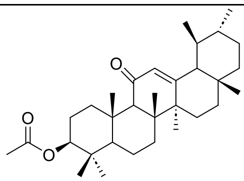

TKS5	α-amyrin	$C_{30}H_{50}O$	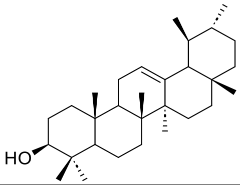
TKS6	lupeol	$C_{30}H_{50}O$	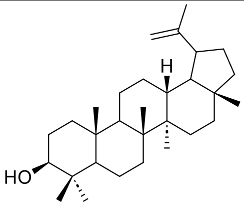
TKS7	24-methylenecycloartanol	$C_{31}H_{52}O$	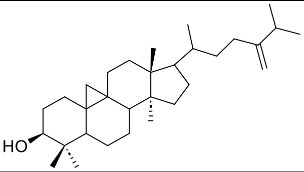
TKS8	cycloart-23-ene-3β, 25-diol	$C_{30}H_{50}O_2$	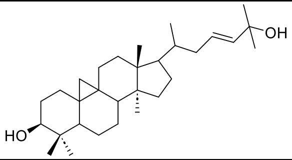
TKS9	β-sitosterol	$C_{29}H_{50}O$	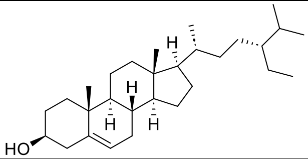
TKS10	periplogenin digitoxoside	$C_{29}H_{44}O_7$	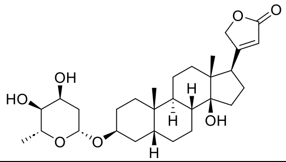
TKS11	griffithigenin	$C_{23}H_{32}O_5$	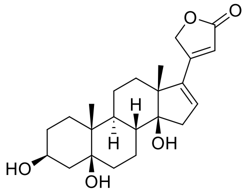
TKS12	cycloartan-3, 24, 25-triol	$C_{30}H_{52}O_3$	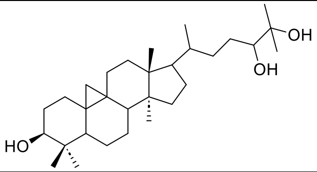
TKS13	digitoxogenin	$C_{23}H_{34}O_4$	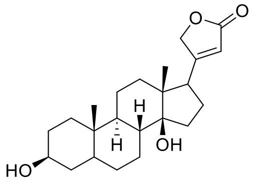
TKS14	16-O-acetylgitoxigenin	$C_{25}H_{36}O_6$	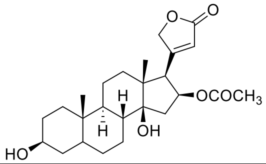

TKS15	periplogenin	$C_{23}H_{34}O_5$	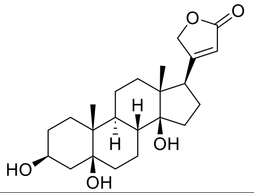
TKS16	16-O-acetyl-hydroxyperiplogenin	$C_{25}H_{36}O_7$	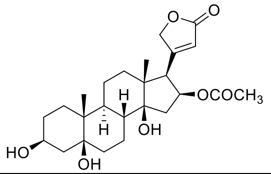
TKS17	corchorusosede C	$C_{35}H_{54}O_{13}$	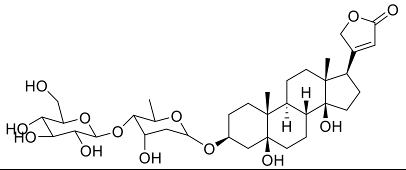
TKS18	periplogenin glucoside	$C_{29}H_{44}O_{10}$	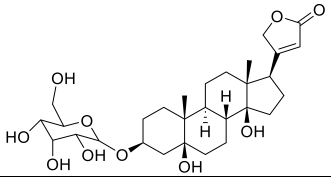
TKS19	griffithoside A	$C_{29}H_{42}O_{10}$	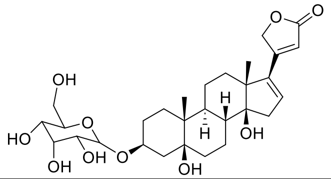
TKS20	3β,7β,12β-trihydroxy-4,4,14α-trimethyl-11,15-dioxo-5α-chol-8-en-24-oic acid	$C_{27}H_{40}O_7$	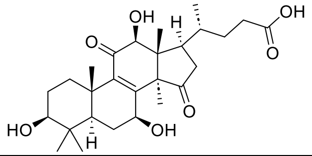
TKS21	Methyl lucidenate P	$C_{30}H_{44}O_8$	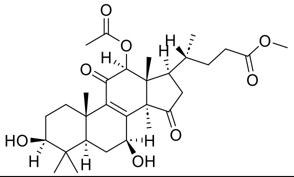
TKS22	Methyl lucidenate K	$C_{28}H_{38}O_7$	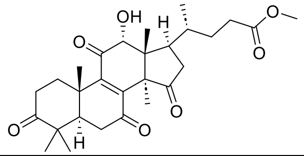
TKS23	Griffithii X	$C_{28}H_{42}O_5$	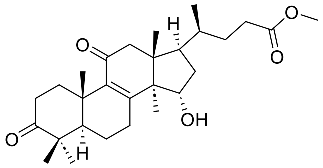
TKS24	Dammar-20,24-dien-3β-ol	$C_{30}H_{50}O$	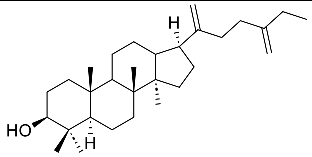

TKS25	ursolic acid	$C_{30}H_{48}O_3$	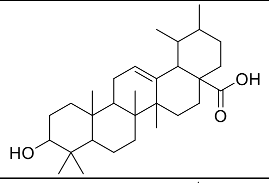
TKS26	23-hydroxy-ursolic acid	$C_{30}H_{48}O_4$	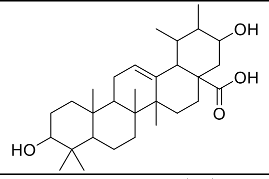
TKS27	Arjunolic acid	$C_{30}H_{48}O_5$	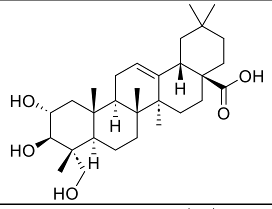
TKS28	3β,6β,23-trihydroxyolean-12-en-28-oic acid	$C_{30}H_{48}O_5$	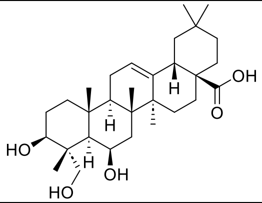
TKS29	3β,6α,23-trihydroxyolean-12-en-28-oic acid	$C_{30}H_{48}O_5$	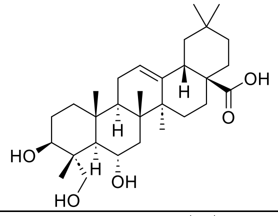
TKS30	2α,3β,23-trihydroxyurs-12-en-28-oic acid	$C_{30}H_{48}O_5$	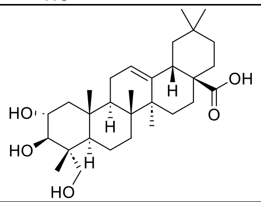
TKS31	23-hydroxyerythrodiol	$C_{30}H_{50}O_3$	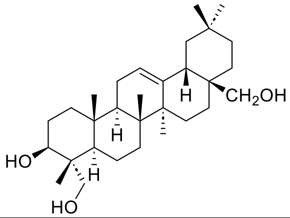
TKS32	belleric acid	$C_{30}H_{48}O_6$	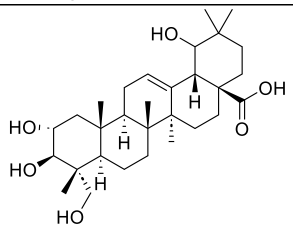

TKS33	(24S)-24-ethylcholesta-3β,5α,6β-triol	$C_{29}H_{52}O_3$	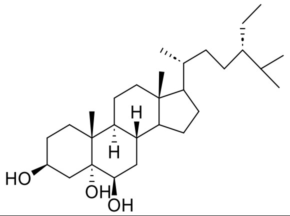
TKS34	Daucosterol	$C_{35}H_{60}O_6$	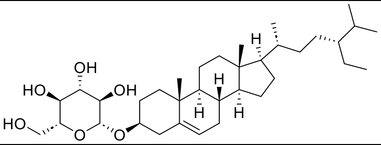
TKS35	7α-hydroxy sitosterol-3-O-β-glucoside	$C_{35}H_{60}O_7$	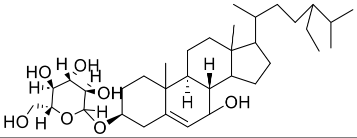
TKS36	3α-periplogenin	$C_{23}H_{34}O_5$	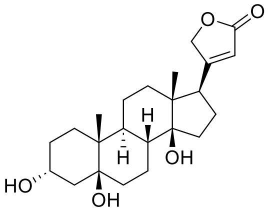
TKS37	3,5-Dimethoxy-4-hydroxybenzaldehyde	$C_8H_8O_4$	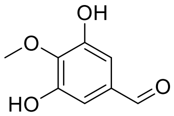
TKS38	Vanillin	$C_8H_8O_3$	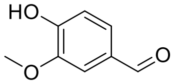
TKS39	ferulic acid	$C_{10}H_{10}O_4$	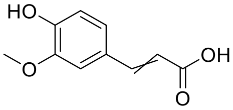
TKS40	4-hydroxy-3,5-dimethoxybenzaldehyde	$C_9H_{10}O_4$	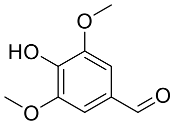
TKS41	ar-turmerone	$C_{15}H_{20}O$	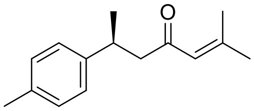
TKS42	griffithin	$C_{25}H_{36}O_7$	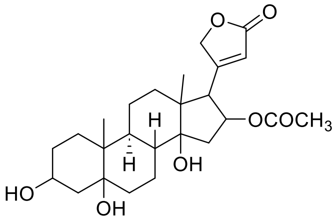
TKS43	digitoxigenin	$C_{23}H_{34}O_4$	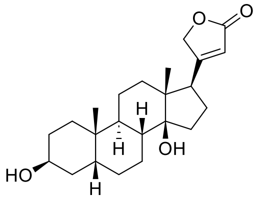

TKS44	acovenosigenin A	$C_{23}H_{34}O_5$	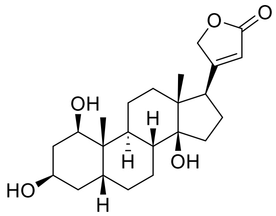
TKS45	daucosterol palmitate	$C_{51}H_{90}O_7$	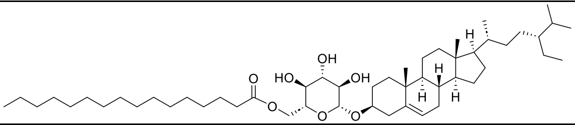
TKS46	β-daucosterol	$C_{35}H_{60}O_6$	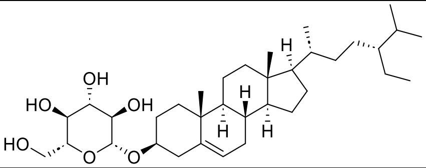
TKS47	physcion	$C_{16}H_{12}O_5$	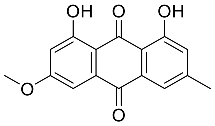
TKS48	scopoletin	$C_{10}H_8O_4$	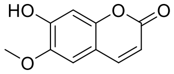
TKS49	daphnoretin	$C_{19}H_{12}O_7$	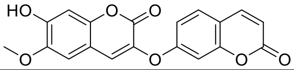
TKS50	salicylic acid	$C_7H_6O_3$	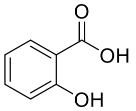
TKS51	syringic acid	$C_9H_{10}O_5$	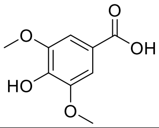
TKS52	hesperidin	$C_{28}H_{34}O_{15}$	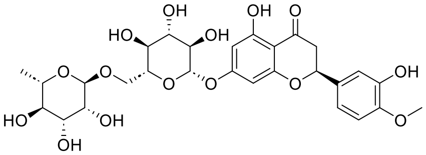
TKS53	Periploxin-3β-Acetate	$C_{25}H_{36}O_5$	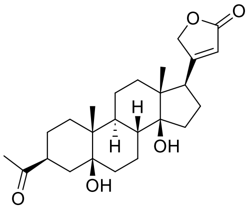
TKS54	Usagin	$C_{23}H_{34}O_4$	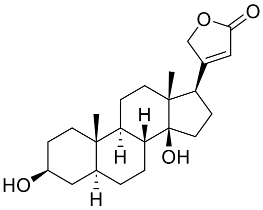
TKS55	α-Aromatic resin alcohol tridecanoate	$C_{43}H_{74}O_2$	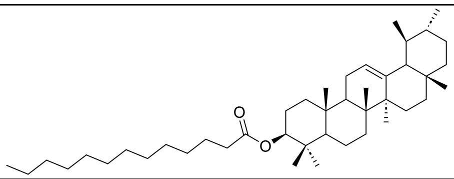

TKS56	9,19-Cyclo Altin-25-en-3 β, 24R diol	$C_{30}H_{51}O_2$	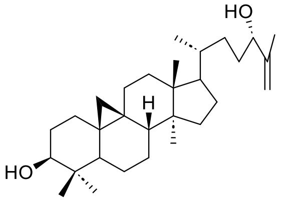
TKS57	9,19-Cyclo Altin-25-en-3 β, 24S diol	$C_{30}H_{50}O_2$	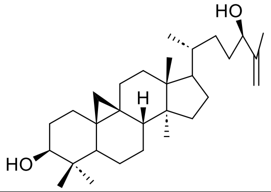
TKS58	Cyclocineol	$C_{30}H_{50}O$	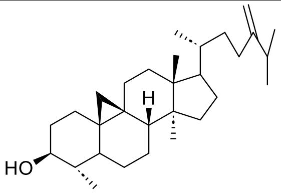
TKS59	9,19-Cyclo Altin-23E-en-3 β, 25-diol	$C_{30}H_{50}O_2$	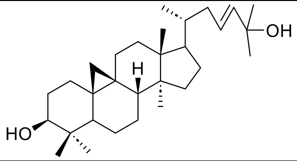
TKS60	25-Methoxy-9,19-Cycloartetin-23E-en-3 β-alcohol	$C_{31}H_{52}O_2$	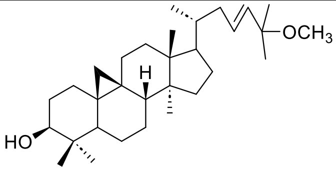
TKS61	11α, 12α-epoxytaraxer-14-en-3β-acetate	$C_{32}H_{50}O_2$	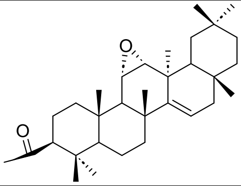
TKS62	Oleanolic Acid	$C_{30}H_{48}O_3$	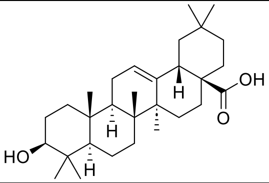
TKS63	3β,5β,14β-trihydroxyl-card-16,20(22)-dienolide	$C_{23}H_{32}O_5$	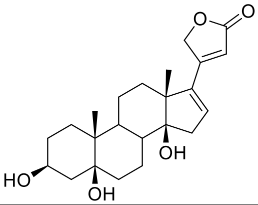
TKS64	3-O-β-d-glucopyranosyl-5β,14β-dihydroxyl-card-16,20(22)-dienolide	$C_{29}H_{42}O_{10}$	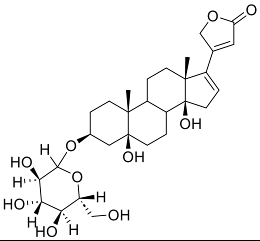

TKS65	digitogenin	$C_{27}H_{44}O_5$	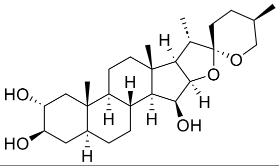
TKS66	16-O-acetylgitoxigenin	$C_{25}H_{36}O_6$	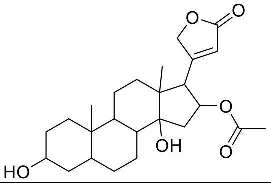
TKS67	16-O-acetylperiplogenin	$C_{23}H_{34}O_5$	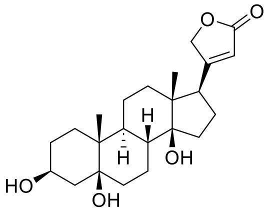
TKS68	periplogenin-3-O-β-d-glucopyranoside	$C_{29}H_{44}O_{10}$	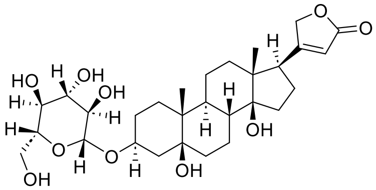
TKS69	periplogenin-3-O-β-d-glucopyranosyl-(1 → 4)-O-β-d-digitoxopyranoside	$C_{35}H_{54}O_{13}$	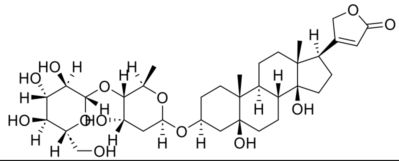
TKS70	periplogenin 3- O-[O-β-D-glucopyranosyl-(1 → 4)-O-β-D-glucopyranosyl-(1 → 4)-β-D-cymaropyranoside]	$C_{42}H_{66}O_{18}$	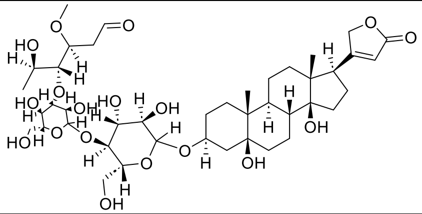
TKS71	Periplogenin 3-O-[O-β-d-glucopyranosyl-(1→6)-O-β-d-glucopyranosyl-(1→4)-2-O-acetyl-β-d-digitalopyranoside]	$C_{44}H_{68}O_{20}$	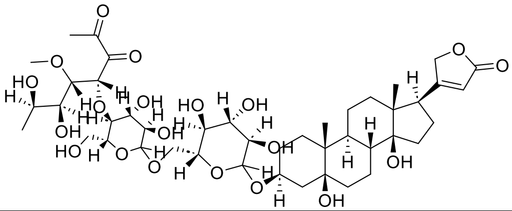
TKS72	Periplogenin 3-O-[O-β-d-glucopyranosyl-(1→4)-O-βd-glucopyranosyl-(1→4)-β-d-digitoxopyranoside]	$C_{41}H_{64}O_{18}$	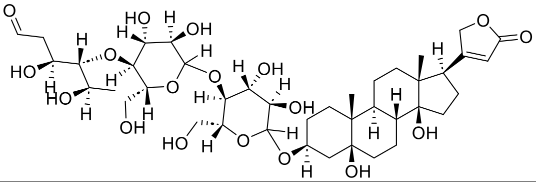

TKS73	Acovenosigenin A 3-O-[O-β-d-glucopyranosyl-(1→6)-O-β-d-glucopyranosyl-(1→4)-β-d-cymaropyranoside]	$C_{42}H_{66}O_{18}$	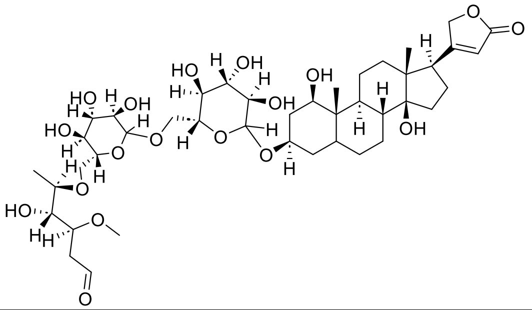
TKS74	Acovenosigenin A 3-O-[O-β-d-glucopyranosyl-(1→6)-O-β-d-glucopyranosyl-(1→4)-2-O-acetyl-β-d-digitalopyranoside]	$C_{44}H_{68}O_{20}$	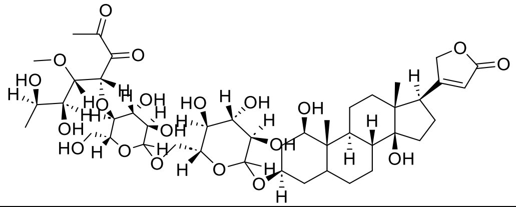
TKS75	16-O-acetyl-hydroxyacovenosigenin 3-O-[O-β-d-glucopyranosyl-(1→6)-O-β-d-glucopyranosyl-(1→4)-2-O-acetyl-β-d-digitalopyranoside]	$C_{46}H_{70}O_{22}$	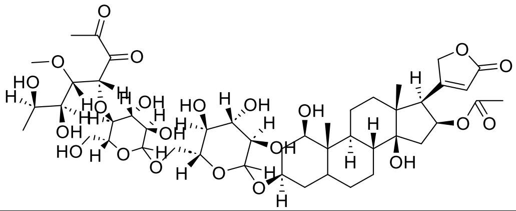
TKS76	Acovenosigenin A 3-O-[O-β-d-glucopyranosyl-(1→6)-O-β-d-glucopyranosyl-(1→4)-O-β-d-digitalopyranosyl-(1→4)-β-d-cymaropyranoside]	$C_{49}H_{78}O_{22}$	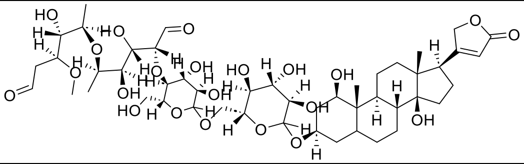
TKS77	Acovenosigeninβ-D-glucoside	$C_{29}H_{44}O_{10}$	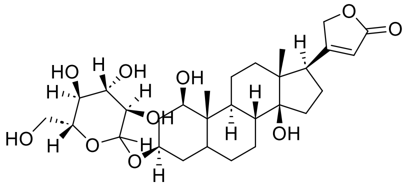
TKS78	Digitoxigenin sophoroside	$C_{35}H_{54}O_{14}$	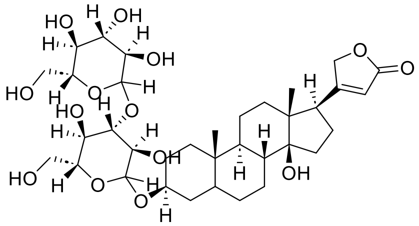
TKS79	Digitoxigenin gentiobioside	$C_{35}H_{54}O_{14}$	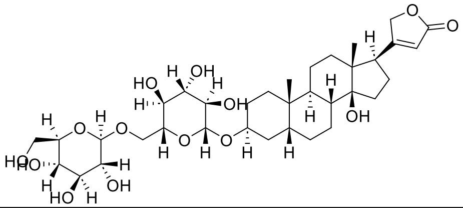
TKS80	Corchorusoside C	$C_{35}H_{54}O_{13}$	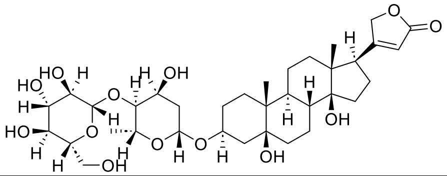

TKS81	Biondianoside A	$C_{42}H_{66}O_{18}$	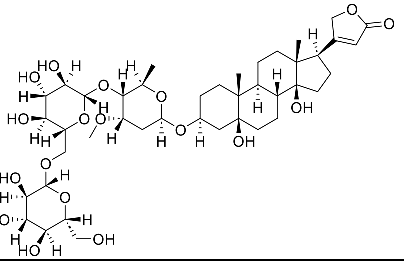
TKS82	17β-H-periplogenin 3-O-β-D-digitoxoside	$C_{29}H_{44}O_{8}$	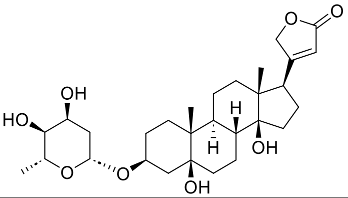
TKS83	△5-pregnene-3β,16α-diol-3-O-[2,4-O-diacetyl-β-digitalopyranosyl-(1→4)-β-D-cymaropyranoside]-16-O-[β-D-glucopyranoside]	$C_{45}H_{70}O_{17}$	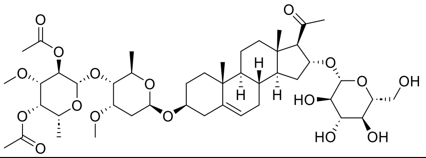
TKS84	Acovenosigenin A	$C_{23}H_{34}O_{5}$	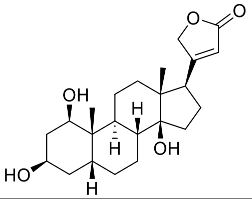
TKS85	Δ5-pregnene-3β,17α,20(S)-triol	$C_{21}H_{34}O_{3}$	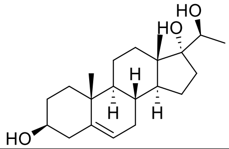
TKS86	Pinoresinol	$C_{20}H_{22}O_{6}$	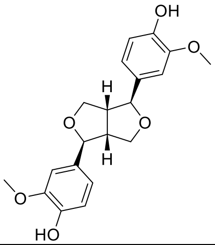
TKS87	Cleomiscosin A	$C_{20}H_{18}O_{8}$	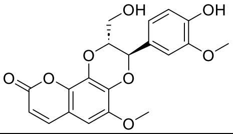
TKS88	Chinensin	$C_{21}H_{16}O_{6}$	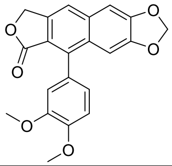
TKS89	Patriscabratine	$C_{27}H_{28}N_{2}O_{4}$	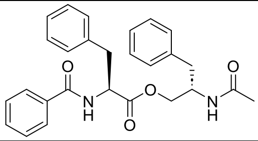

TKS90	(24R)-cycloartane-3β, 24, 25-triol	$C_{30}H_{52}O_3$	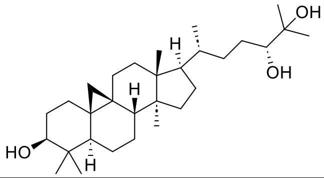
Compounds from SZM			
NO	Compound Name	Formula	Chemical structure
SZM1	helicterilic acid	$C_{39}H_{54}O_6$	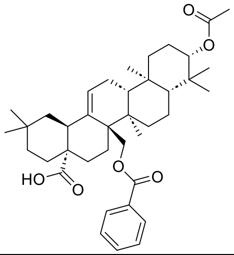
SZM2	Methyl helicterilate	$C_{40}H_{56}O_6$	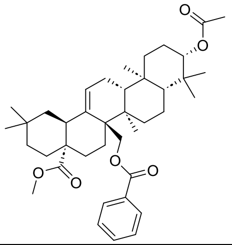
SZM3	Helicteric acid	$C_{39}H_{54}O_6$	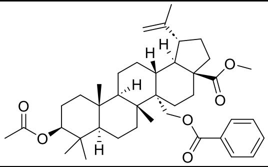
SZM4	methyl helicter	$C_{40}H_{56}O_6$	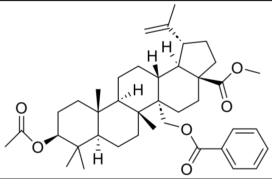
SZM5	Oleanic acid	$C_{30}H_{48}O_3$	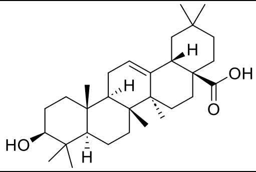
SZM6	Betulinic acid	$C_{30}H_{48}O_3$	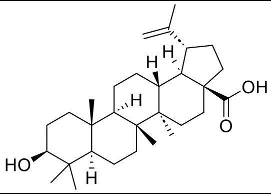
SZM7	Quinic acid	$C_7H_{12}O_6$	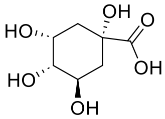
SZM8	Neochlorogenic acid	$C_{16}H_{18}O_9$	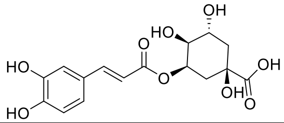

SZM9	Chlorogenic acid	$C_{16}H_{18}O_9$	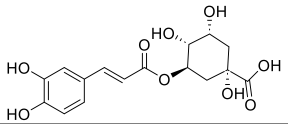
SZM10	Cryptochlorogenic acid	$C_{16}H_{18}O_9$	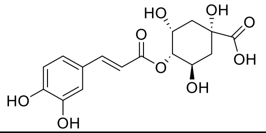
SZM11	Isochlorogenic acid C	$C_{25}H_{24}O_{12}$	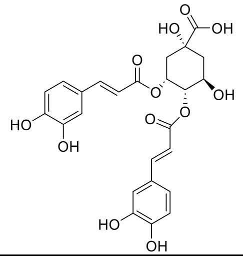
SZM12	Isochlorogenic acid A	$C_{25}H_{24}O_{12}$	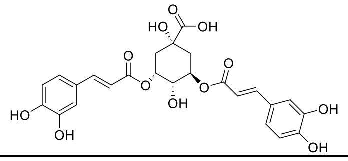
SZM13	Isochlorogenic acid B	$C_{25}H_{24}O_{12}$	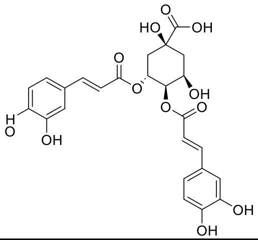
SZM14	cucurbitacin B 2-sulfate	$C_{32}H_{46}O_{11}S$	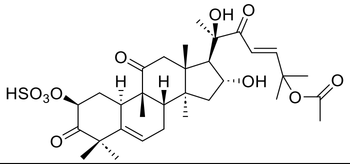
SZM15	cucurbitacin B	$C_{32}H_{46}O_8$	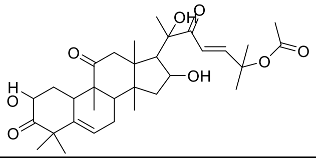
SZM16	cucurbitacin E	$C_{30}H_{40}O_8$	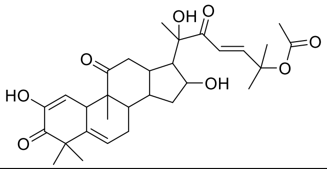
SZM17	rosmarinic acid	$C_{18}H_{16}O_8$	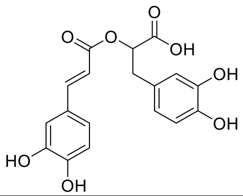
SZM18	Dodecanethiol	$C_{12}H_{26}S$	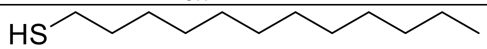
SZM19	β－sitosterol	$C_{29}H_{52}O$	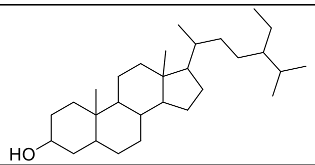

SZM20	Ursolic acid	$C_{29}H_{46}O_3$	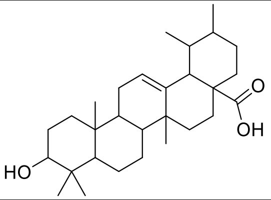
SZM21	Asarinin	$C_{20}H_{18}O_6$	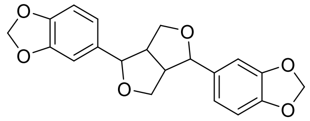
SZM22	Ergosterol	$C_{32}H_{54}O$	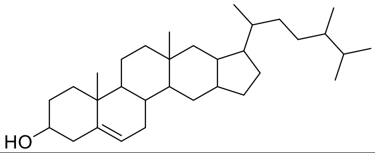
SZM23	2,6-Dimethoxyp-quinone	$C_8H_8O_4$	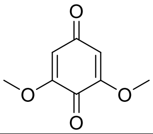
SZM24	3-acetoxybetulin	$C_{32}H_{52}O_3$	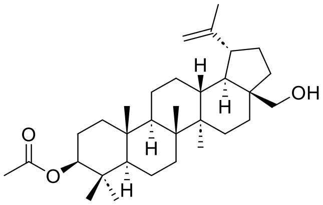
SZM25	3β-acetoxy-27-<br>(p-hydroxyl) benzoyloxylup-20(29)-en-28-<br>oic acid methyl ester	$C_{40}H_{56}O_7$	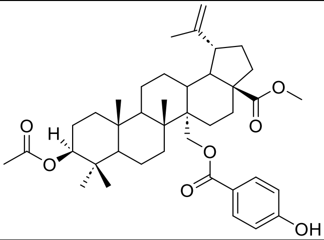
SZM26	3β-acetoxy-27-benzoyloxylup-20<br>(29)-en-28-oic acid	$C_{39}H_{54}O_6$	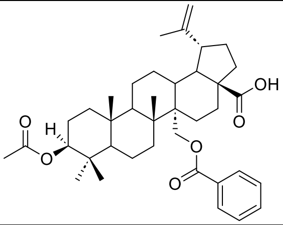
SZM27	3β-acetoxybetulinic<br>acid	$C_{32}H_{50}O_4$	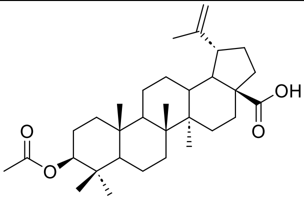
SZM28	pyracrenic acid	$C_{39}H_{54}O_6$	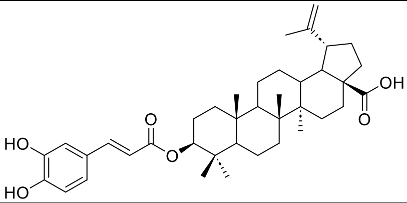

SZM29	cucurbitacin D	$C_{30}H_{44}O_7$	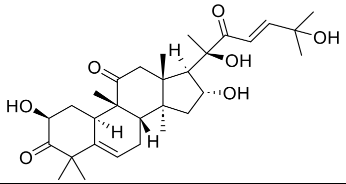
SZM30	isocucurbitacin D	$C_{30}H_{44}O_7$	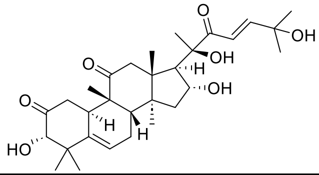
SZM31	3-acetoxy-27-［(4-hydroxybenzoyl) oxy］olean-12-en-28-oic acid methyl ester	$C_{39}H_{54}O_7$	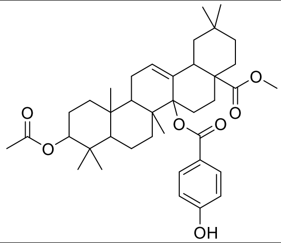
SZM32	2α,7β,20αtrihydroxy-3β,21-dimethoxy-5-pregnene	$C_{23}H_{38}O_5$	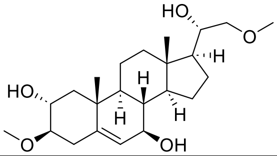
SZM33	hexadecanoic acid	$C_{16}H_{32}O_2$	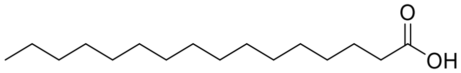
SZM34	daucosterol	$C_{35}H_{60}O_6$	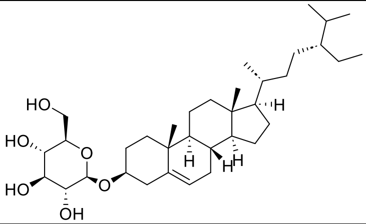
SZM35	heliclactone	$C_{15}H_{16}O_4$	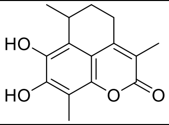
SZM36	Tricin	$C_{17}H_{14}O_7$	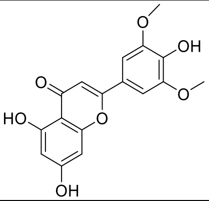
SZM37	3-O-[β-D-glucopyranose]glutathione-5-ene-3β-Alcohol glycoside	$C_{35}H_{60}O_6$	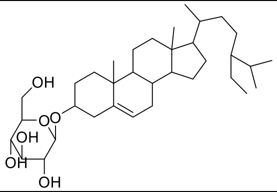
SZM38	2-O-b-D-glucopyranoside	$C_{36}H_{56}O_{13}$	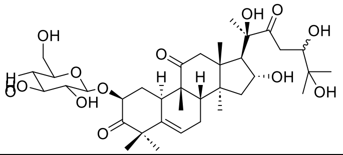
Compounds from GM			
No.	Name	Formula	Chemical structure

GM1	GM1	$C_{30}H_{48}O_4$	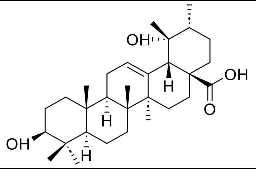
GM2	GM2	$C_{35}H_{56}O_8$	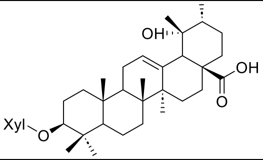
GM3	GM3	$C_{35}H_{56}O_8$	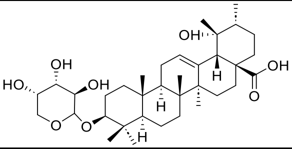
GM4	GM4	$C_{30}H_{48}O_7S$	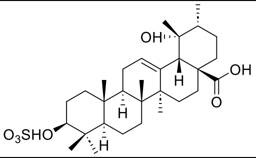
GM5	GM5	$C_{37}H_{58}O_9$	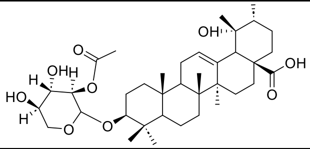
GM6	GM6	$C_{36}H_{56}O_{10}$	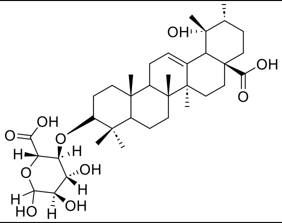
GM7	GM7	$C_{46}H_{74}O_{11}$	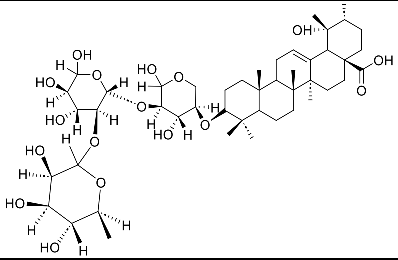
GM8	GM8	$C_{41}H_{66}O_{13}$	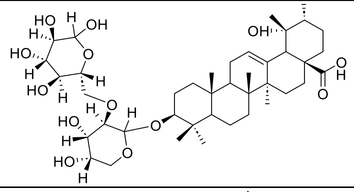
GM9	GM9	$C_{30}H_{48}O_4$	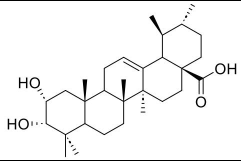
GM10	GM10	$C_{41}H_{66}O_{13}$	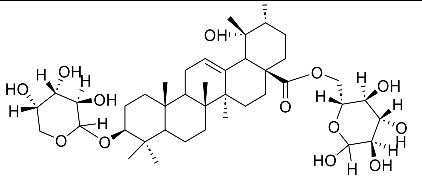

GM11	GM11	$C_{36}H_{58}O_9$	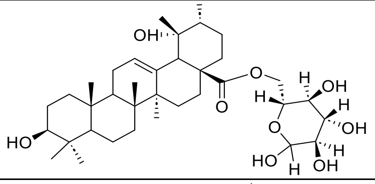
GM12	GM12	$C_{41}H_{66}O_{13}$	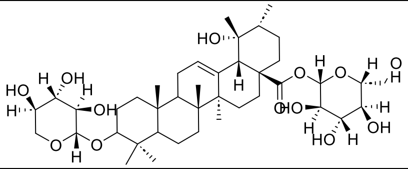
GM13	GM13	$C_{36}H_{57}NaO_{12}S$	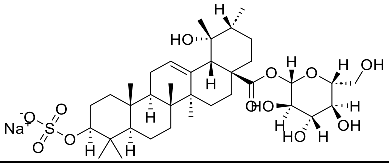
GM14	GM14	$C_{36}H_{58}O_{10}$	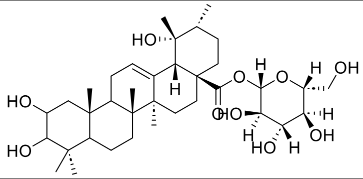
GM15	GM15	$C_{40}H_{64}O_{10}$	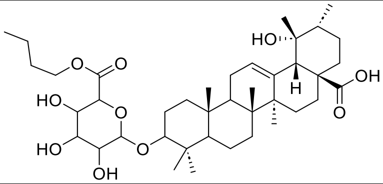
GM16	GM16	$C_{37}H_{58}O_{10}$	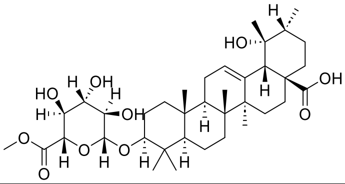
GM17	GM17	$C_{37}H_{57}NaO_{13}S$	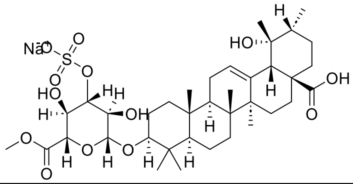
GM18	GM18	$C_{43}H_{68}O_{15}$	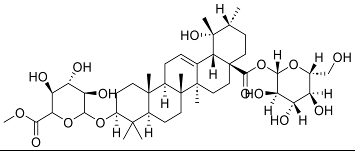
GM19	GM19	$C_{43}H_{67}NaO_{18}S$	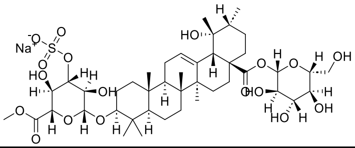
GM20	GM20	$C_{35}H_{54}O_7$	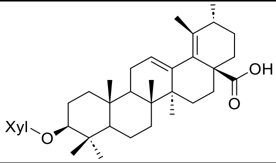

GM21	GM21	$C_{30}H_{46}O_3$	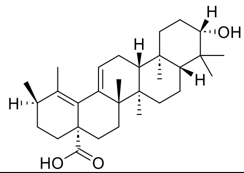
GM22	GM22	$C_{35}H_{54}O_7$	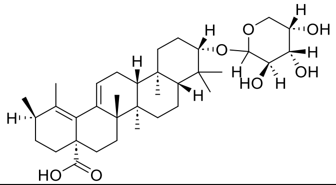
GM23	GM23	$C_{41}H_{64}O_{12}$	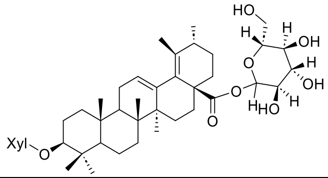
GM24	GM24	$C_{35}H_{54}O_{10}S$	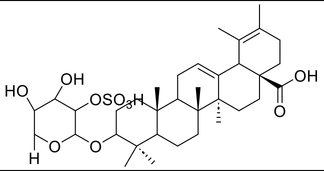
GM25	GM25	$C_{41}H_{64}O_{15}S$	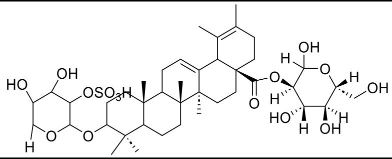
GM26	GM26	$C_{43}H_{66}O_{14}$	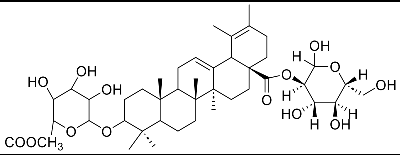
GM27	GM27	$C_{41}H_{66}O_{13}$	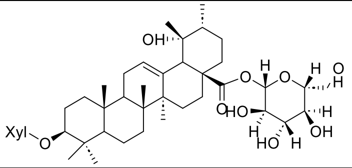
GM28	GM28	$C_{41}H_{66}O_{13}$	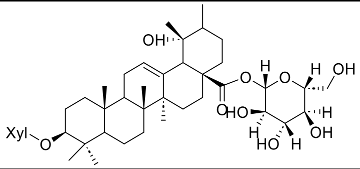
GM29	GM29	$C_{30}H_{46}O_3$	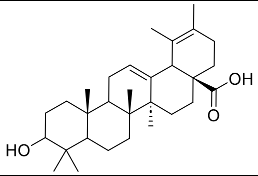
GM30	GM30	$C_{41}H_{64}O_{12}$	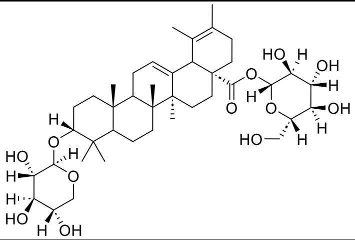

GM31	GM31	$C_{32}H_{50}O_4$	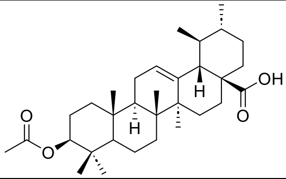
GM32	GM32	$C_{30}H_{48}O_3$	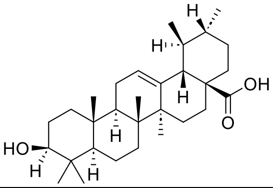
GM33	GM33	$C_{41}H_{66}O_{16}S$	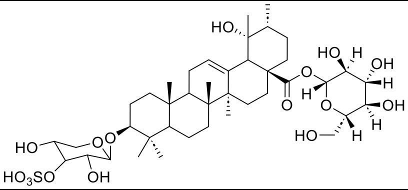
GM34	GM34	$C_{35}H_{56}O_{11}S$	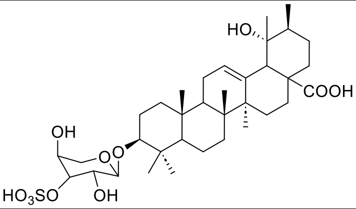
GM35	GM35	$C_{43}H_{66}O_{14}$	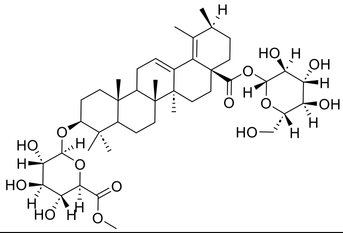
GM36	GM36	$C_{43}H_{66}O_{14}$	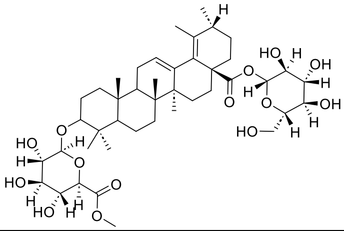
GM37	GM37	$C_{43}H_{66}O_{14}$	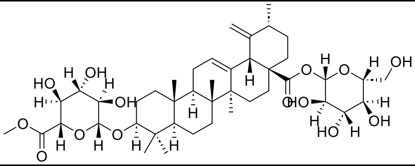
GM38	GM38	$C_{41}H_{64}O_{15}S$	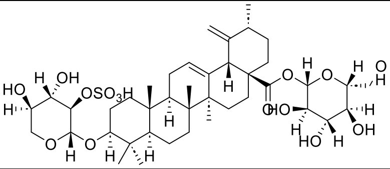
GM39	GM39	$C_{29}H_{46}O$	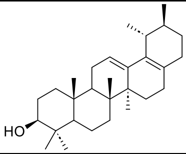

GM40	GM40	$C_{34}H_{54}O_5$	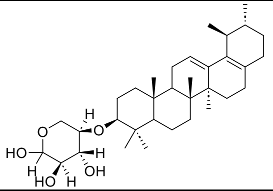
GM41	GM41	$C_{32}H_{52}O_3$	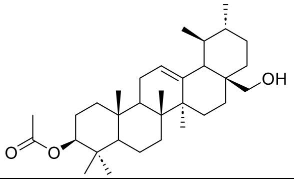
GM42	GM42	$C_{36}H_{56}O_{12}$	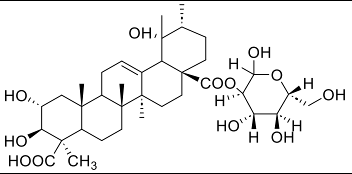
GM43	GM43	$C_{30}H_{46}O_7$	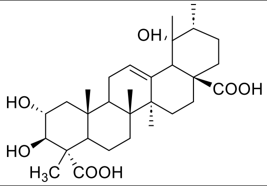
GM44	GM44	$C_{36}H_{56}O_{12}$	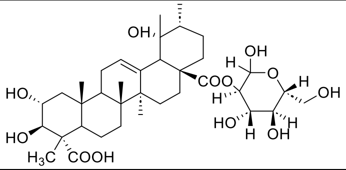
GM45	GM45	$C_{41}H_{66}O_{14}$	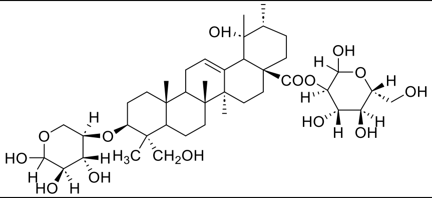
GM46	GM46	$C_{30}H_{46}O_6$	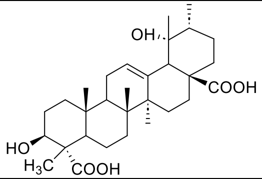
GM47	GM47	$C_{36}H_{56}O_{11}$	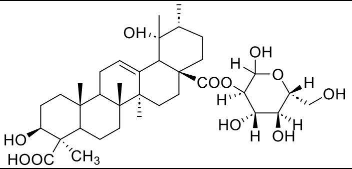
GM48	GM48	$C_{30}H_{46}O_6$	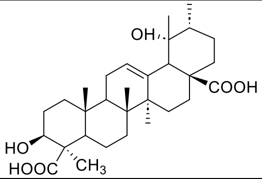

GM49	GM49	$C_{35}H_{54}O_8$	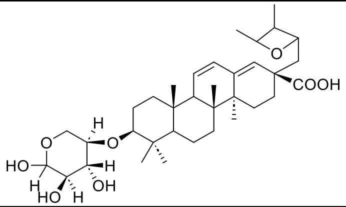
GM50	GM50	$C_{32}H_{52}O_3$	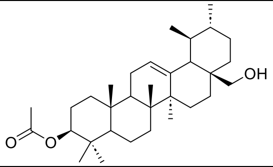
GM51	GM51	$C_{36}H_{56}O_9$	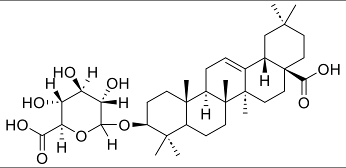
GM52	GM52	$C_{30}H_{48}O_3$	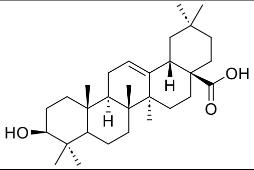
GM53	GM53	$C_{30}H_{46}O_7$	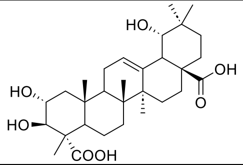
GM54	GM54	$C_{48}H_{60}O_8$	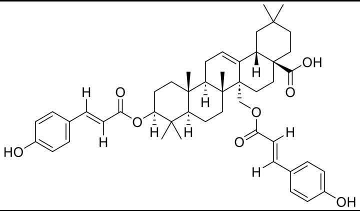
GM55	GM55	$C_{48}H_{60}O_8$	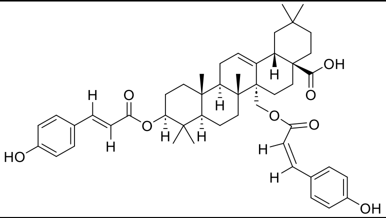
GM56	GM56	$C_{48}H_{60}O_8$	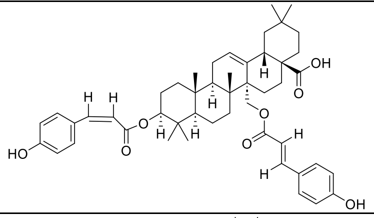
GM57	GM57	$C_{30}H_{48}O_4$	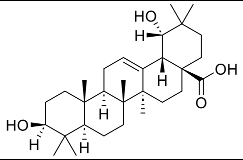
GM58	GM58	$C_{37}H_{58}O_9$	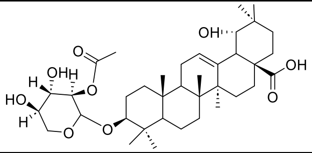

GM59	GM59	$C_{36}H_{56}O_{10}$	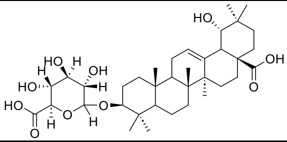
GM60	GM60	$C_{41}H_{66}O_{13}$	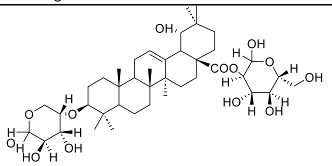
GM61	GM61	$C_{41}H_{66}O_{19}S_2$	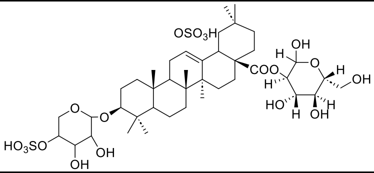
GM62	GM62	$C_{36}H_{56}O_{11}$	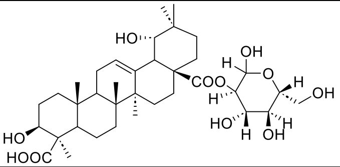
GM63	GM63	$C_{36}H_{56}O_{11}$,	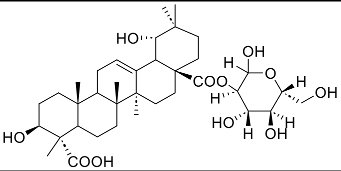
GM64	GM64	$C_{29}H_{38}O_7$	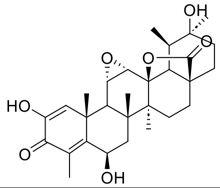
GM65	GM65	$C_{29}H_{36}O_6$	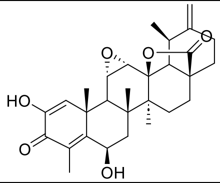
GM66	GM66	$C_{29}H_{38}O_6$	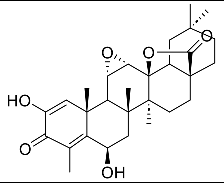
GM67	GM67	$C_{29}H_{38}O_6$	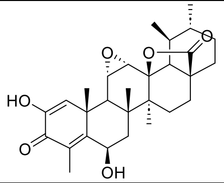
GM68	GM68	$C_{16}H_{18}O_9$	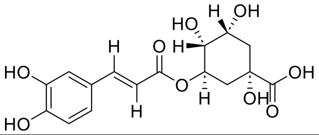

GM69	GM69	$C_{16}H_{18}O_{9}$	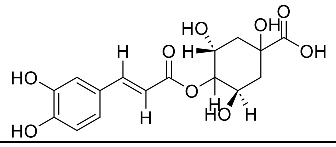
GM70	GM70	$C_{26}H_{26}O_{12}$	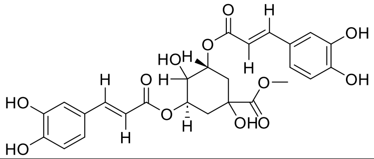
GM71	GM71	$C_{23}H_{22}O_{12}$	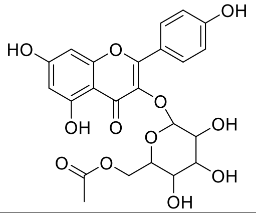
GM72	GM72	$C_{21}H_{20}O_{11}$	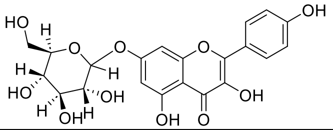
GM73	GM73	$C_{16}H_{12}O_{6}$	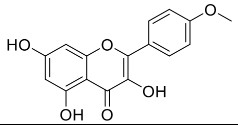
GM74	GM74	$C_{21}H_{20}O_{11}$	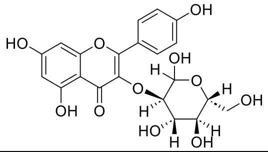
GM75	GM75	$C_{27}H_{30}O_{16}$	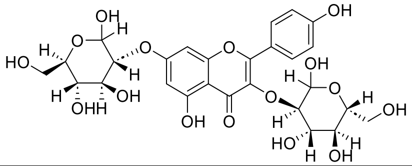
GM76	GM76	$C_{15}H_{10}O_{6}$	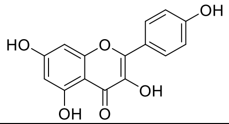
GM77	GM77	$C_{15}H_{10}O_{7}$	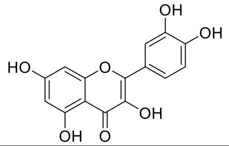
GM78	GM78	$C_{21}H_{20}O_{12}$	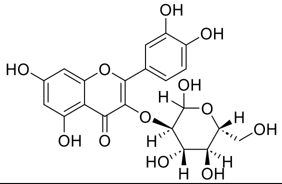
GM79	GM79	$C_{27}H_{30}O_{16}$	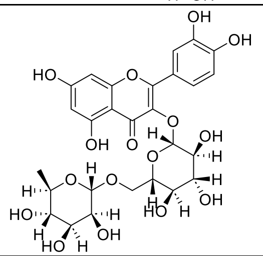

GM80	GM80	$C_9H_8O_4$	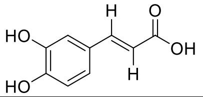
GM81	GM81	$C_{10}H_{10}O_4$	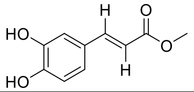
GM82	GM82	$C_{10}H_{10}O_3$	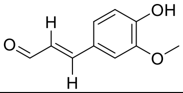
GM83	GM83	$C_{29}H_{36}O_{16}$	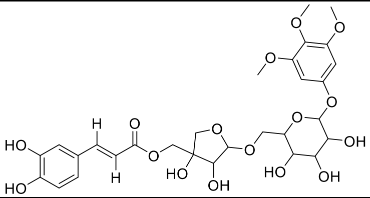
GM84	GM84	$C_{26}H_{34}O_{11}$	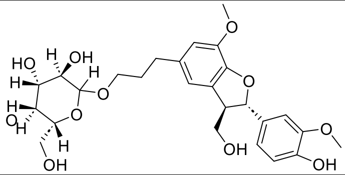
GM85	GM85	$C_{16}H_{22}O_8$	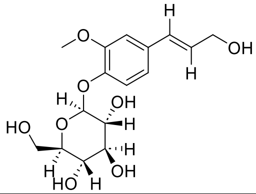
GM86	GM86	$C_{22}H_{26}O_8$	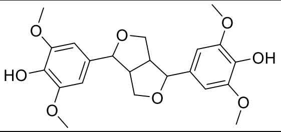
GM87	GM87	$C_{28}H_{36}O_{13}$	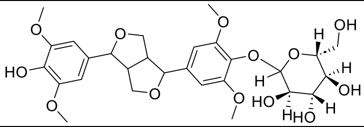
GM88	GM88	$C_{26}H_{32}O_{12}$	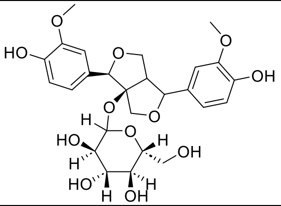
GM89	GM89	$C_{27}H_{34}O_{13}$	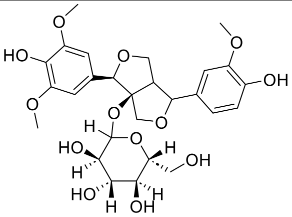
GM90	GM90	$C_{21}H_{24}O_7$	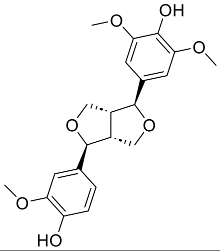

GM91	GM91	$C_9H_{10}O_5$	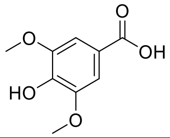
GM92	GM92	$C_9H_{10}O_4$	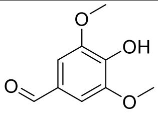
GM93	GM93	$C_7H_6O_4$	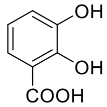
GM94	GM94	$C_7H_6O_4$	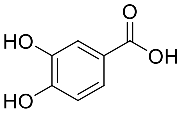
GM95	GM95	$C_{11}H_{14}O_5$	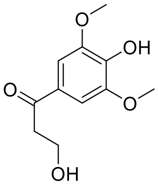
GM96	GM96	$C_{10}H_{12}O_4$	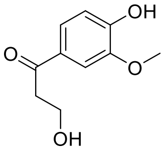
GM97	GM97	$C_{51}H_{88}O_7$	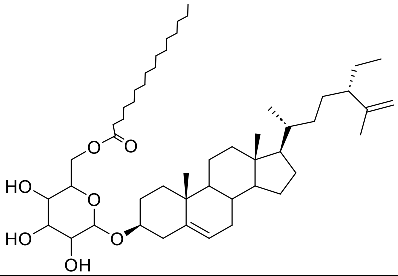
GM98	GM98	$C_{53}H_{92}O_7$	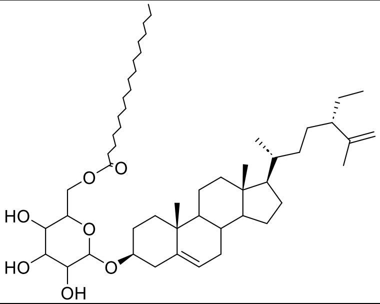
GM99	GM99	$C_{29}H_{48}O$	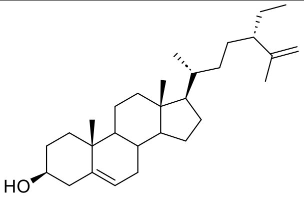
GM100	GM100	$C_{35}H_{58}O_6$	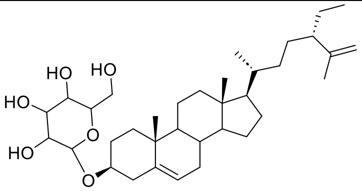

GM101	GM101	$C_{35}H_{60}O_6$	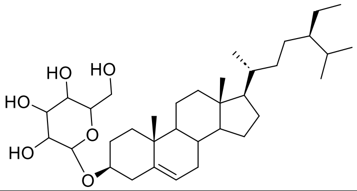
GM102	GM102	$C_{19}H_{28}O_{10}$	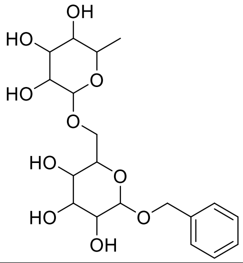
GM103	GM103	$C_{16}H_{28}O_7$	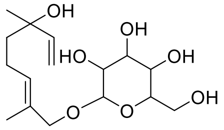
GM104	GM104	$C_{20}H_{24}O_7$	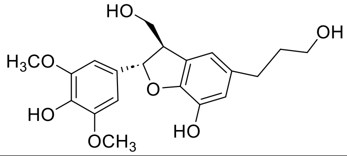
GM105	GM105	$C_{27}H_{36}O_{12}$	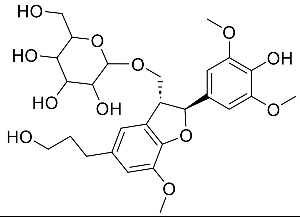
GM106	GM106	$C_{20}H_{24}O_7$	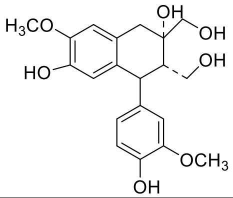
GM107	GM107	$C_{28}H_{36}O_{13}$	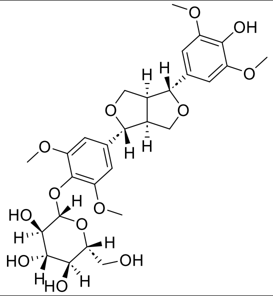

GM108	GM108	$C_{34}H_{46}O_{18}$	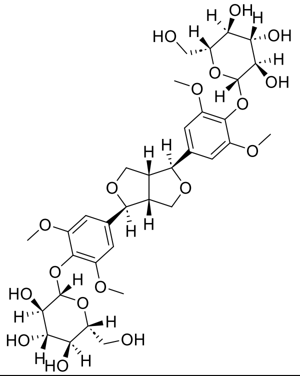
GM109	GM109	$C_8H_8O_4$	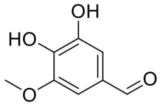
GM110	GM110	$C_{20}H_{30}O_{13}$	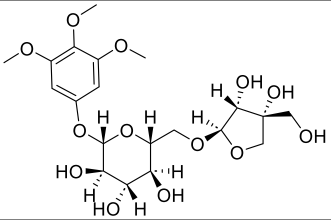
GM111	GM111	$C_9H_6O_4$	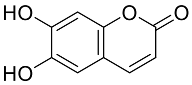
GM112	GM112	$C_{18}H_{22}O_9$	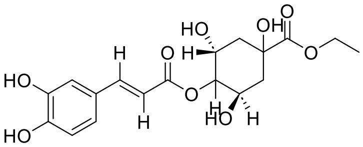
GM113	GM113	$C_{18}H_{22}O_9$	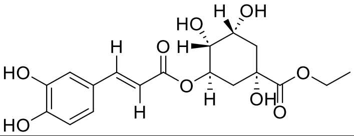
GM114	GM114	$C_{15}H_{20}O_6$	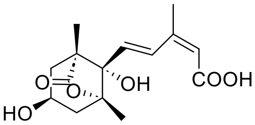
GM115	GM115	$C_{42}H_{66}O_{17}S$	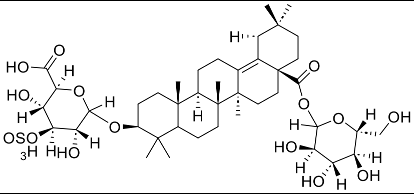
GM116	GM116	$C_{47}H_{76}O_{18}$	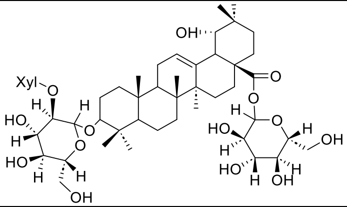
GM117	GM117	$C_{41}H_{66}O_{14}$	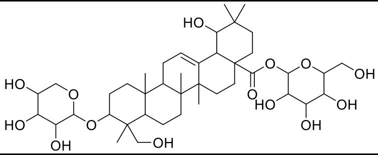

GM118	GM118	$C_{47}H_{76}O_{18}$	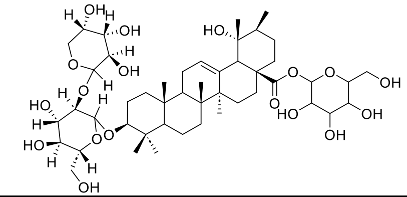
GM119	GM119	$C_{47}H_{76}O_{17}$	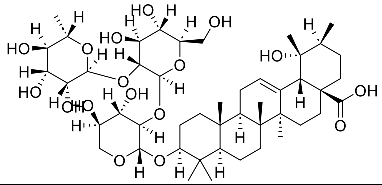
GM120	GM120	$C_{47}H_{76}O_{18}$	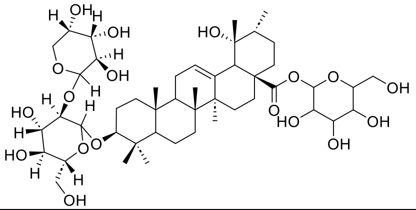
GM121	GM121	$C_{47}H_{76}O_{18}$	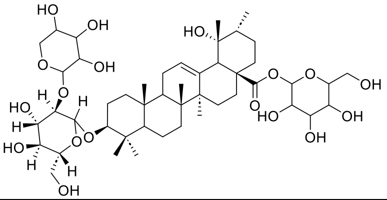
GM122	GM122	$C_{42}H_{66}O_{15}$	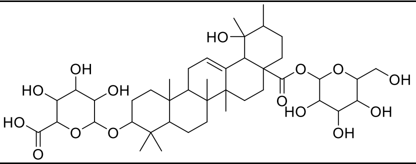
GM123	GM123	$C_{53}H_{86}O_{22}$	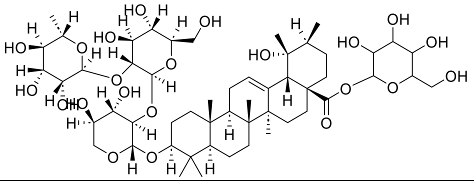
GM124	GM124	$C_{41}H_{66}O_{14}$	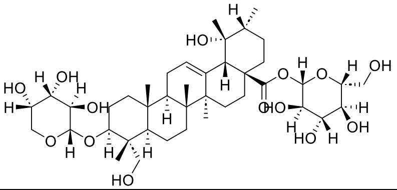
GM125	GM125	$C_{36}H_{56}O_{11}$	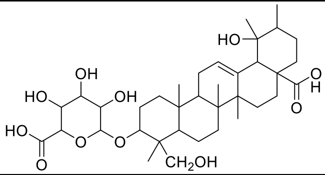
GM126	GM126	$C_{49}H_{78}O_{20}$	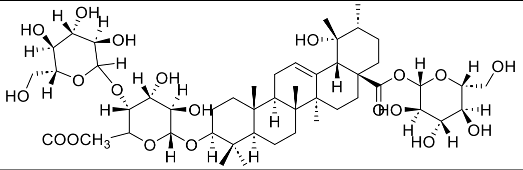

GM127	GM127	$C_{42}H_{66}O_{16}$	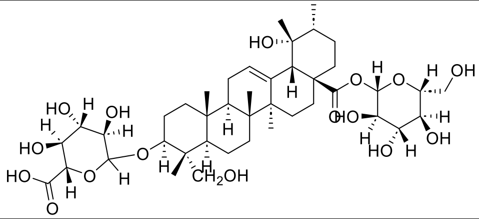
GM128	GM128	$C_{53}H_{86}O_{22}$	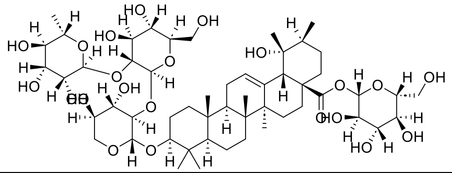
GM129	GM129	$C_{19}H_{22}O_{6}$	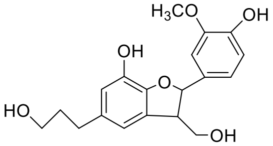
GM130	GM130	$C_{27}H_{38}O_{13}$	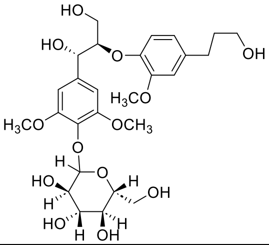
GM131	GM131	$C_{17}H_{26}O_{10}$	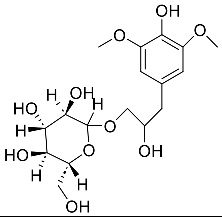
GM132	GM132	$C_{12}H_{18}O_{5}$	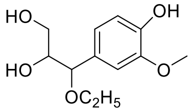
GM133	GM133	$C_{13}H_{20}O_{6}$	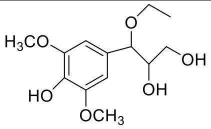
GM134	GM134	$C_{11}H_{14}O_{6}$	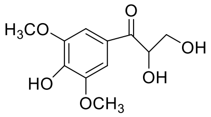
GM135	GM135	$C_{15}H_{18}O_{8}$	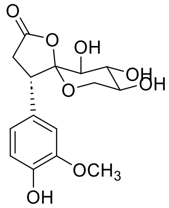

GM136	GM136	$C_{26}H_{26}O_{12}$	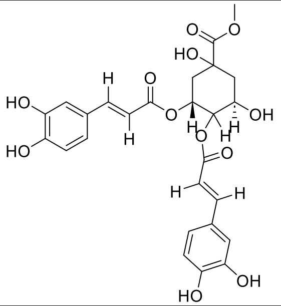
GM137	GM137	$C_{27}H_{28}O_{12}$	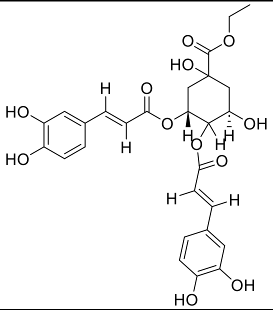
GM138	GM138	$C_{26}H_{26}O_{12}$	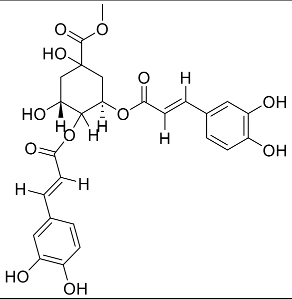
GM139	GM139	$C_{27}H_{28}O_{12}$	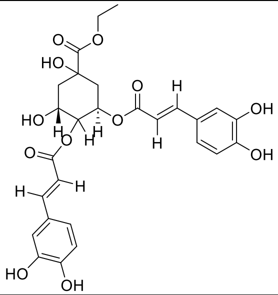
GM140	GM140	$C_{26}H_{26}O_{12}$	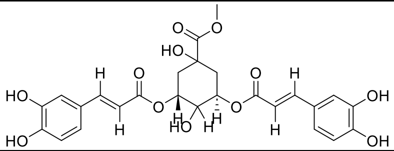
GM141	GM141	$C_{27}H_{28}O_{12}$	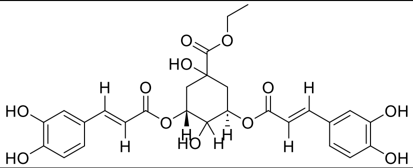
GM142	GM142	$C_{15}H_{20}O_{9}$	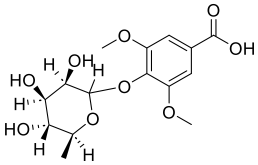
GM143	GM143	$C_{8}H_{8}O_{4}$	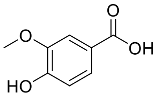

GM144	GM144	$C_8H_8O_5$	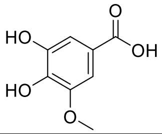
GM145	GM145	$C_9H_{10}O_4$	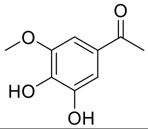
GM146	GM146	$C_{36}H_{56}O_{13}S$	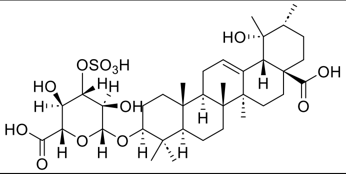
GM147	GM147	$C_{42}H_{66}O_{18}S$	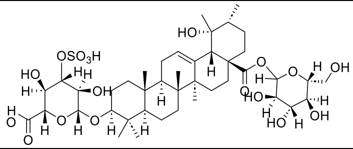
GM148	GM148	$C_{35}H_{56}O_{11}S$	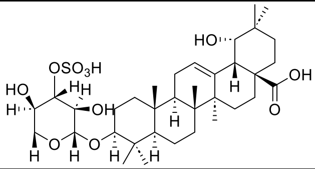
GM149	GM149	$C_{41}H_{66}O_{16}S$	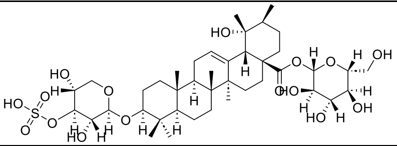
GM150	GM150	$C_{41}H_{66}O_{19}S_2$	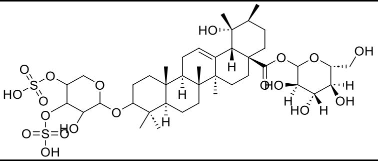
GM151	GM151	$C_{41}H_{66}O_{19}S_2$	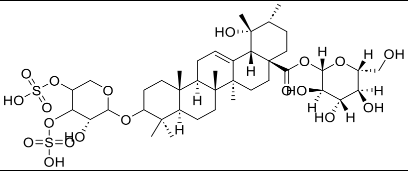
GM152	GM152	$C_{47}H_{74}O_{17}$	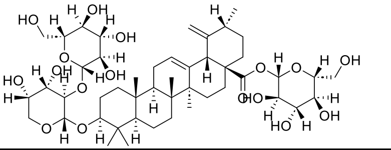
GM153	GM153	$C_{47}H_{74}O_{17}$	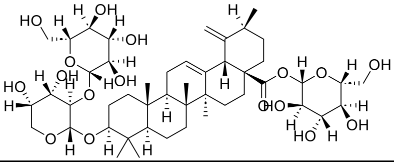
GM154	GM154	$C_{42}H_{64}O_{17}S$	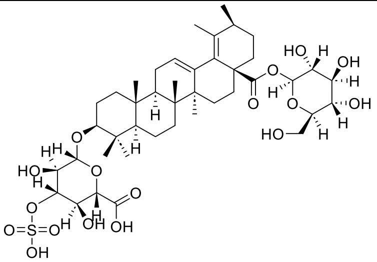

GM155	GM155	$C_{47}H_{74}O_{17}$	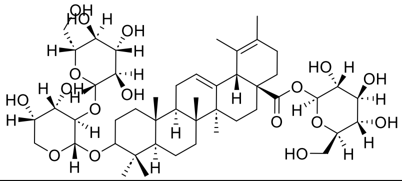
GM156	GM156	$C_{41}H_{66}O_{15}S$	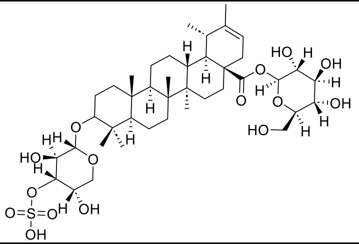
GM157	GM157	$C_{47}H_{76}O_{17}$	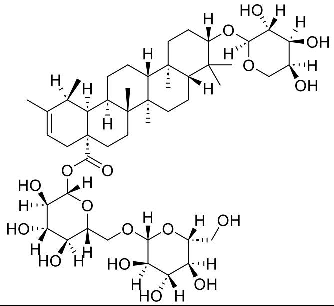
GM158	GM158	$C_{36}H_{56}O_{13}S$	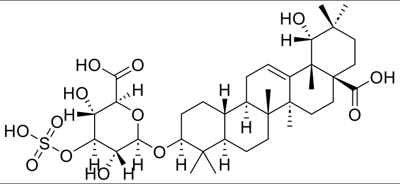
GM159	GM159	$C_{47}H_{76}O_{17}$	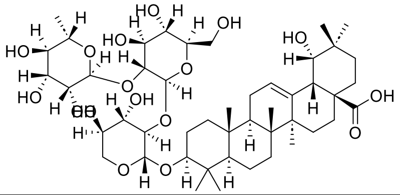
GM160	GM160	$C_{22}H_{30}O_{12}$	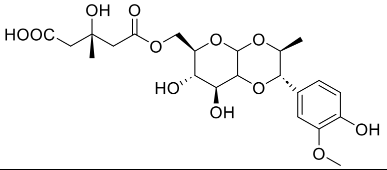
GM161	GM161	$C_{22}H_{28}O_{9}$	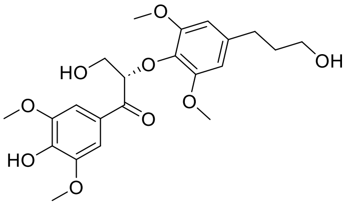
GM162	GM162	$C_{20}H_{24}O_{10}$	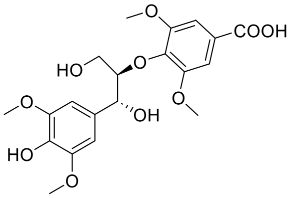

GM163	GM163	$C_{20}H_{24}O_{10}$	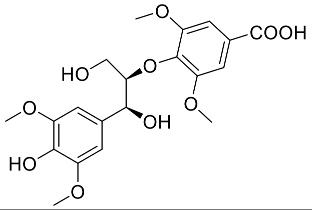
GM164	GM164	$C_{20}H_{24}O_{8}$	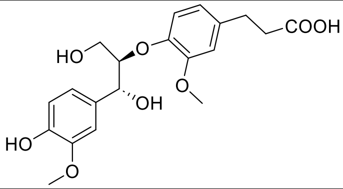
GM165	GM165	$C_{20}H_{24}O_{8}$	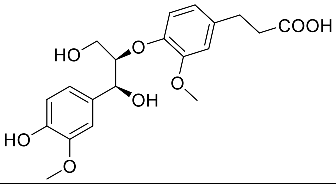
GM166	GM166	$C_{19}H_{22}O_{9}$	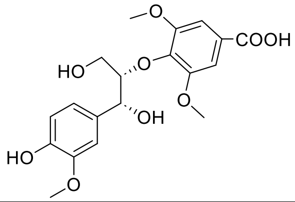
GM167	GM167	$C_{21}H_{28}O_{8}$	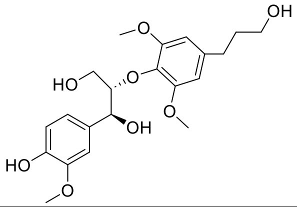
GM168	GM168	$C_{47}H_{76}O_{17}$	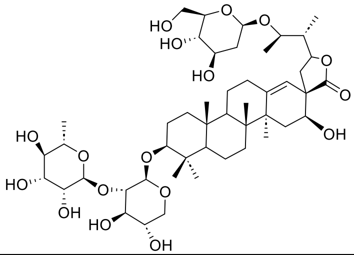
GM169	GM169	$C_{47}H_{74}O_{17}$	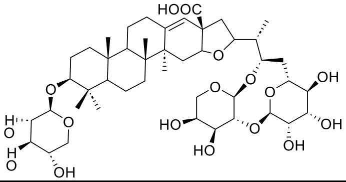
GM170	GM170	$C_{28}H_{34}O_{15}$	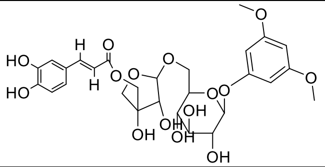
GM171	GM171	$C_{36}H_{56}O_{12}$	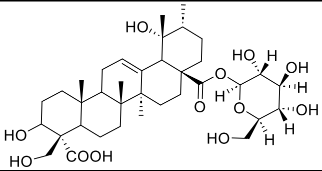

GM172	GM172	$C_{41}H_{66}O_{19}S_2$	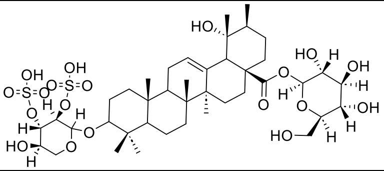
GM173	GM173	$C_{47}H_{76}O_{17}$	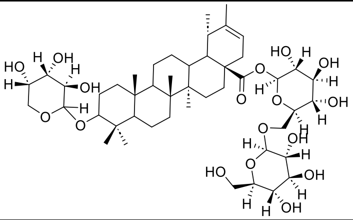
GM174	GM174	$C_{36}H_{56}O_{11}$	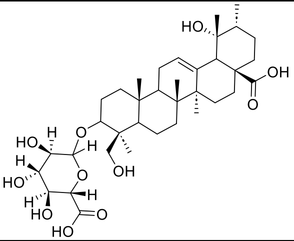
GM175	GM175	$C_{41}H_{66}O_{16}S$	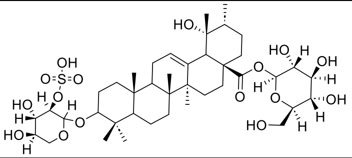
GM176	GM176	$C_{41}H_{58}O_8$	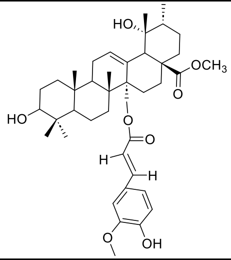
GM177	GM177	$C_{36}H_{56}O_{13}S$	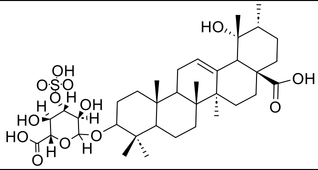
GM178	GM178	$C_{42}H_{66}O_{14}$	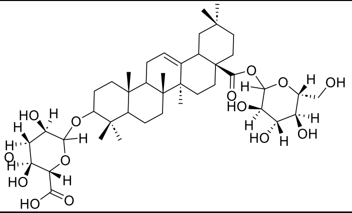
GM179	GM179	$C_{40}H_{56}O_6$	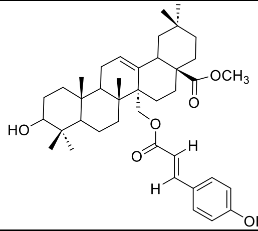

GM180	GM180	$C_{39}H_{54}O_7$	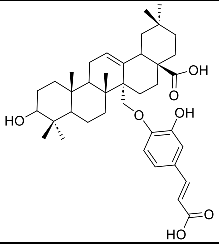
GM181	GM181	$C_{39}H_{54}O_6$	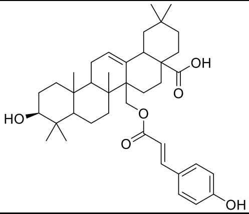
GM182	GM182	$C_{45}H_{64}O_{12}$	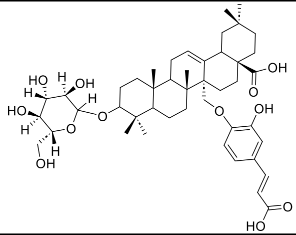
GM183	GM183	$C_{45}H_{64}O_{11}$	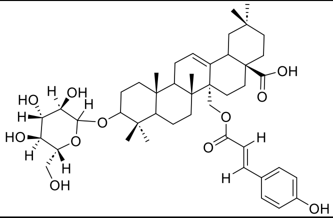
GM184	GM184	$C_{48}H_{60}O_8$	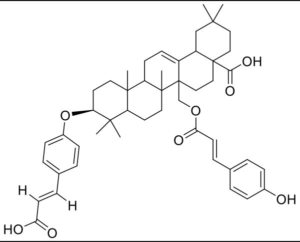
GM185	GM185	$C_{49}H_{62}O_9$	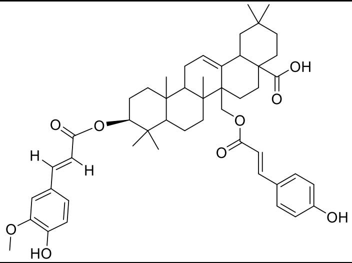
**Compounds from CEC**			
**NO.**	**Compound Name**	**Formula**	**Chemical structure**
CEC1	Caffeic acid	$C_9H_8O_4$	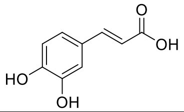
CEC2	1,3,5-tri-O-caffeoylquinic acid	$C_{34}H_{30}O_{15}$	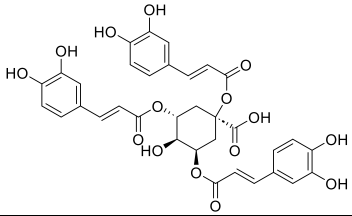

CEC3	1,5-di-O-caffeoylquinic acid	$C_{25}H_{24}O_{12}$	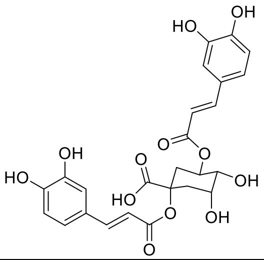
CEC4	3-O-caffeoylquinic acid(Chlorogenic acid)	$C_{16}H_{18}O_9$	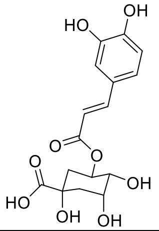
CEC5	5-O-caffeoylquinic acid(Neochlorogenic acid	$C_{16}H_{18}O_9$	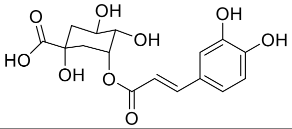
CEC6	1,4-di-O-caffeoylquinic acid	$C_{25}H_{24}O_{12}$	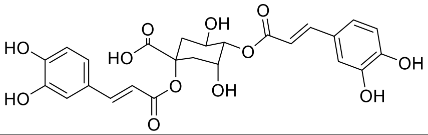
CEC7	4,5-di-O-caffeoylquinic ac-id(Isochlorogenic acid C)	$C_{25}H_{24}O_{12}$	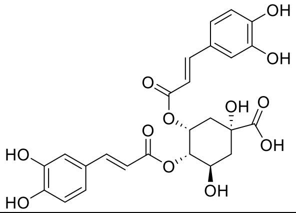
CEC8	1-O-caffeoylquinic acid	$C_{16}H_{18}O_9$	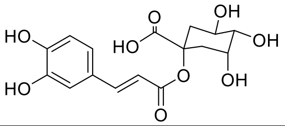
CEC9	4-O-caffeoylquinic acid(cryptochlorogenic acid)	$C_{16}H_{18}O_9$	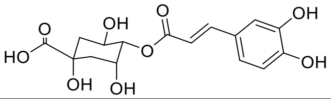
CEC10	Ferulic acid	$C_{10}H_{10}O_4$	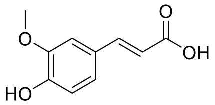
CEC11	Protocatechuate	$C_7H_6O_4$	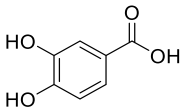
CEC12	Protocatechualdehyde	$C_7H_6O_3$	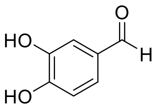
CEC13	3,5-di-O-caffeoylquinic ac-id(Isochlorogenic acid A)	$C_{25}H_{24}O_{12}$	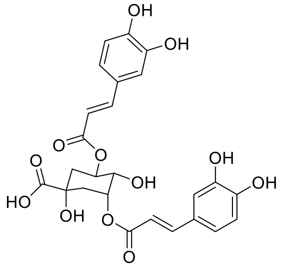

CEC14	4-caffeioyl quinine methyl ester	$C_{19}H_{26}O_7$	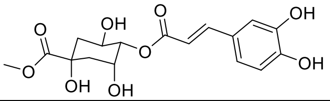
CEC15	3-caffeioyl quinine methyl ester(Chlorogenic acid methyl ester)	$C_{17}H_{20}O_9$	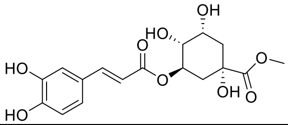
CEC16	3,4-di-O-caffeoylquinic acid(Isochlorogenic acid B)	$C_{25}H_{24}O_{12}$	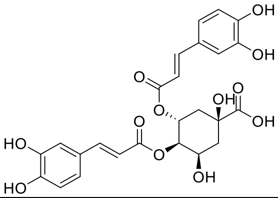
CEC17	5-O-caffeoyl quinic acid methyl ester	$C_{17}H_{20}O_9$	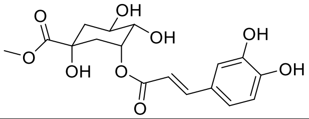
CEC18	Caffeic acid choline ester	$C_{14}H_{20}O_4N^+$	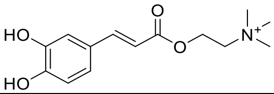
CEC19	11α,13 -dihydro -8 -epi -xanthatin	$C_{15}H_{20}O_3$	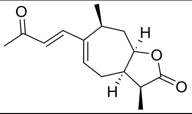
CEC20	xanthiazone	$C_{11}H_{13}NO_3S$	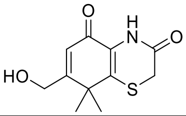
CEC21	Xanthatin	$C_{15}H_{18}O_3$	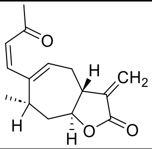
CEC22	Xanthinin	$C_{17}H_{22}O_5$	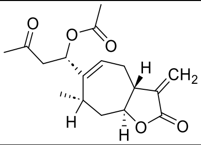
CEC23	Xanthumin	$C_{17}H_{22}O_5$	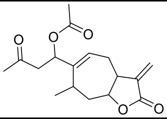
CEC24	Xanthanol	$C_{17}H_{24}O_5$	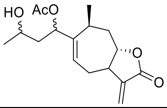
CEC25	Vanillin	$C_8H_8O_3$	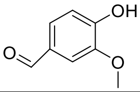
CEC26	11α,13 -dihydrotornentosin	$C_{15}H_{20}O_3$	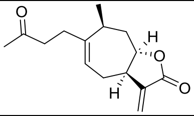
CEC27	8-Epixanthatin	$C_{15}H_{18}O_3$	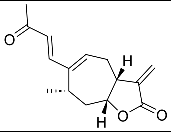

CEC28	Xanthodiene	$C_{15}H_{20}O_2$	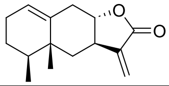
CEC29	Isoalantolactone	$C_{15}H_{20}O_2$	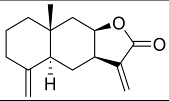
CEC30	Eudesmanolide	$C_{19}H_{26}O_2$	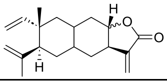
CEC31	eudesmanolide 8 -epimer	$C_{19}H_{26}O_2$	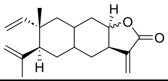
CEC32	2 -hydroxytomentosin	$C_{15}H_{20}O_4$	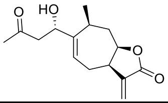
CEC33	Xanthumin	$C_{17}H_{22}O_5$	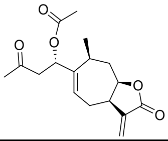
CEC34	2 -epi -xanthumin	$C_{17}H_{22}O_5$	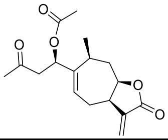
CEC35	8 -epi -xan -thatin -1β,5β -epoxide	$C_{15}H_{18}O_4$	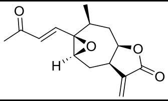
CEC36	tomentosin	$C_{15}H_{20}O_3$	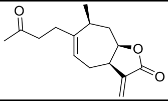
CEC37	1β,5β -epoxy -1,5 -dihydroxanthatin	$C_{15}H_{18}O_4$	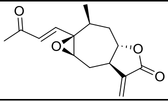
CEC38	1α,5α-epoxy-1,5 -dihydroxanthatin	$C_{15}H_{18}O_4$	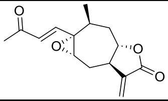
CEC39	2 -oxo -4 -O -acetyl -desacetylxanthanol	$C_{17}H_{22}O_5$	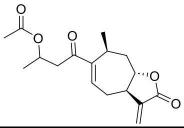
CEC40	4 -O -β -D -glucopyranosyl -11α,13 - dihydro -8 -epi -desaeetyllx -anthiuminol	$C_{21}H_{34}O_9$	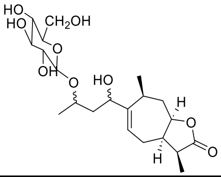
CEC41	2 -O -D -glucopyranosyl -11α,13 -di - hydro -8 -eDidesacetyllxanthiuminol	$C_{21}H_{34}O_9$	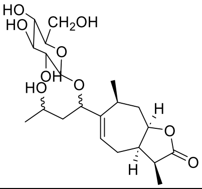
CEC42	1β,5β -epoxy -11α,13 -dihydrotomentosin	$C_{15}H_{20}O_4$	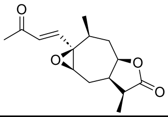

CEC43	3',4',5,7-Tetramethoxyflavone	$C_{19}H_{18}O_6$	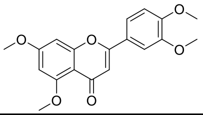
CEC44	Xanthipungolide	$C_{15}H_{18}O_3$	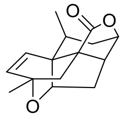
CEC45	pungiolide A	$C_{30}H_{36}O_7$	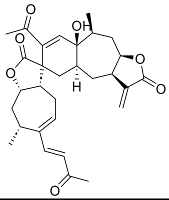
CEC46	pungiolide B	$C_{30}H_{36}O_8$	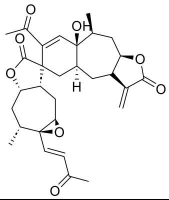
CEC47	1β,5β -epoxy -1,5,11α,13 -tetra -hydro -8 -epi -xanthatin	$C_{15}H_{20}O_4$	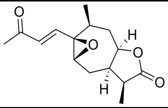
CEC48	1α,5α -epoxy -1,5,11α,13 -tetrahydro -8 -epi -xanthatin	$C_{15}H_{20}O_4$	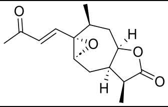
CEC49	sesquiterpene guaiamlide ziniolide	$C_{15}H_{18}O_2$	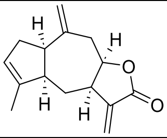
CEC50	emodin	$C_{15}H_{10}O_5$	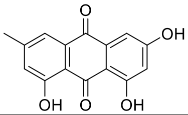
CEC51	Aloe emodin	$C_{15}H_{10}O_5$	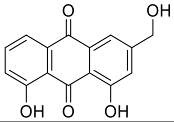
CEC52	chrysophanol	$C_{15}H_{10}O_4$	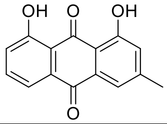
CEC53	quercetin	$C_{15}H_{10}O_7$	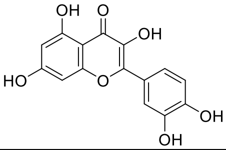
CEC54	Hexadecenoic acid	$C_{16}H_{30}O_2$	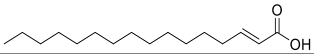
CEC55	Palmitic acid	$C_{16}H_{32}O_2$	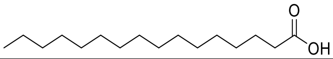
CEC56	stearic acid	$C_{18}H_{36}O_2$	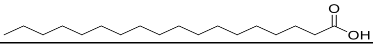
CEC57	Arachidic acid	$C_{20}H_{40}O_2$	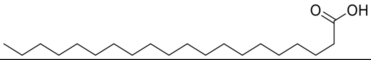
CEC58	Docosanoic acid	$C_{22}H_{44}O_2$	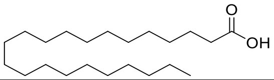

CEC59	2-decyltetradecanoic acid	$C_{24}H_{48}O_2$	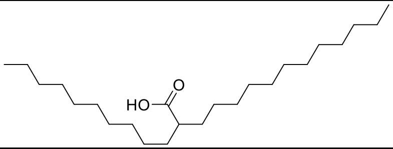
CEC60	oleic acid	$C_{18}H_{34}O_2$	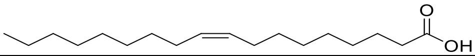
CEC61	Linoleic acid	$C_{18}H_{32}O_2$	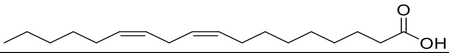
CEC62	Octanoic acid	$C_8H_{16}O_2$	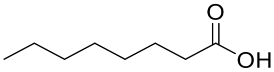
CEC63	Undecanoic acid	$C_{11}H_{22}O_2$	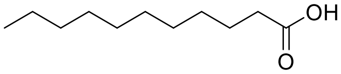
CEC64	Pentadecanoic acid	$C_{15}H_{30}O_2$	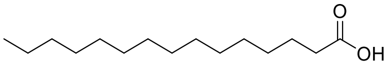
CEC65	Margaric acid	$C_{17}H_{34}O_2$	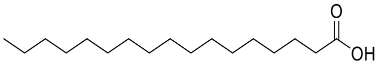
CEC66	Elaidic Acid	$C_{18}H_{34}O_2$	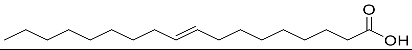
CEC67	Linolelaidic acid	$C_{18}H_{32}O_2$	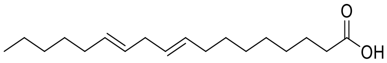
CEC68	4′-methyoxylisoflavone-7-O-β D-glucopyranoside	$C_{22}H_{22}O_9$	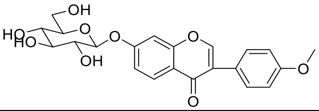
CEC69	Rutin	$C_{27}H_{30}O_{16}$	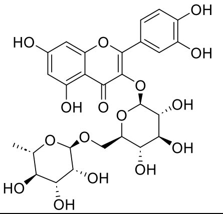
CEC70	Myristic acid	$C_{14}H_{28}O_2$	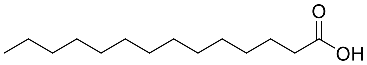
CEC71	sucrose	$C_{12}H_{22}O_{11}$	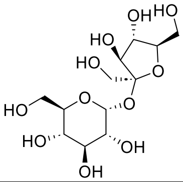

Detail MS, MS/MS information of the identified compounds of WGFSG

Retention Time: 0.99 minutes<br>Precursor m/z : 341.1089	**Exp RT:** 0.99 minutes<br>**Analyte Name:**<br>CEC71
	Collision Energy = 35 ± 15 eV

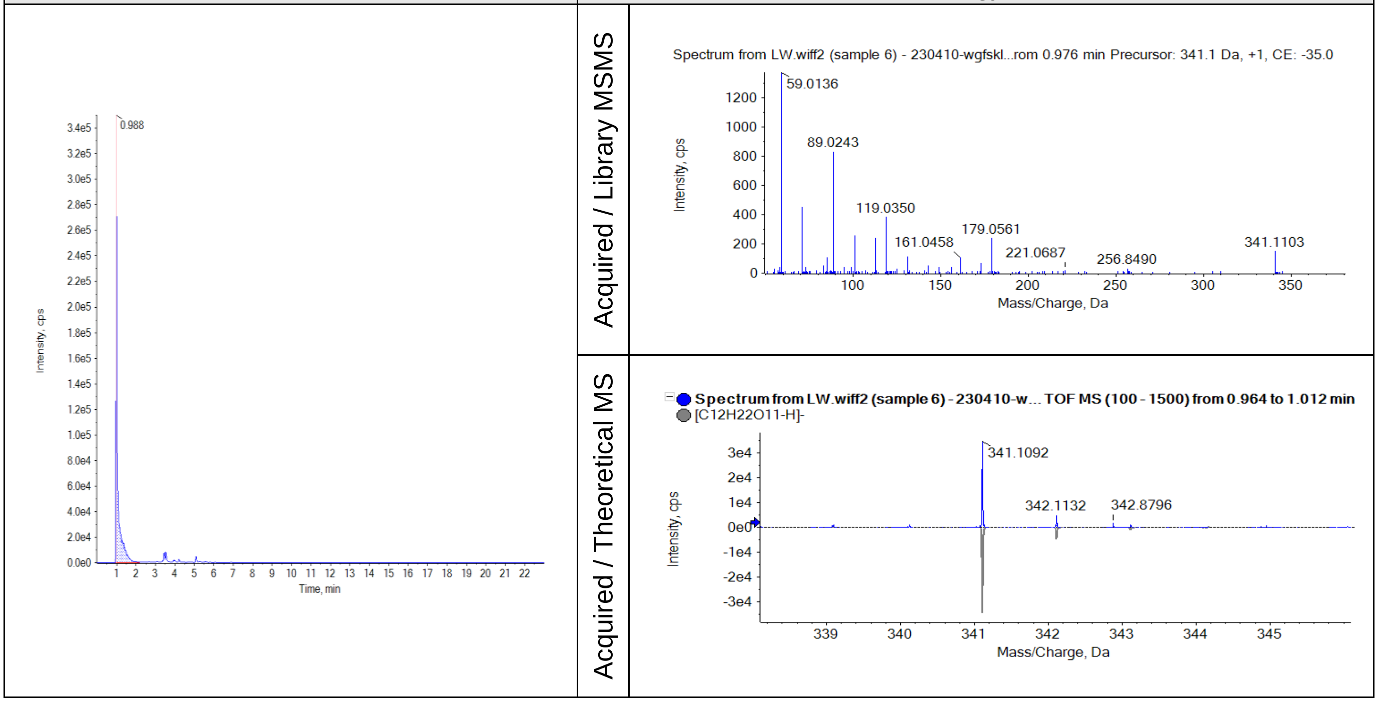

Fig. S1 Detail MS, MS/MS information of compound 1.

**Retention Time:** 1.04 minutes<br>**Precursor m/z :** 191.0561	**Exp RT:** 1.04 minutes<br>**Analyte Name:**<br>SZM7
	**Collision Energy = 35 ± 15 eV**

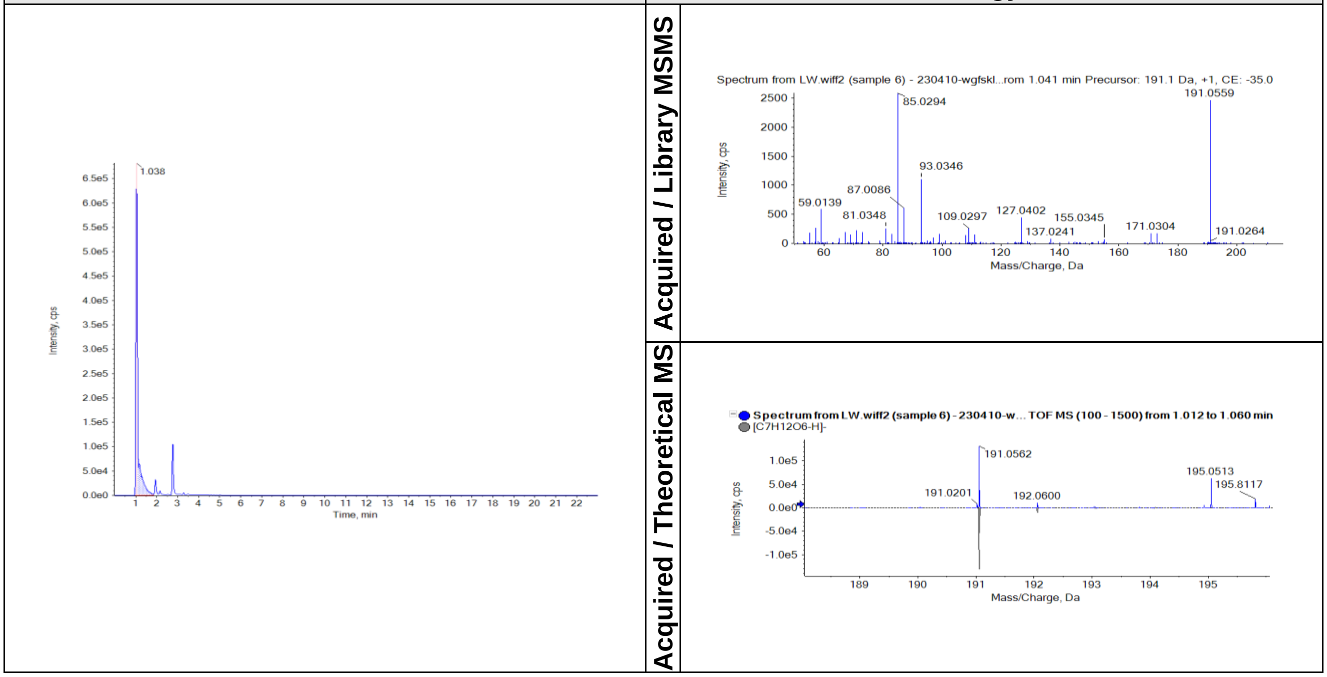

Fig. S2. Detail MS, MS/MS information of compound 2

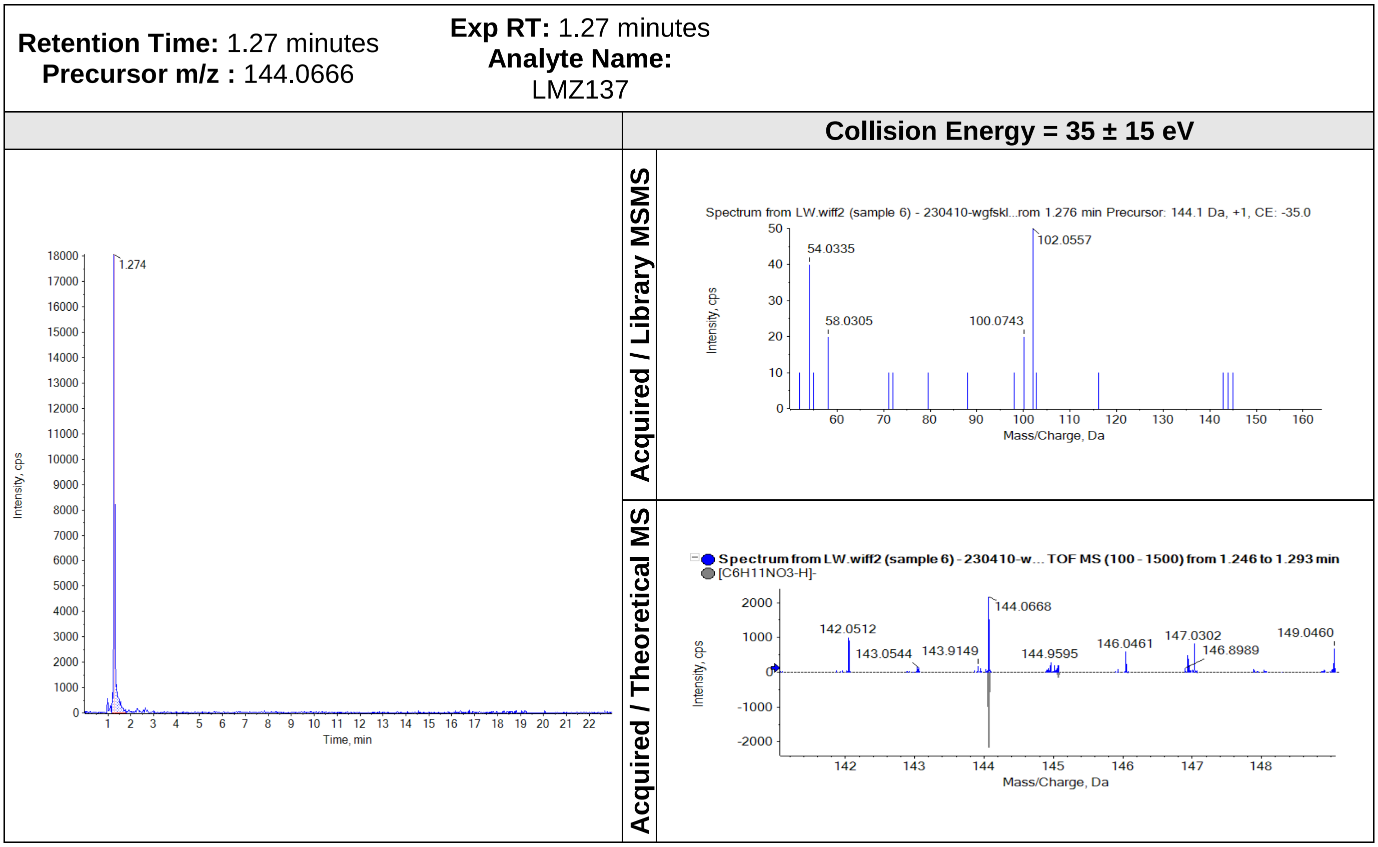

Fig. S3. Detail MS, MS/MS information of compound 3.

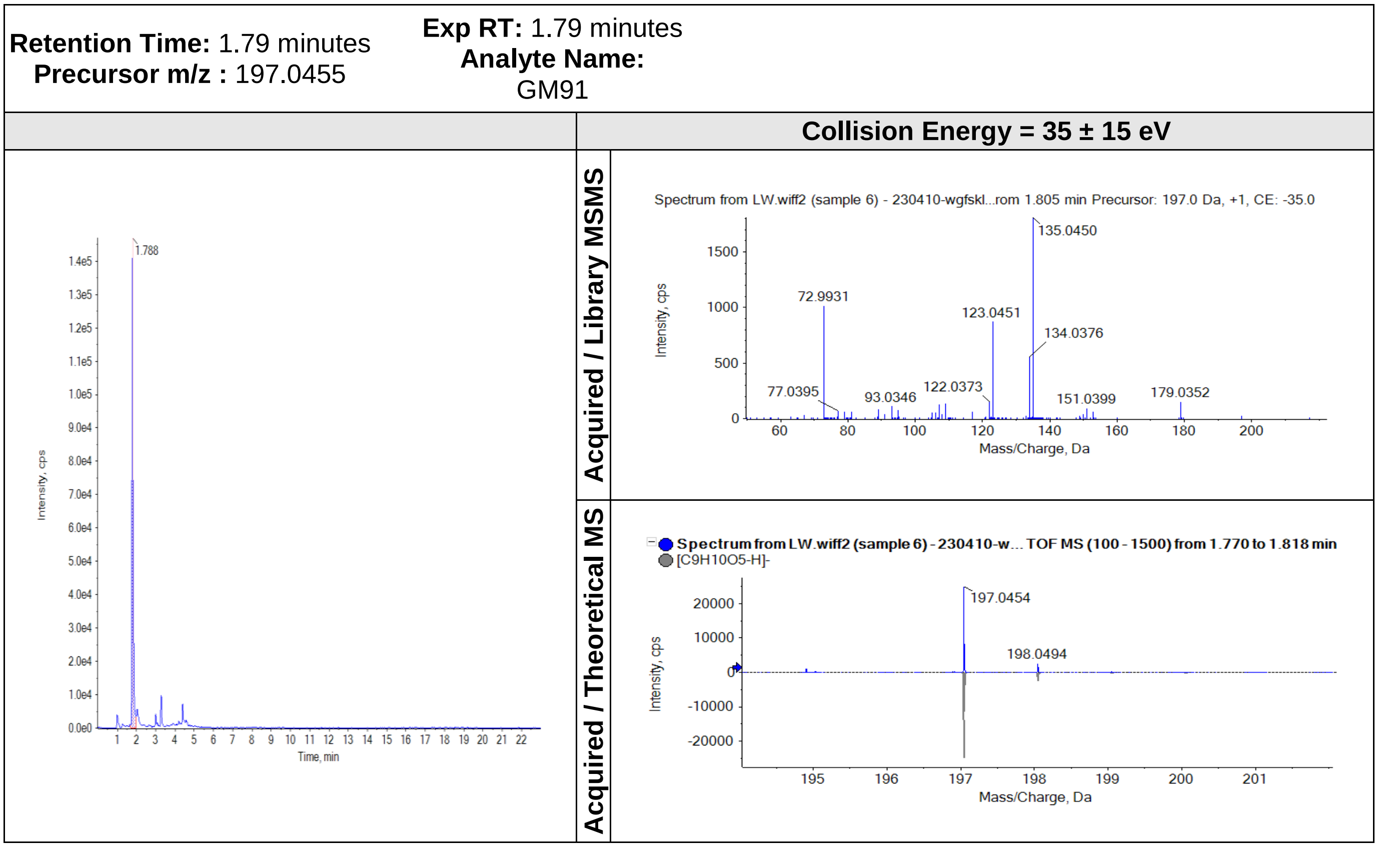

Fig. S4. Detail MS, MS/MS information of compound 4

Retention Time: 2.19 minutes
Precursor m/z : 153.0193

Exp RT: 2.20 minutes
Analyte Name:
CEC11

Collision Energy = 35 ± 15 eV

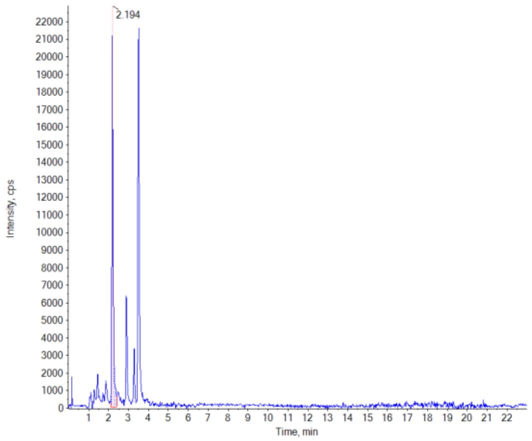

Acquired / Library MSMS

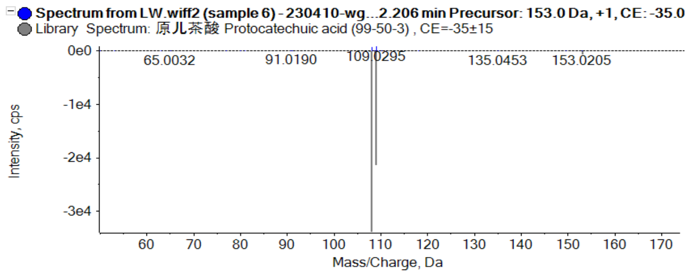

Acquired / Theoretical MS

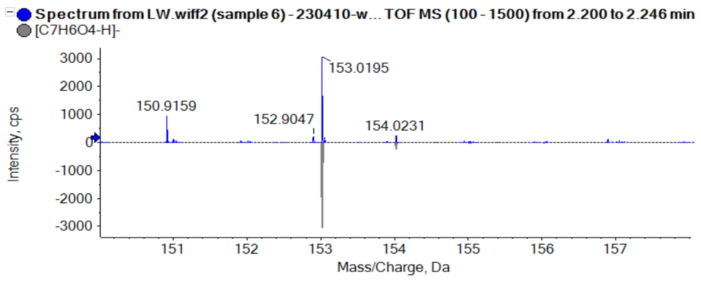

Fig. S5. Detail MS, MS/MS information of compound 6

Retention Time: 2.39 minutes
Precursor m/z : 181.0506

Exp RT: 2.39 minutes
Analyte Name:
GM145

Collision Energy = 35 ± 15 eV

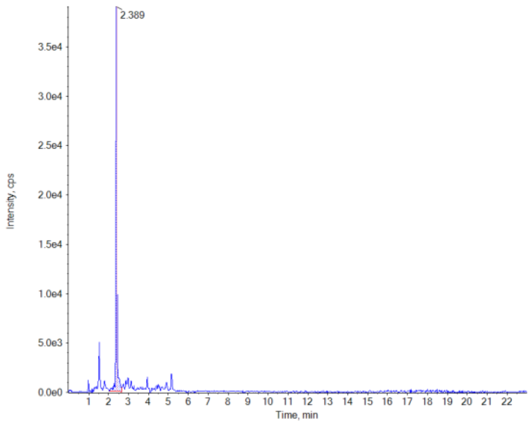

Acquired / Library MSMS

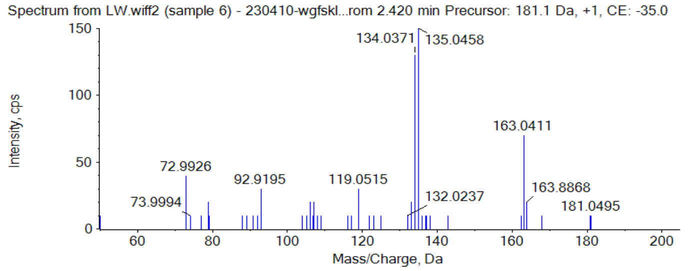

Acquired / Theoretical MS

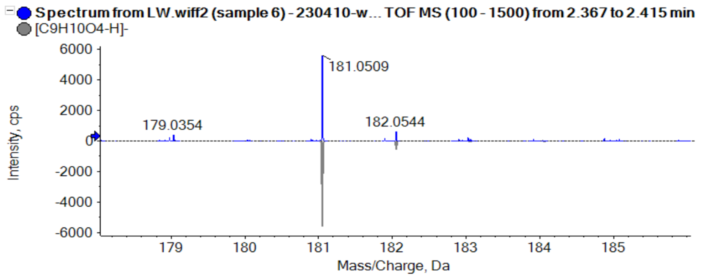

Fig. S6. Detail MS, MS/MS information of compound 7

Retention Time: 2.53 minutes
Precursor m/z : 241.0718

Exp RT: 2.53 minutes
Analyte Name:
GM134

Collision Energy = 35 ± 15 eV

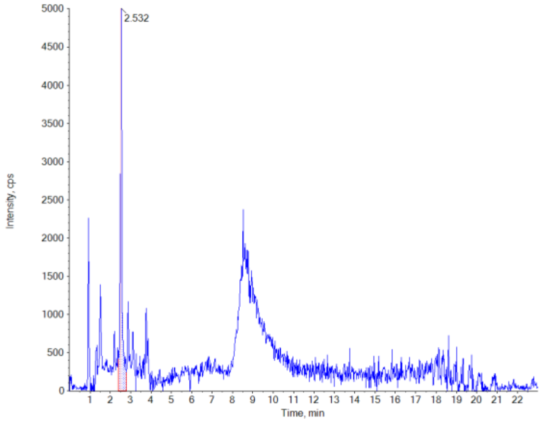

Acquired / Library MSMS

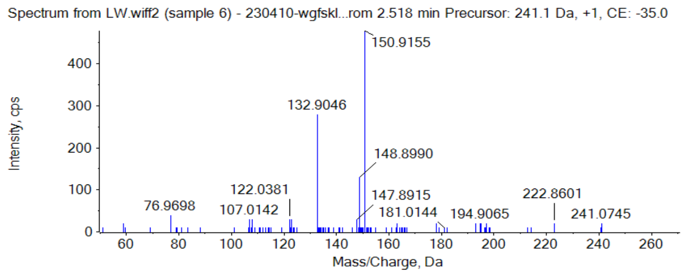

Acquired / Theoretical MS

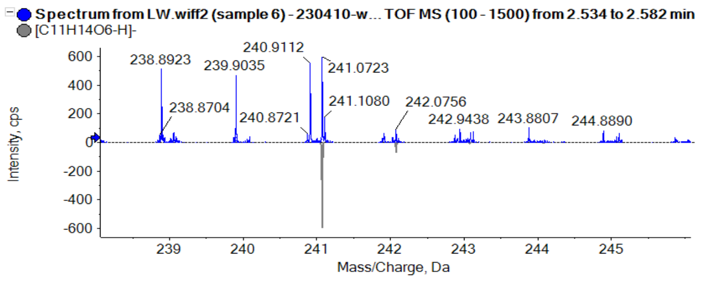

Fig. S7. Detail MS, MS/MS information of compound 8

Retention Time: 2.54 minutes
Precursor m/z : 146.0600

Exp RT: 2.53 minutes
Analyte Name:
LMZ83

Collision Energy = 35 ± 15 eV

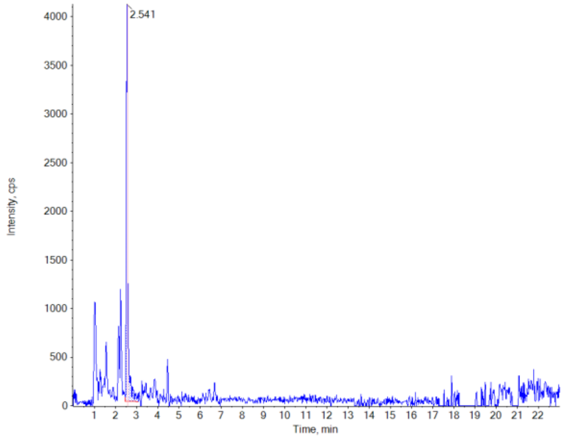

Acquired / Library MSMS

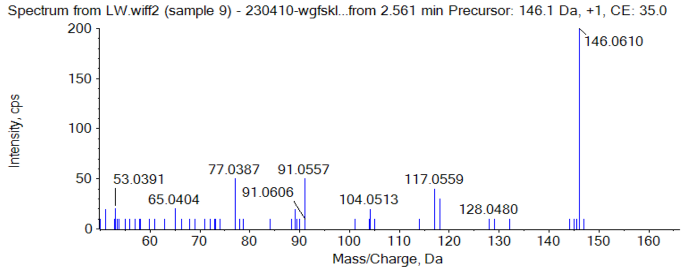

Acquired / Theoretical MS

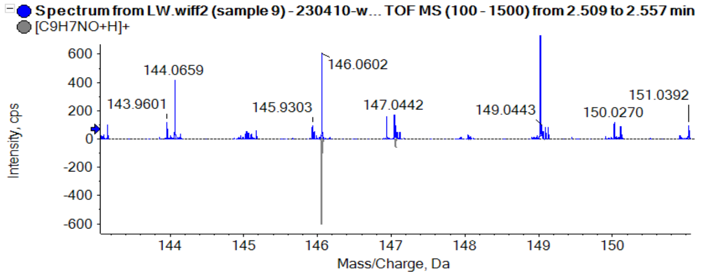

Fig. S8. Detail MS, MS/MS information of compound 9

Retention Time: 2.76 minutes
Precursor m/z : 353.0878

Exp RT: 2.77 minutes
Analyte Name:
CEC9

Collision Energy = 35 ± 15 eV

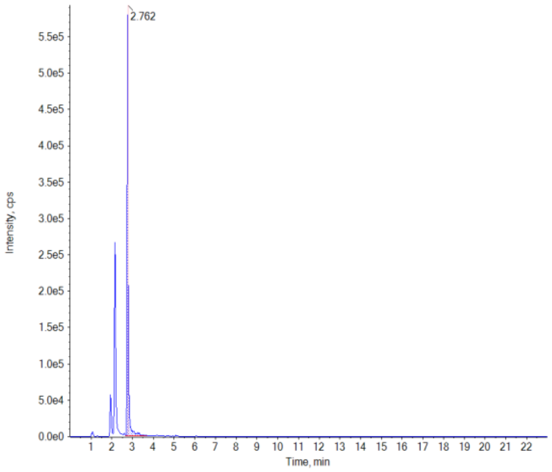

Acquired / Library MSMS

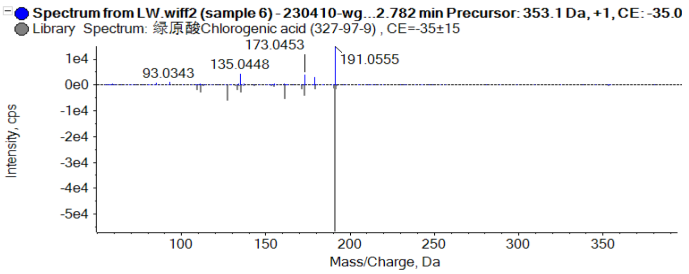

Acquired / Theoretical MS

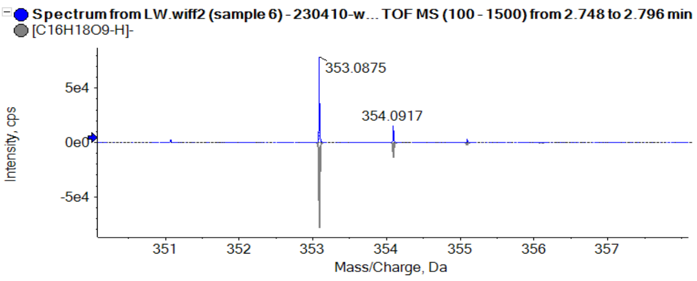

Fig. S9. Detail MS, MS/MS information of compound 10

Retention Time: 3.08 minutes
Precursor m/z : 151.0401

Exp RT: 3.08 minutes
Analyte Name:
CEC25

Collision Energy = 35 ± 15 eV

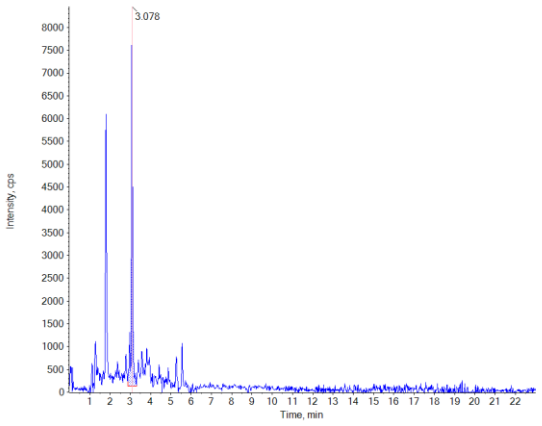

Acquired / Library MSMS

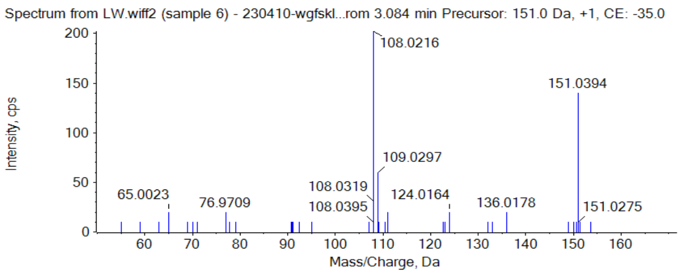

Acquired / Theoretical MS

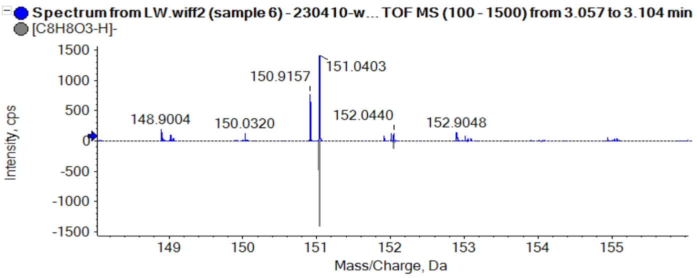

Fig. S11 Detail MS, MS/MS information of compound 12

Retention Time: 3.09 minutes **Exp RT:** 3.08 minutes
Precursor m/z : 177.0193 **Analyte Name:** GM111
Fit (%) N/A **RFit (%)** N/A

Collision Energy = 35 ± 15 eV

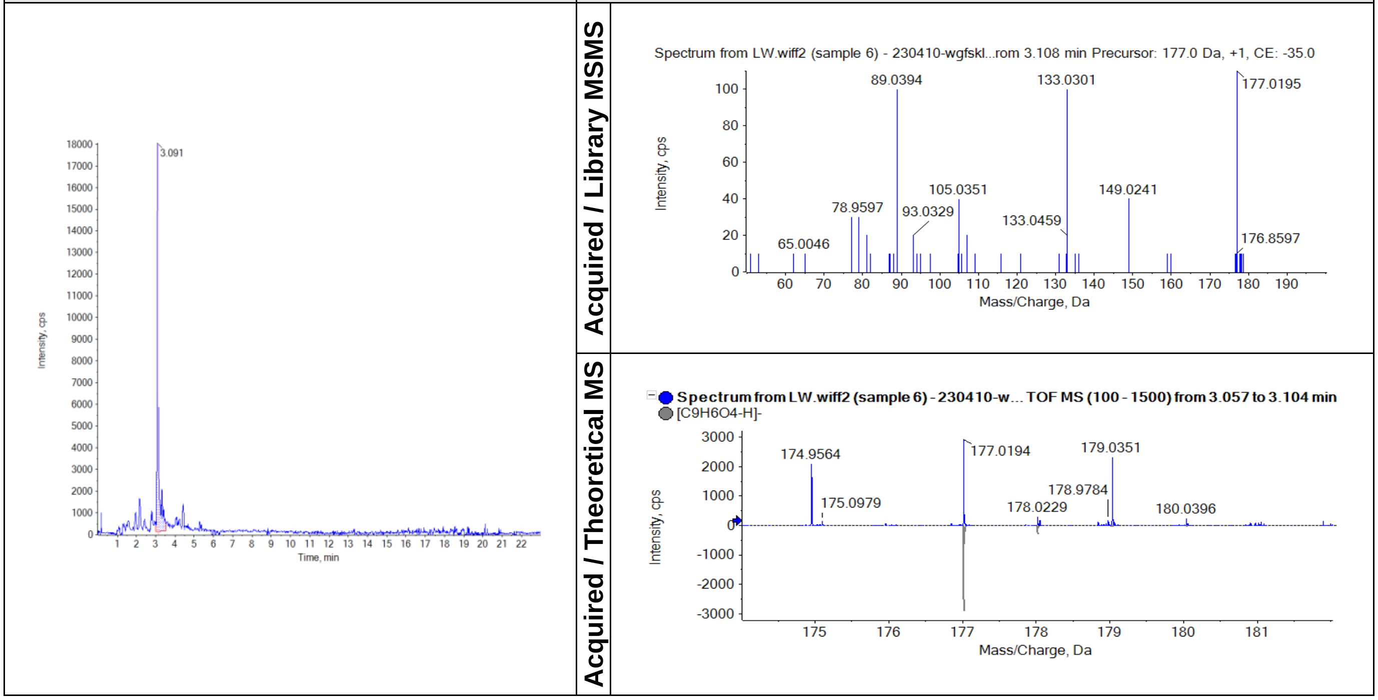

Fig. S12 Detail MS, MS/MS information of compound 13

Retention Time: 3.14 minutes **Exp RT:** 3.14 minutes
Precursor m/z : 179.0350 **Analyte Name:** CEC1

Collision Energy = 35 ± 15 eV

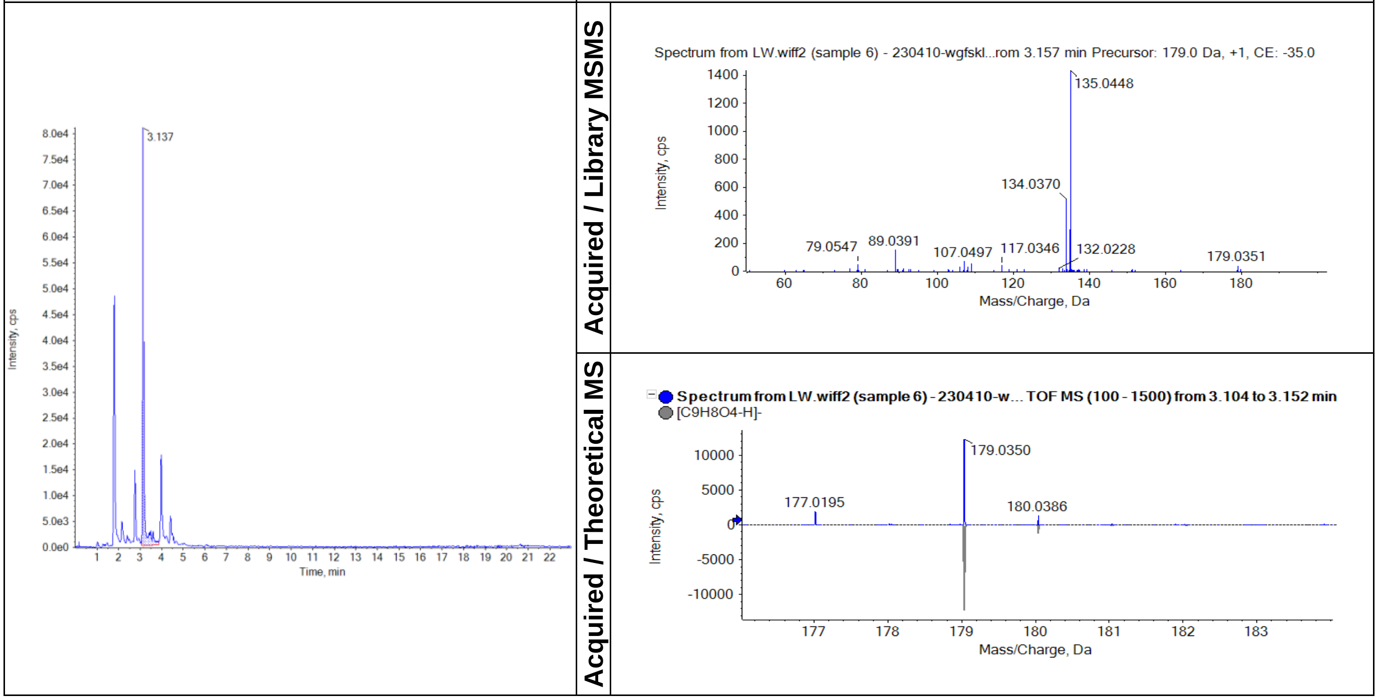

Fig. S13 Detail MS, MS/MS information of compound 14

**Retention Time:** 3.24 minutes<br>**Precursor m/z :** 515.1195	**Exp RT:** 3.25 minutes<br>**Analyte Name:**<br>CEC16
	**Collision Energy = 35 ± 15 eV**

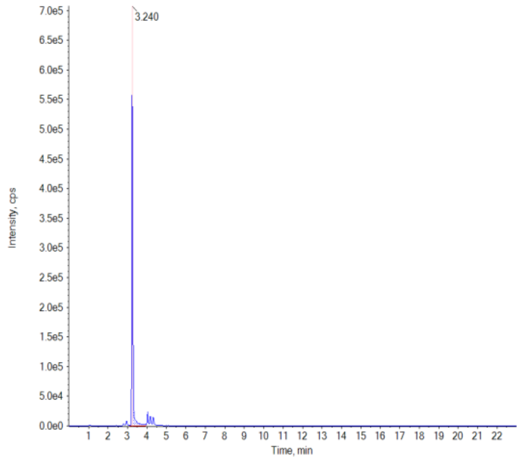

Acquired / Library MSMS

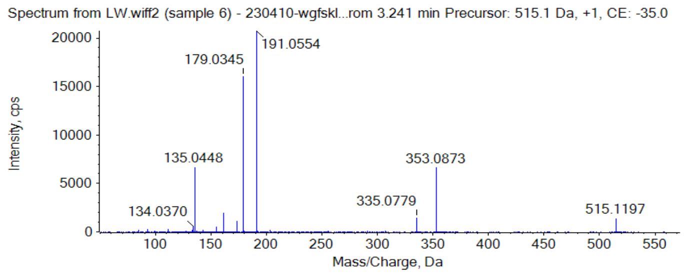

Acquired / Theoretical MS

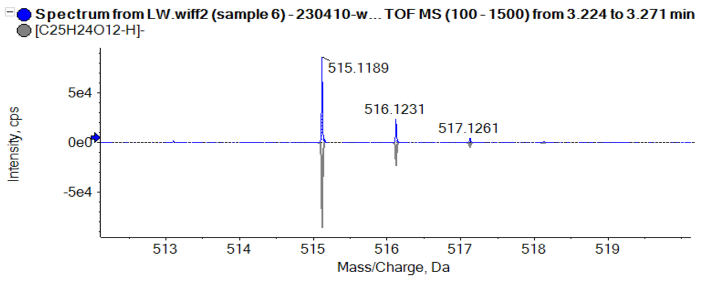

Fig. S14 Detail MS, MS/MS information of compound 15

**Retention Time:** 3.40 minutes<br>**Precursor m/z :** 787.2666	**Exp RT:** 3.39 minutes<br>**Analyte Name:**<br>GM108
	**Collision Energy = 35 ± 15 eV**

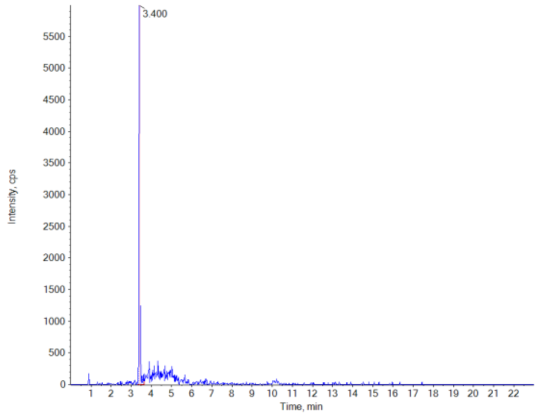

Acquired / Library MSMS

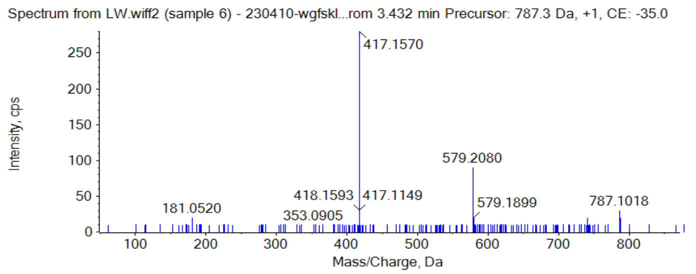

Acquired / Theoretical MS

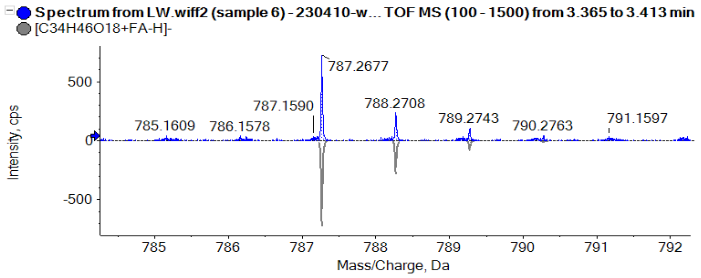

Fig. S15. Detail MS, MS/MS information of compound 16.

Retention Time: 3.61 minutes
Precursor m/z : 183.0299

Exp RT: 3.60 minutes
Analyte Name:
GM144

Collision Energy = 35 ± 15 eV

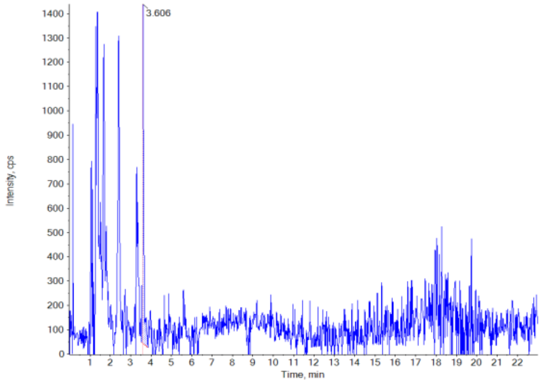

Acquired / Library MSMS

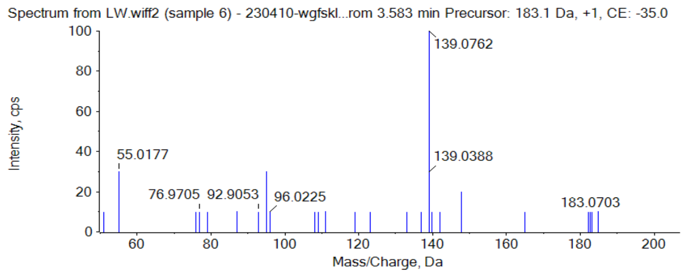

Acquired / Theoretical MS

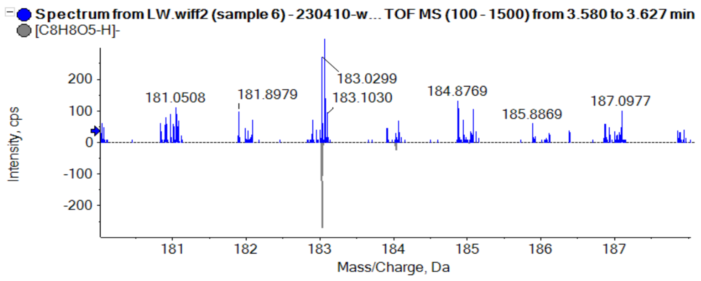

Fig. S16 Detail MS, MS/MS information of compound 17.

Retention Time: 3.67 minutes
Precursor m/z : 529.1351

Exp RT: 3.68 minutes
Analyte Name:
GM140

Collision Energy = 35 ± 15 eV

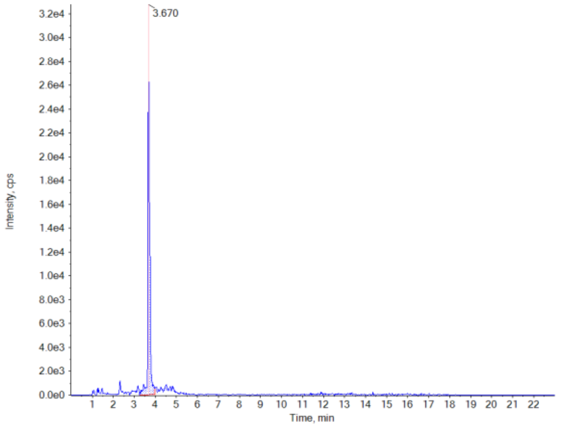

Acquired / Library MSMS

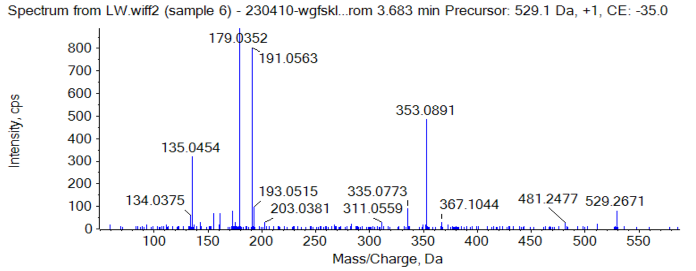

Acquired / Theoretical MS

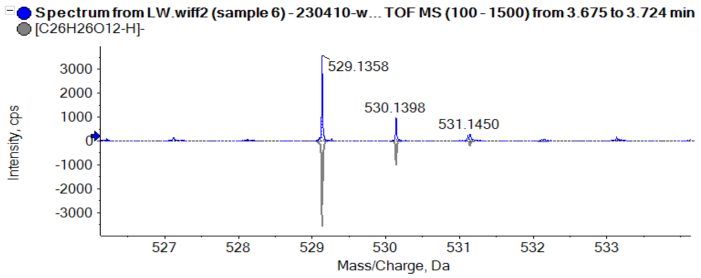

Fig. S17 Detail MS, MS/MS information of compound 18

Retention Time: 3.69 minutes
Precursor m/z : 206.0823

Exp RT: 3.68 minutes
Analyte Name:
LMZ87

Collision Energy = 35 ± 15 eV

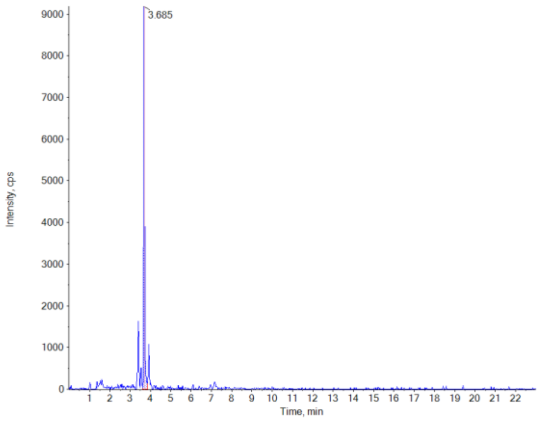

Acquired / Library MSMS

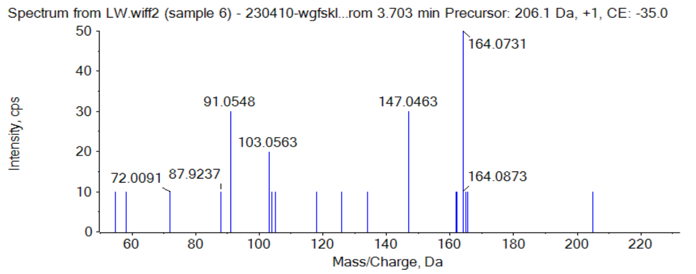

Acquired / Theoretical MS

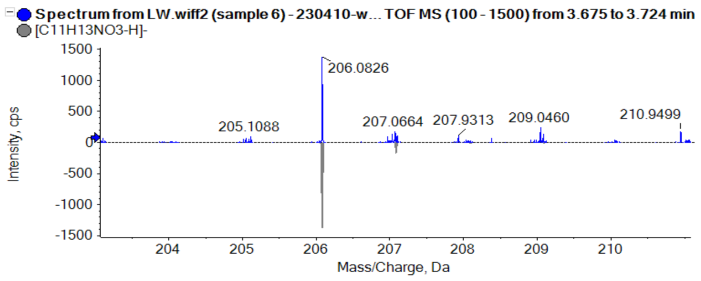

Fig. S18 Detail MS, MS/MS information of compound 19

Retention Time: 3.69 minutes
Precursor m/z : 291.0874

Exp RT: 3.70 minutes
Analyte Name:
LMZ106

Collision Energy = 35 ± 15 eV

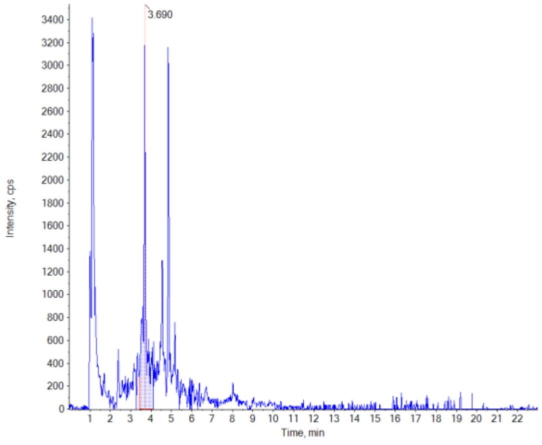

Acquired / Library MSMS

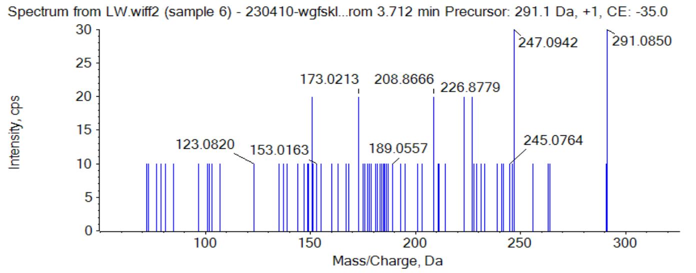

Acquired / Theoretical MS

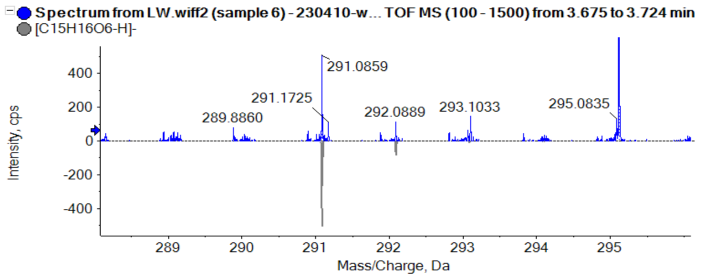

Fig. S19 Detail MS, MS/MS information of compound 20

Retention Time: 3.70 minutes
Precursor m/z : 551.2134

Exp RT: 3.70 minutes
Analyte Name:
GM105

Collision Energy = 35 ± 15 eV

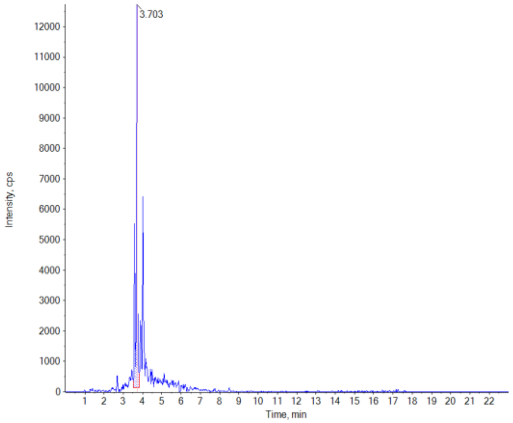

Acquired / Library MSMS

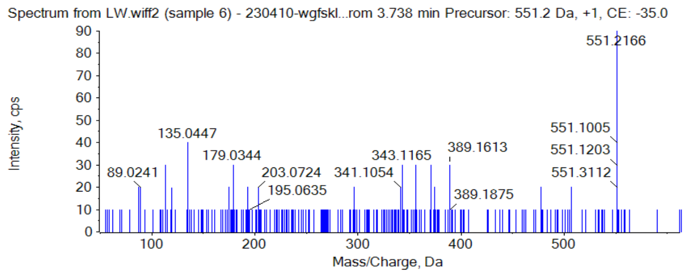

Acquired / Theoretical MS

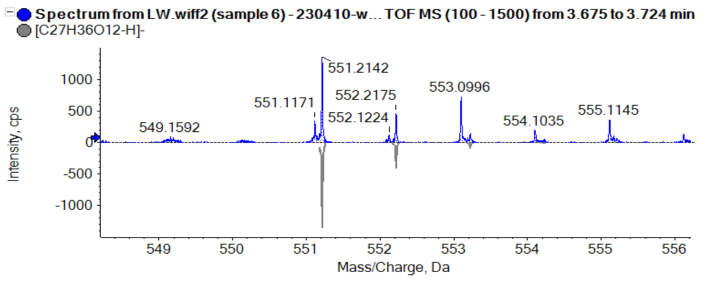

Fig. S20 Detail MS, MS/MS information of compound 21

Retention Time: 3.73 minutes
Precursor m/z : 521.2028

Exp RT: 3.72 minutes
Analyte Name:
GM84

Collision Energy = 35 ± 15 eV

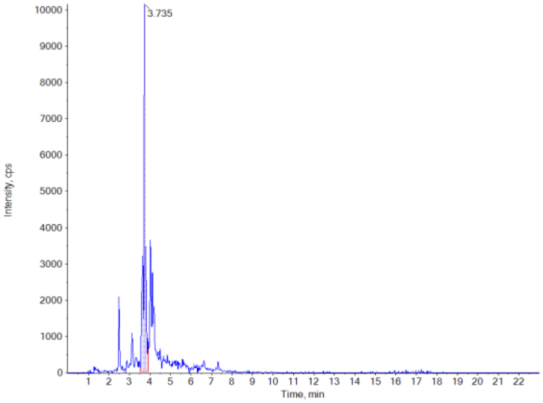

Acquired / Library MSMS

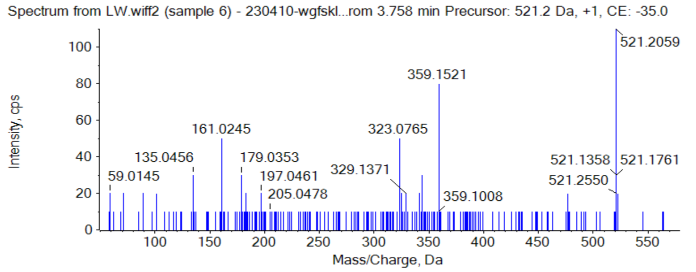

Acquired / Theoretical MS

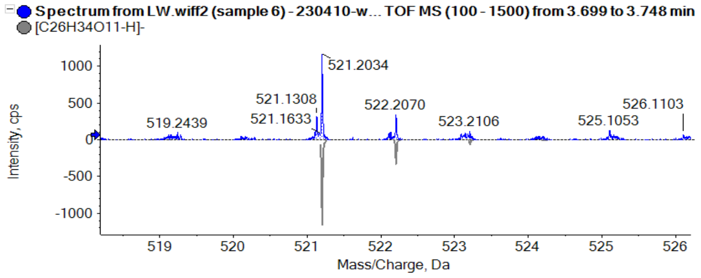

Fig. S21 Detail MS, MS/MS information of compound 22

Retention Time: 3.96 minutes
Precursor m/z : 422.1809

Exp RT: 3.97 minutes
Analyte Name:
LMZ70

Collision Energy = 35 ± 15 eV

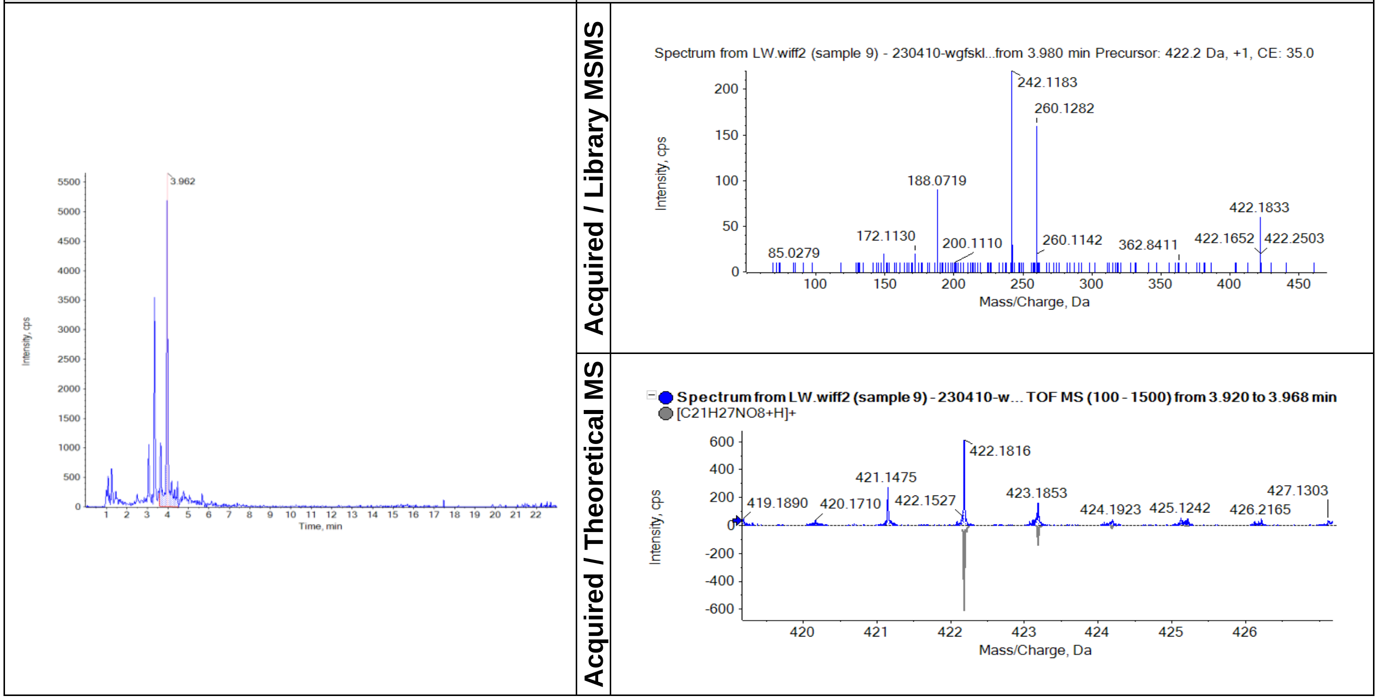

Fig. S22 Detail MS, MS/MS information of compound 23

Retention Time: 4.03 minutes
Precursor m/z : 597.2917

Exp RT: 3.89 minutes
Analyte Name:
TKS18

Collision Energy = 35 ± 15 eV

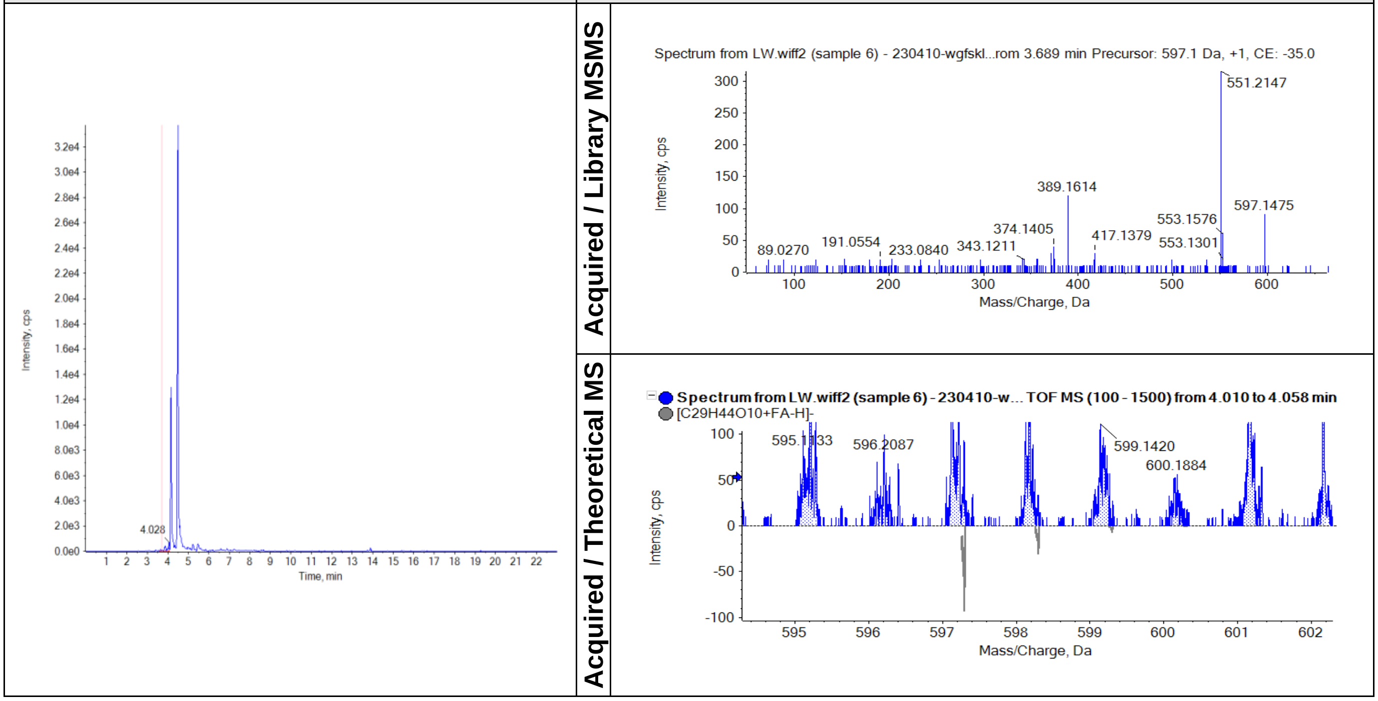

Fig. S23 Detail MS, MS/MS information of compound 24.

**Retention Time:** 4.11 minutes<br>**Precursor m/z :** 611.1981	**Exp RT:** 4.11 minutes<br>**Analyte Name:**<br>GM89
	**Collision Energy = 35 ± 15 eV**

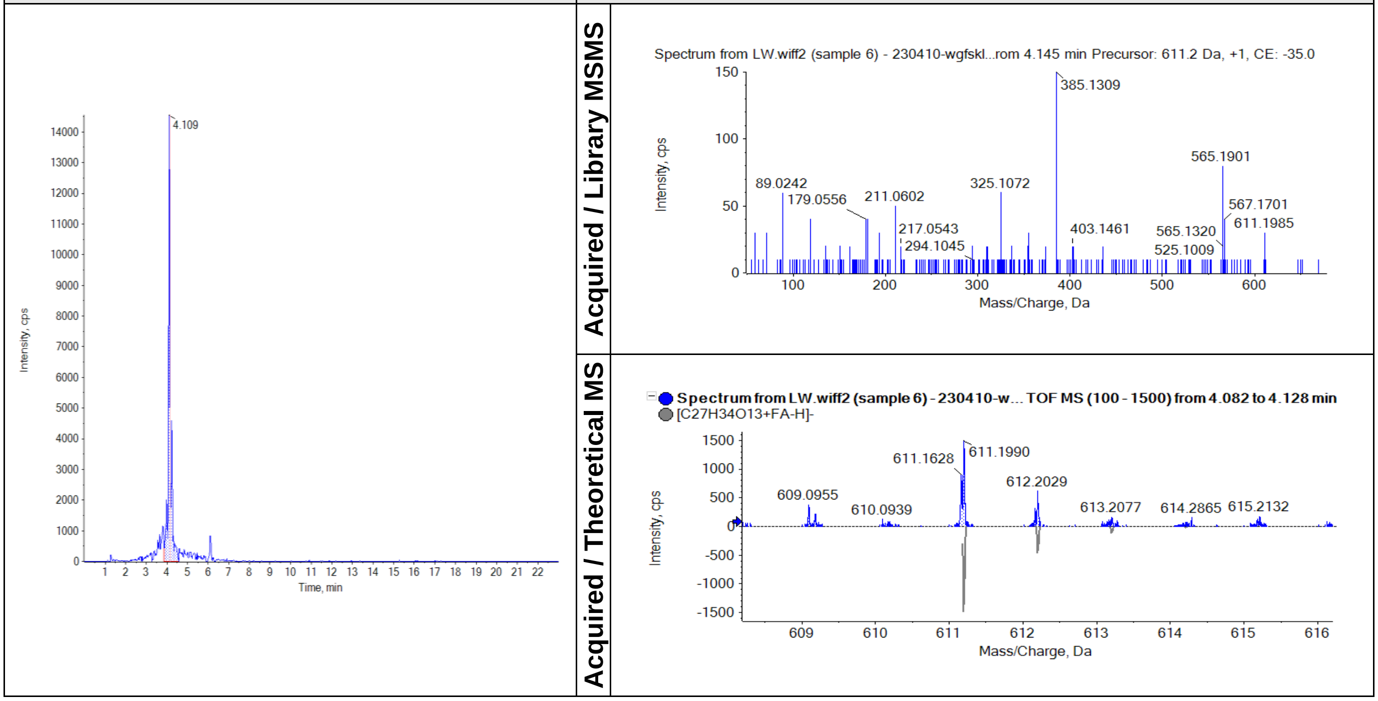

Fig. S24 Detail MS, MS/MS information of compound 25

**Retention Time:** 4.16 minutes<br>**Precursor m/z :** 579.2083	**Exp RT:** 4.15 minutes<br>**Analyte Name:**<br>GM107
	**Collision Energy = 35 ± 15 eV**

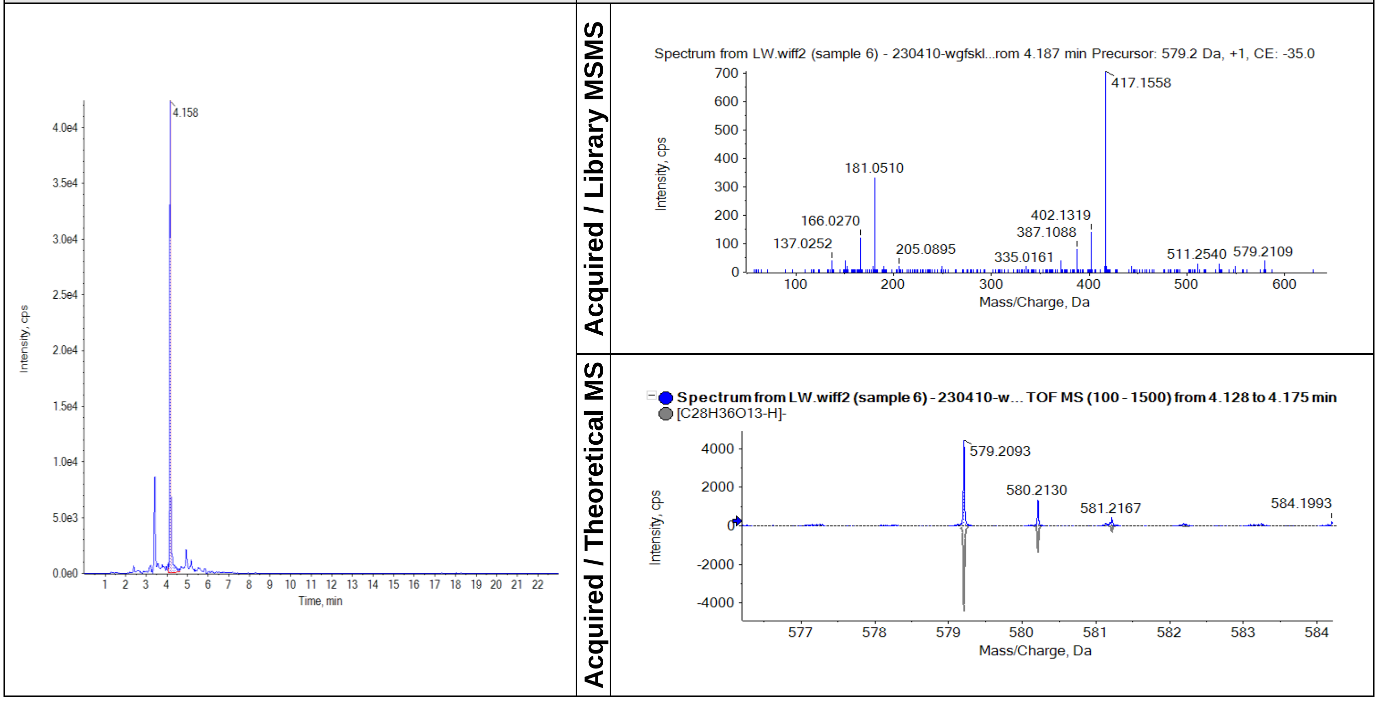

Fig. S25 Detail MS, MS/MS information of compound 27

Retention Time: 4.24 minutes
Precursor m/z : 609.1825

Exp RT: 4.25 minutes
Analyte Name:
TKS52

Collision Energy = 35 ± 15 eV

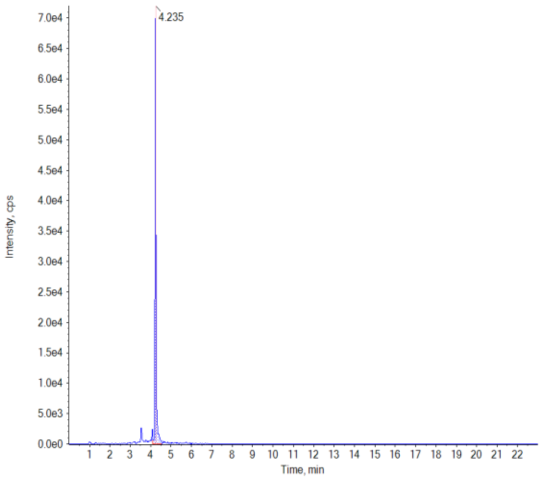

Acquired / Library MSMS

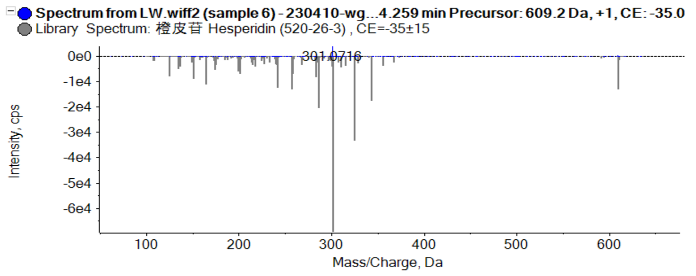

Acquired / Theoretical MS

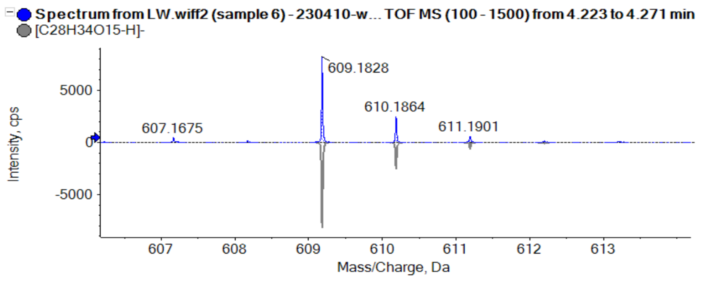

Fig. S26 Detail MS, MS/MS information of compound 28

Retention Time: 4.26 minutes
Precursor m/z : 639.1931

Exp RT: 4.25 minutes
Analyte Name:
GM83

Collision Energy = 35 ± 15 eV

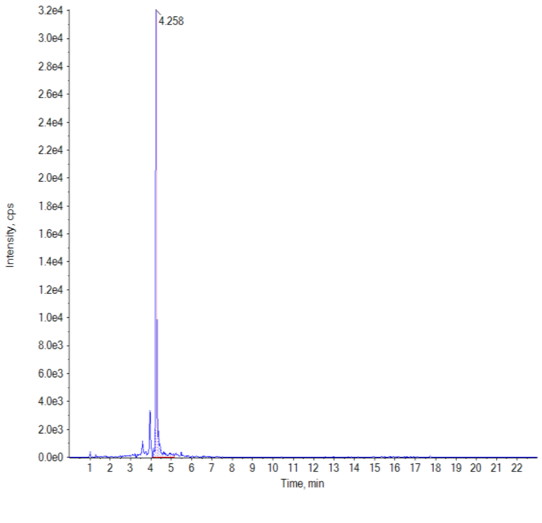

Acquired / Library MSMS

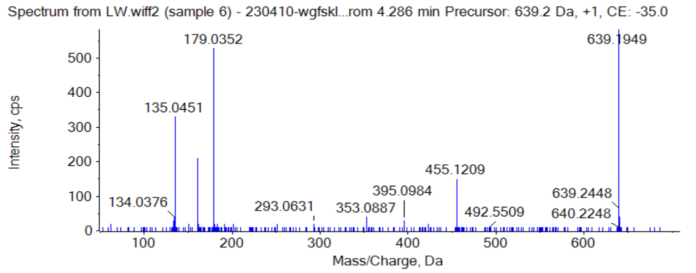

Acquired / Theoretical MS

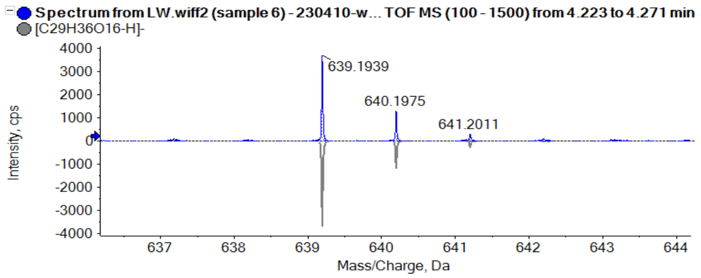

Fig. S27 Detail MS, MS/MS information of compound 29.

**Retention Time:** 4.31 minutes<br>**Precursor m/z :** 743.3496	**Exp RT:** 4.48 minutes<br>**Analyte Name:**<br>TKS79

		**Collision Energy = 35 ± 15 eV**
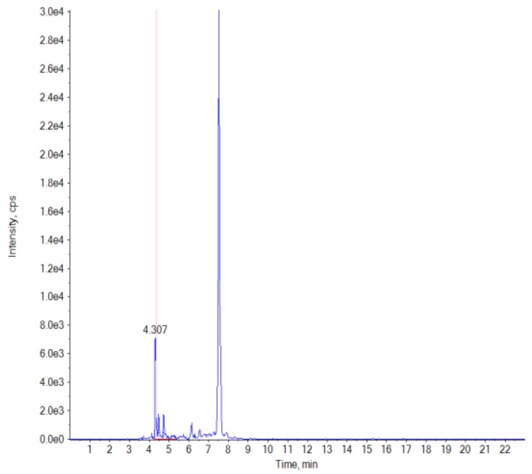	**Acquired / Library MSMS**	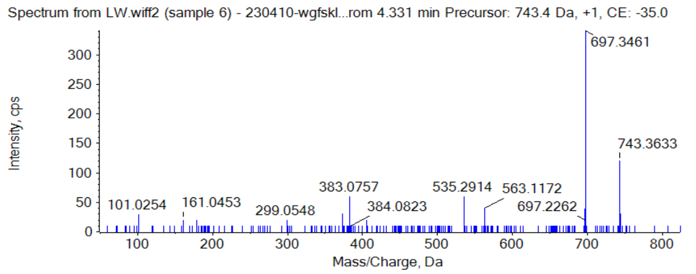
	**Acquired / Theoretical MS**	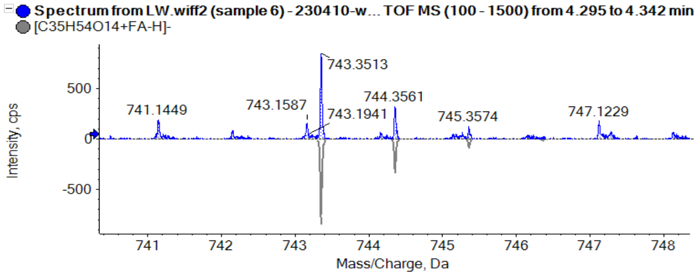

Fig. S28 Detail MS, MS/MS information of compound 31

**Retention Time:** 4.32 minutes<br>**Precursor m/z :** 370.1649	**Exp RT:** 4.33 minutes<br>**Analyte Name:**<br>LMZ88

		**Collision Energy = 35 ± 15 eV**
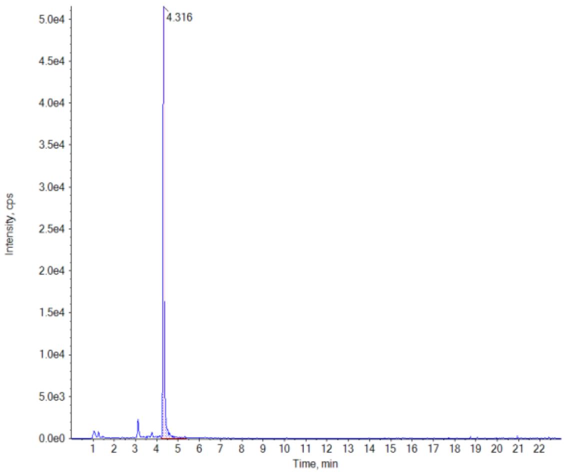	**Acquired / Library MSMS**	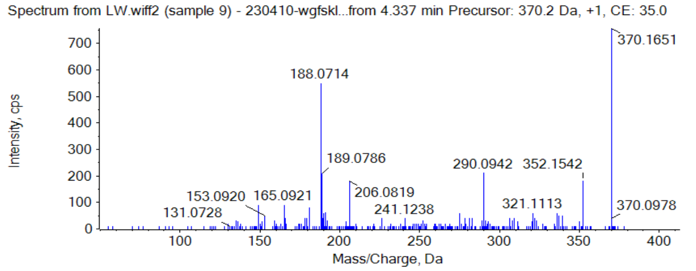
	**Acquired / Theoretical MS**	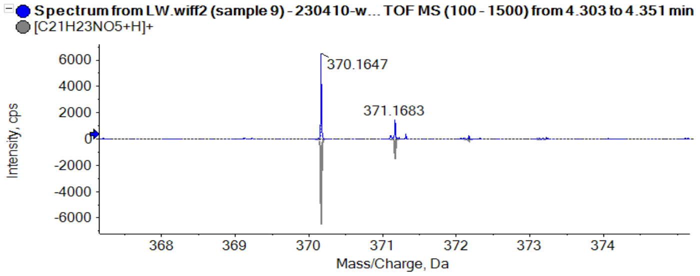

Fig. S29 Detail MS, MS/MS information of compound 32

Retention Time: 4.40 minutes
Precursor m/z : 359.0772

Exp RT: 4.39 minutes
Analyte Name:
SZM17

Collision Energy = 35 ± 15 eV

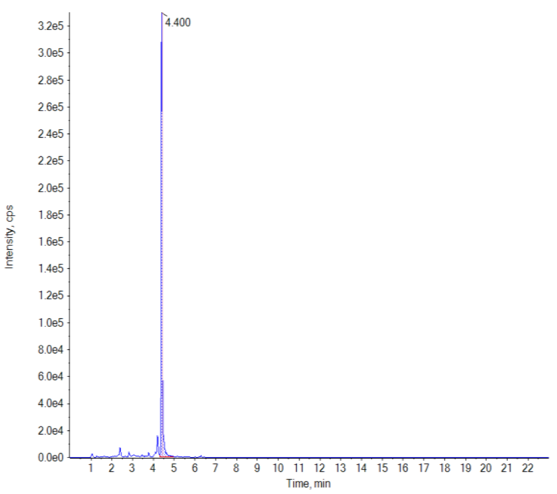

Acquired / Library MSMS

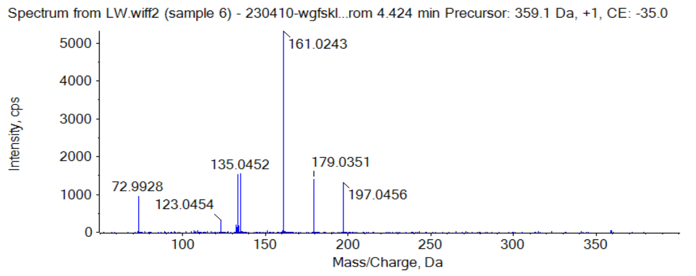

Acquired / Theoretical MS

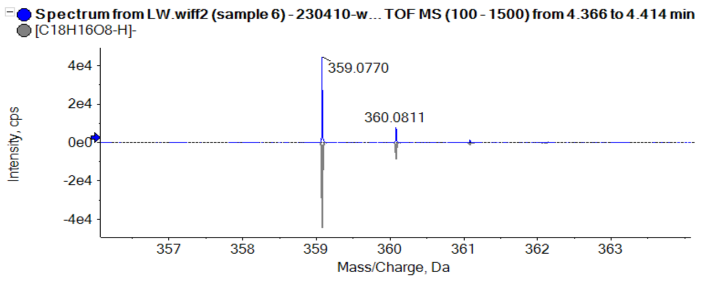

Fig. S30 Detail MS, MS/MS information of compound 33

Retention Time: 4.74 minutes
Precursor m/z : 335.1152

Exp RT: 4.66 minutes
Analyte Name:
LMZ24

Collision Energy = 35 ± 15 eV

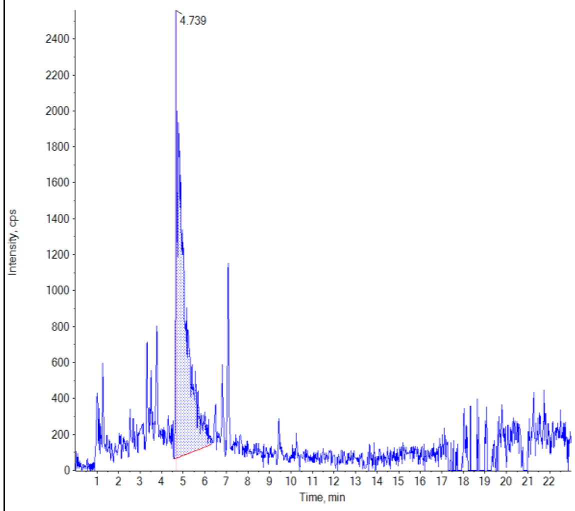

Acquired / Library MSMS

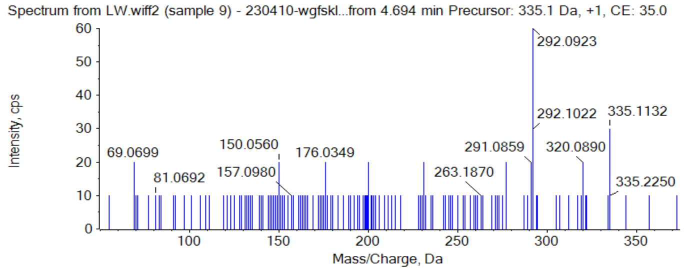

Acquired / Theoretical MS

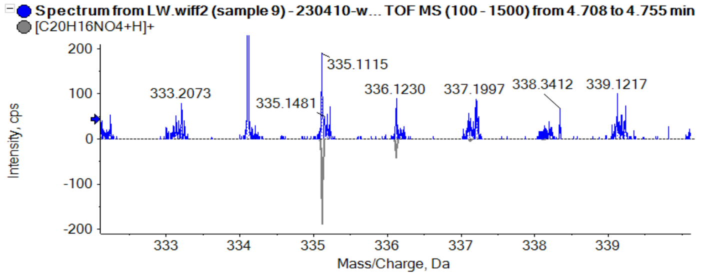

Fig. S31 Detail MS, MS/MS information of compound 35

Retention Time: 4.84 minutes
Precursor m/z : 334.1074

Exp RT: 4.78 minutes
Analyte Name: LMZ30

Collision Energy = 35 ± 15 eV

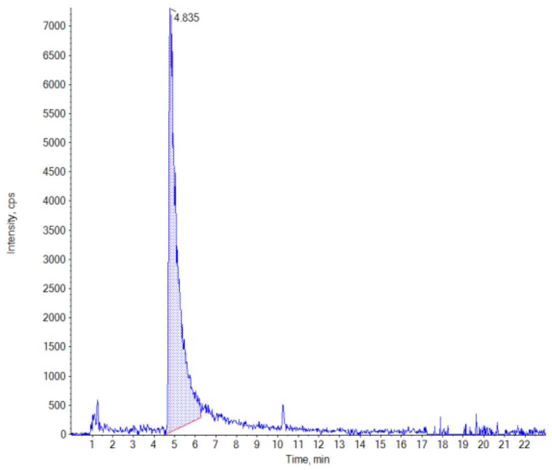

Acquired / Library MSMS

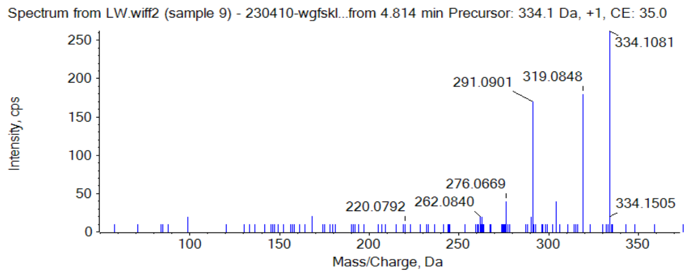

Acquired / Theoretical MS

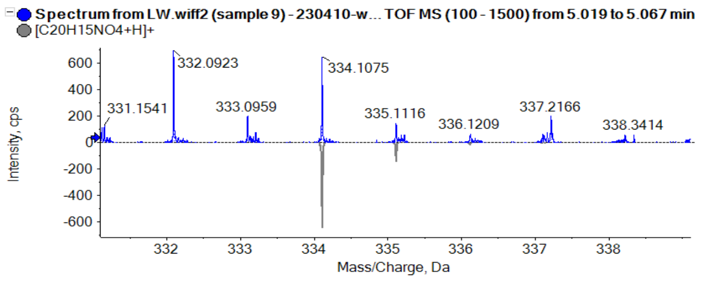

Fig. S32 Detail MS, MS/MS information of compound 36

Retention Time: 4.88 minutes
Precursor m/z : 825.4278

Exp RT: 4.89 minutes
Analyte Name: GM127

Collision Energy = 35 ± 15 eV

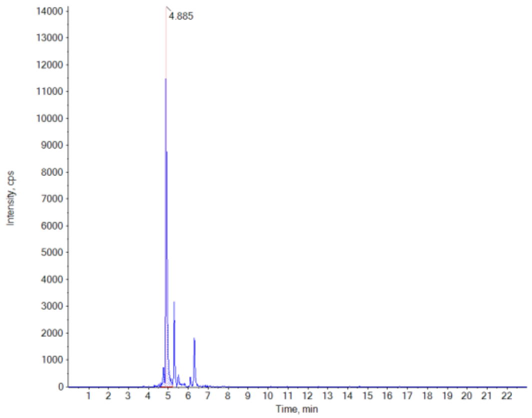

Acquired / Library MSMS

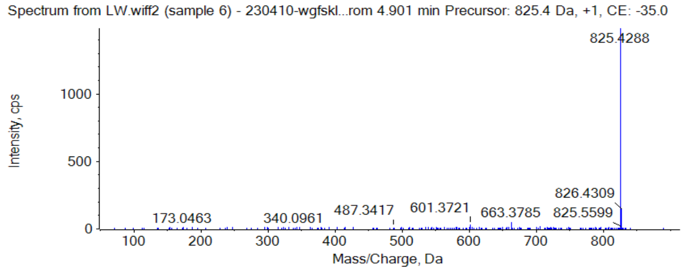

Acquired / Theoretical MS

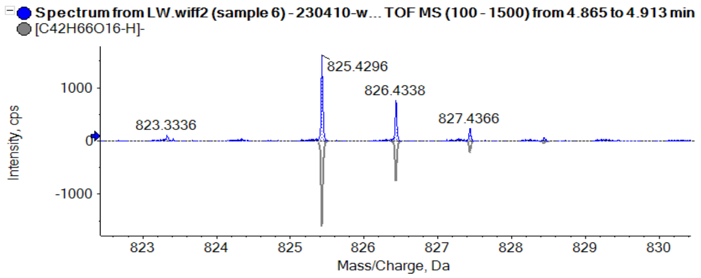

Fig. S33 Detail MS, MS/MS information of compound 37.

Retention Time: 5.19 minutes
Precursor m/z : 333.0996

Exp RT: 5.11 minutes
Analyte Name:
LMZ26

Collision Energy = 35 ± 15 eV

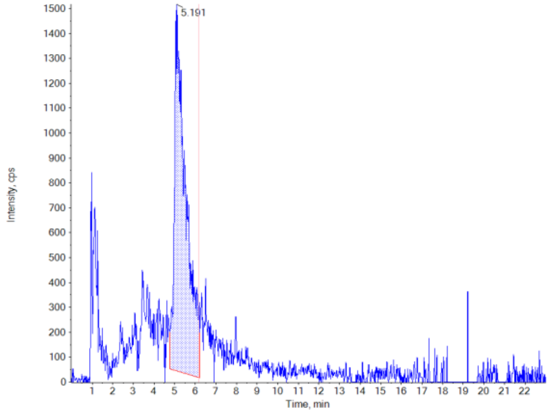

Acquired / Library MSMS

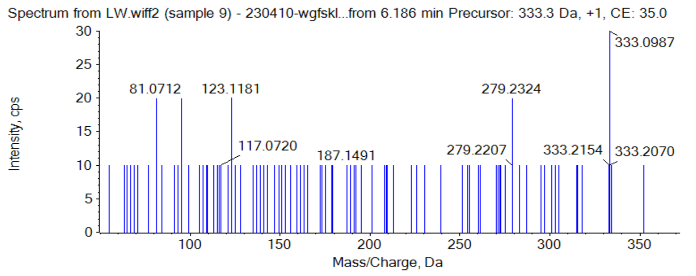

Acquired / Theoretical MS

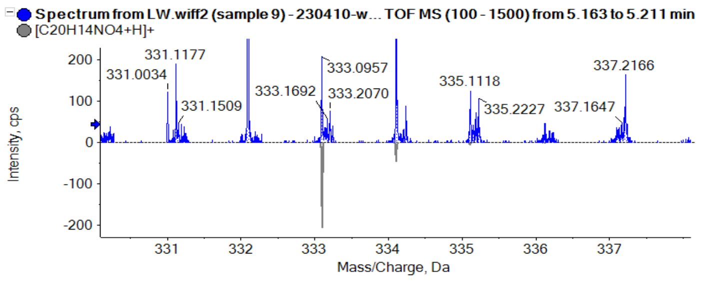

Fig. S34 Detail MS, MS/MS information of compound 38

Retention Time: 5.26 minutes
Precursor m/z : 167.0350

Exp RT: 5.27 minutes
Analyte Name:
GM143

Collision Energy = 35 ± 15 eV

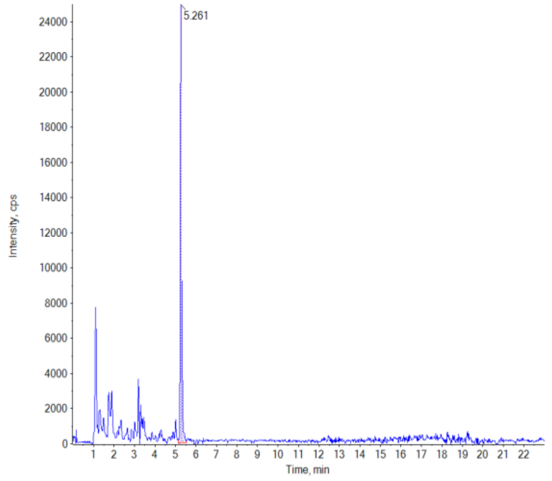

Acquired / Library MSMS

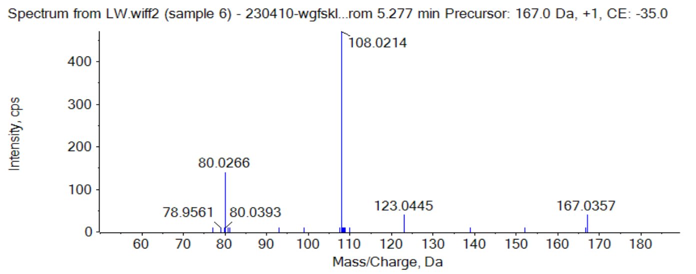

Acquired / Theoretical MS

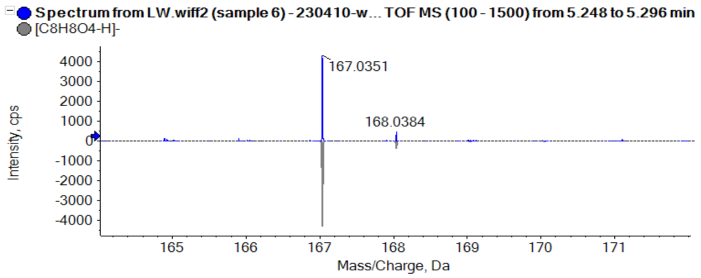

Fig. S35 Detail MS, MS/MS information of compound 39

Retention Time: 5.30 minutes
Precursor m/z : 285.0405

Exp RT: 5.30 minutes
Analyte Name: GM76

Collision Energy = 35 ± 15 eV

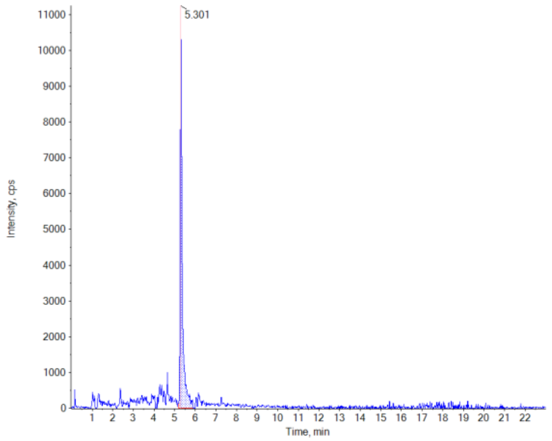

Acquired / Library MSMS

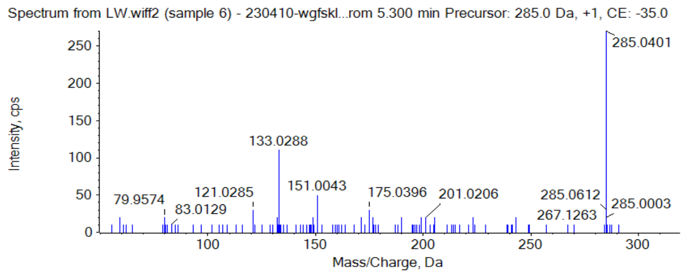

Acquired / Theoretical MS

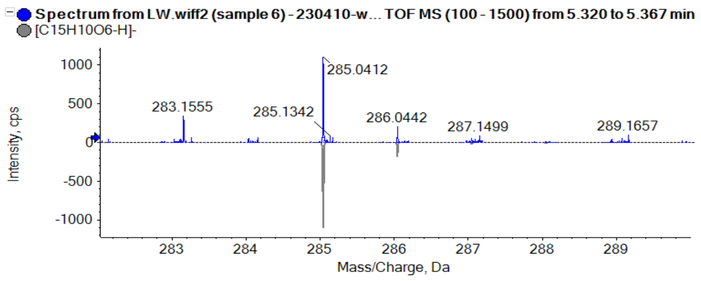

Fig. S36 Detail MS, MS/MS information of compound 40

Retention Time: 5.31 minutes
Precursor m/z : 911.5010

Exp RT: 5.34 minutes
Analyte Name: GM119

Collision Energy = 35 ± 15 eV

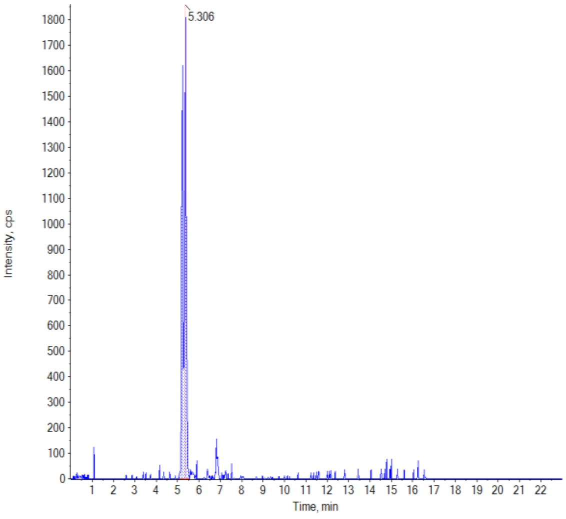

Acquired / Library MSMS

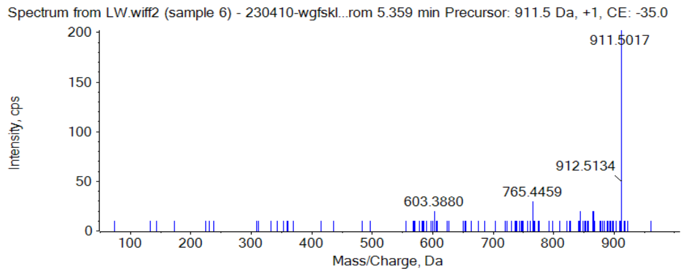

Acquired / Theoretical MS

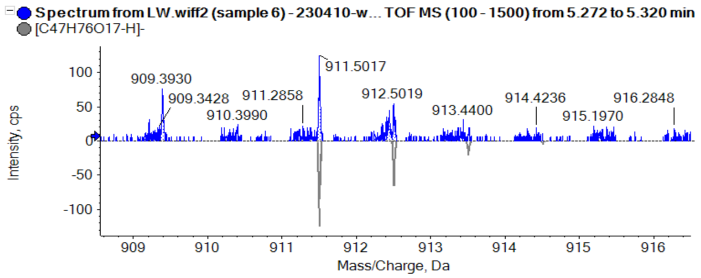

Fig. S37 Detail MS, MS/MS information of compound 41.

Retention Time: 5.50 minutes
Precursor m/z : 809.4329

Exp RT: 5.51 minutes
Analyte Name:
GM122

Collision Energy = 35 ± 15 eV

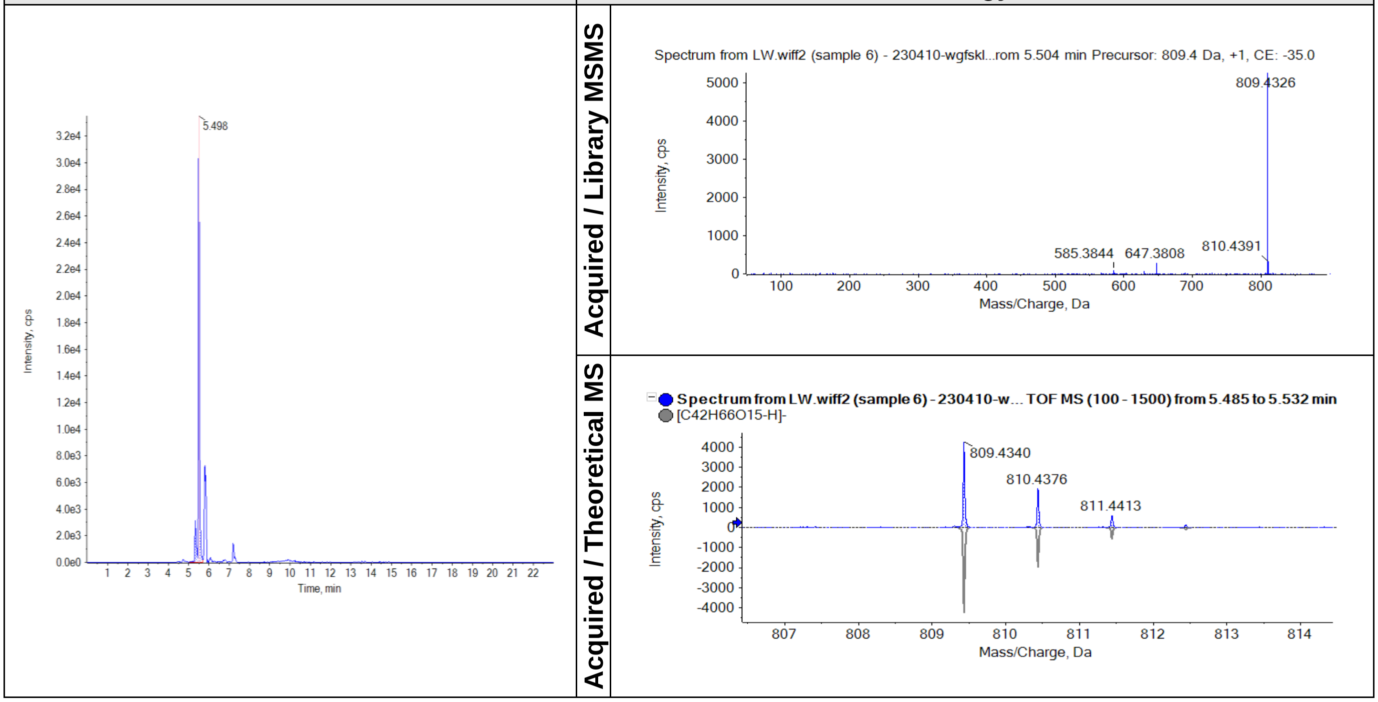

Fig. S38 Detail MS, MS/MS information of compound 42.

Retention Time: 5.60 minutes
Precursor m/z : 190.0863

Exp RT: 5.59 minutes
Analyte Name:
LMZ64

Collision Energy = 35 ± 15 eV

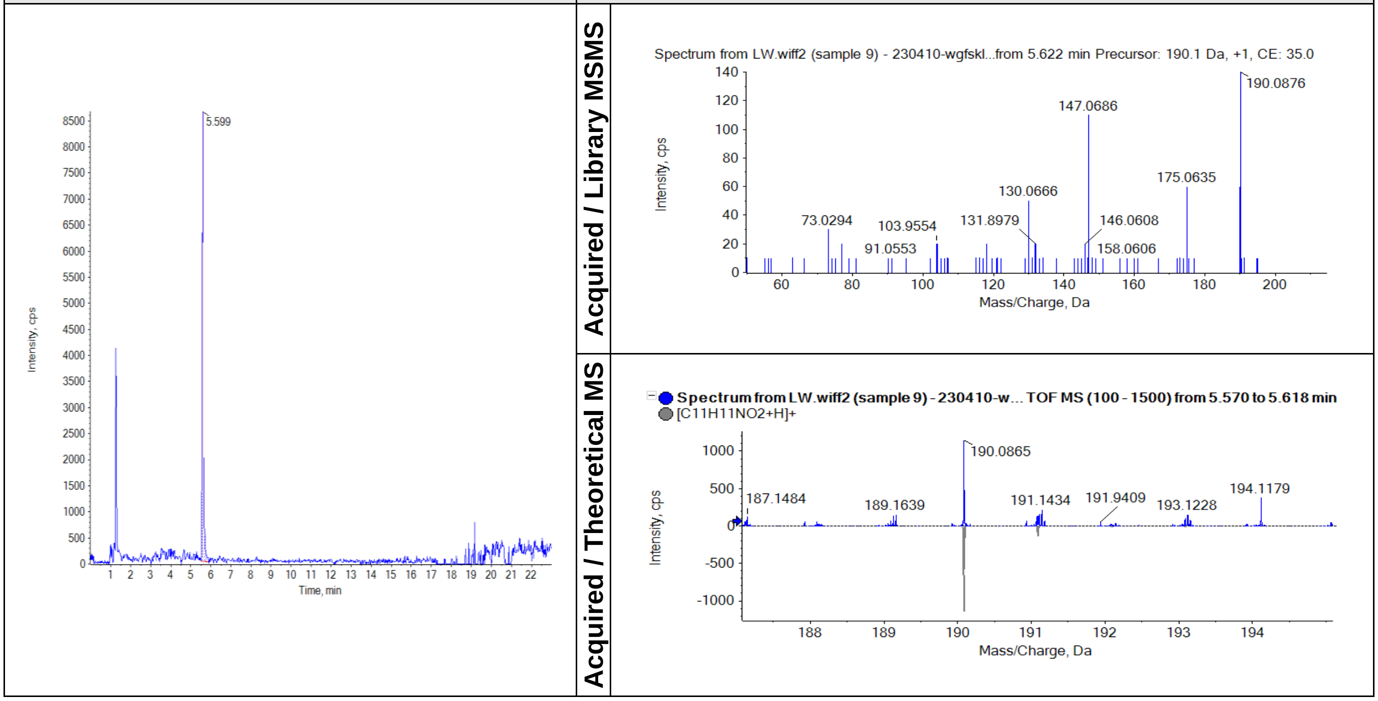

Fig. S39 Detail MS, MS/MS information of compound 43

**Retention Time:** 5.90 minutes<br>**Precursor m/z :** 889.3897	**Exp RT:** 5.89 minutes<br>**Analyte Name:**<br>GM147
	**Collision Energy = 35 ± 15 eV**
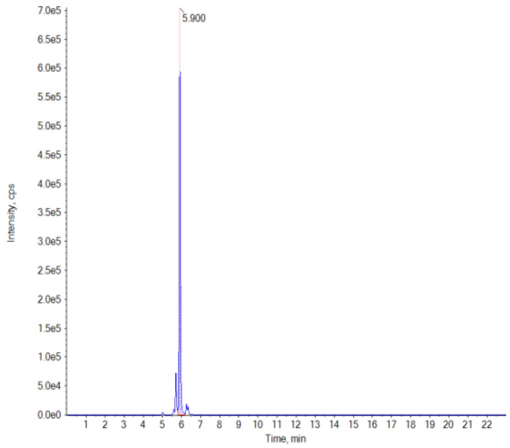	**Acquired / Library MSMS** 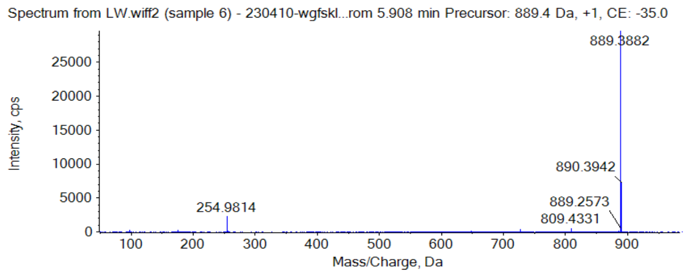<br>**Acquired / Theoretical MS** 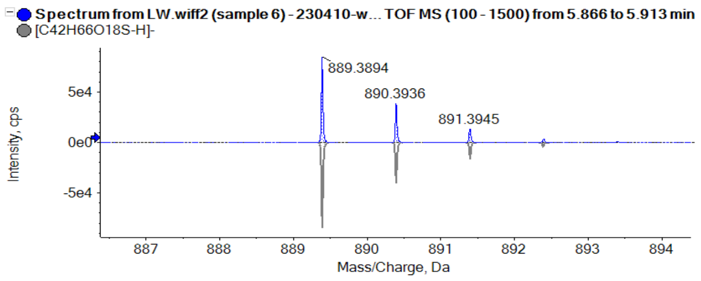

Fig. S40 Detail MS, MS/MS information of compound 44

**Retention Time:** 6.02 minutes<br>**Precursor m/z :** 725.3754	**Exp RT:** 6.08 minutes<br>**Analyte Name:**<br>GM171
	**Collision Energy = 35 ± 15 eV**
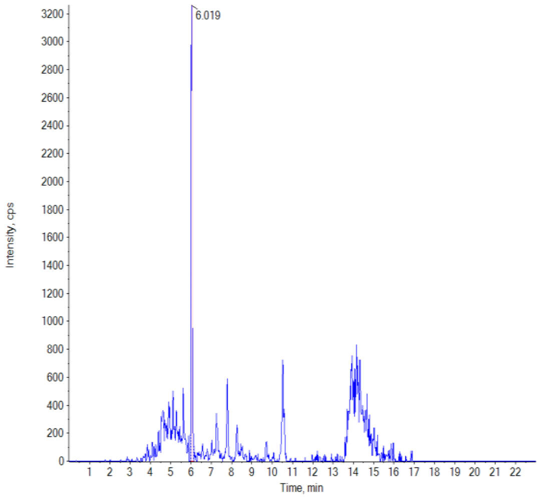	**Acquired / Library MSMS** 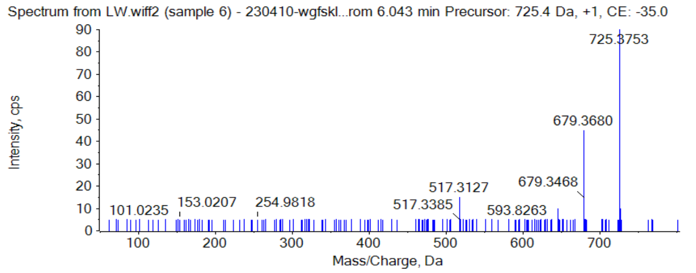<br>**Acquired / Theoretical MS** 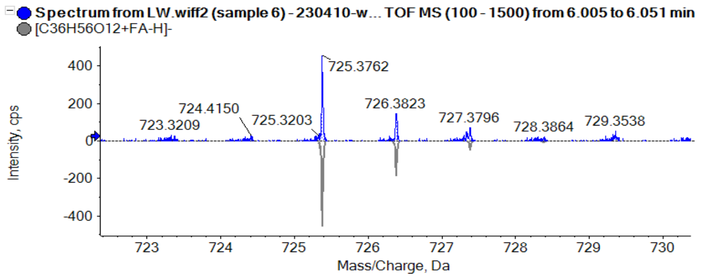

Fig. S41 Detail MS, MS/MS information of compound 45.

Retention Time: 6.30 minutes
Precursor m/z : 925.3567

Exp RT: 6.29 minutes
Analyte Name:
GM172

Collision Energy = 35 ± 15 eV

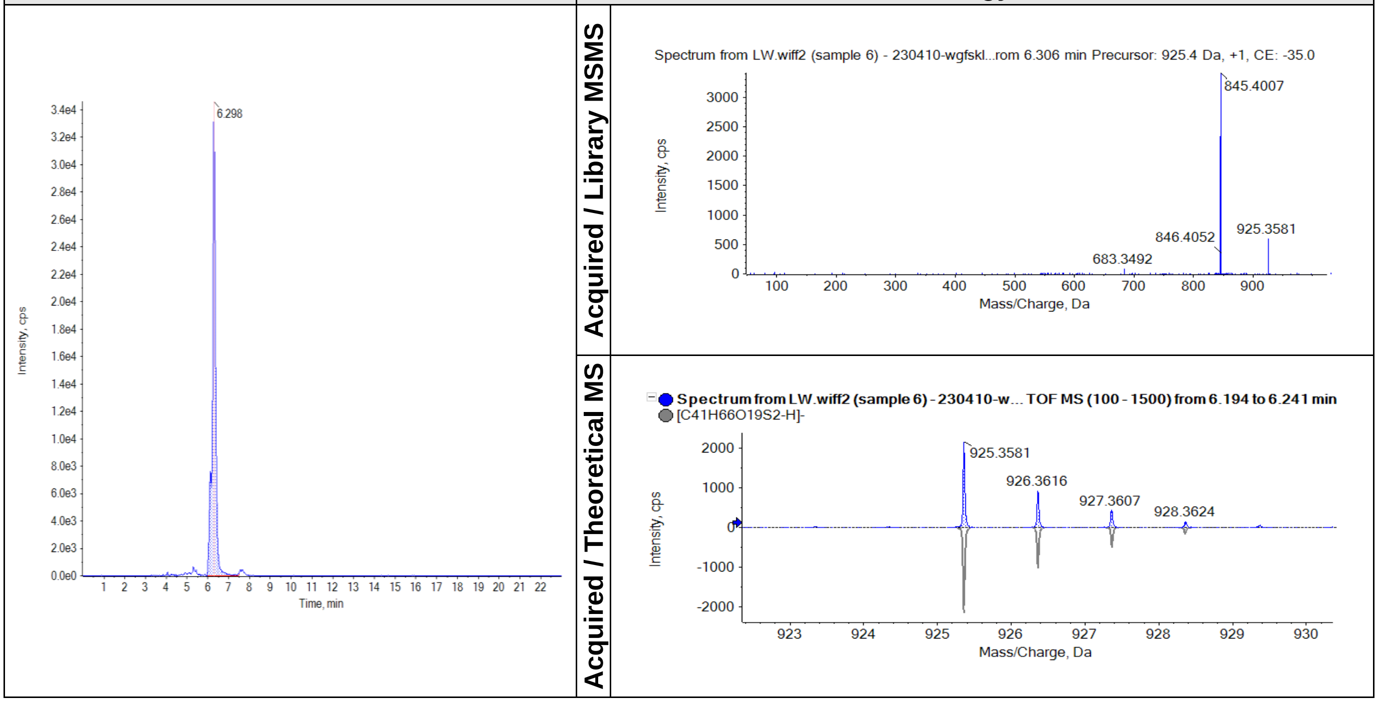

Fig. S42 Detail MS, MS/MS information of compound 46

Retention Time: 6.30 minutes
Precursor m/z : 230.0812

Exp RT: 6.31 minutes
Analyte Name:
LMZ56

Collision Energy = 35 ± 15 eV

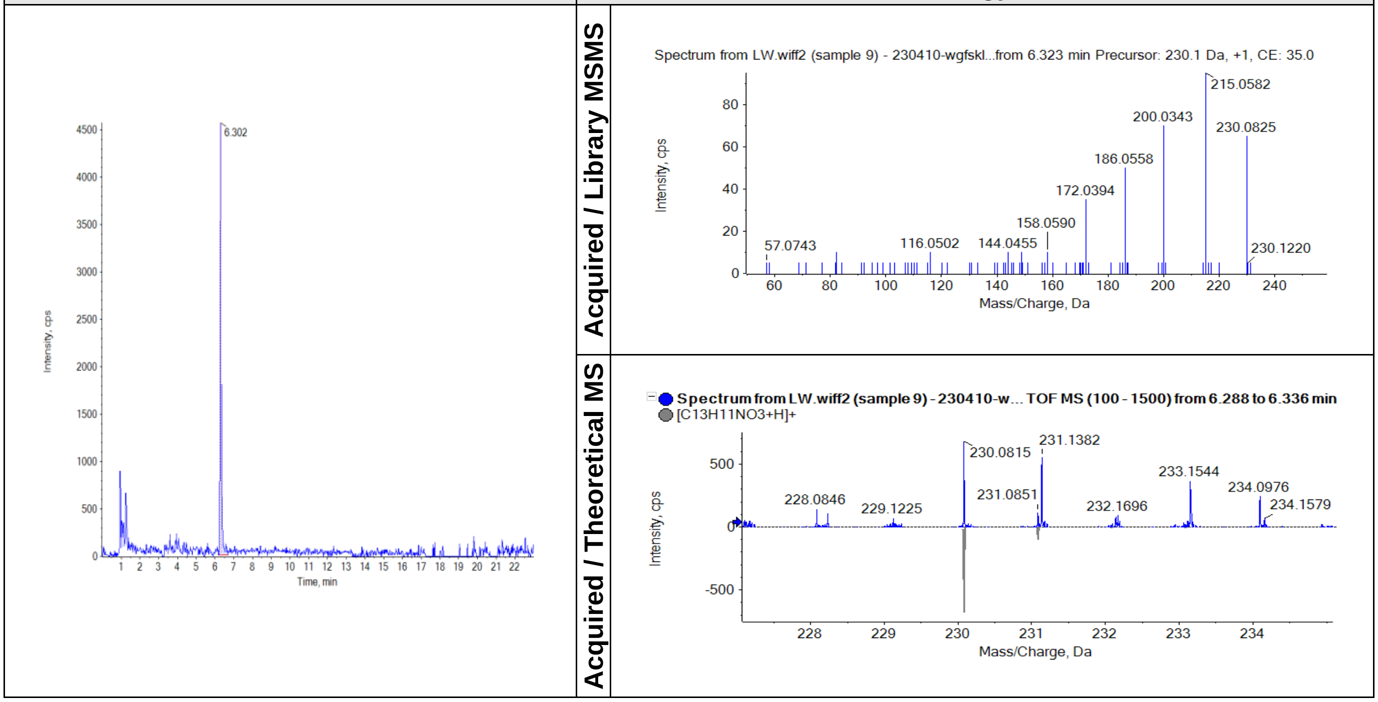

Fig. S43. Detail MS, MS/MS information of compound 47

Retention Time: 6.63 minutes
Precursor m/z : 663.3750

Exp RT: 6.64 minutes
Analyte Name:
GM174

Collision Energy = 35 ± 15 eV

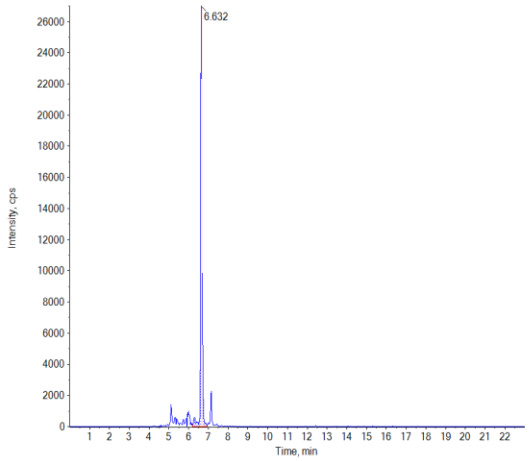

Acquired / Library MSMS

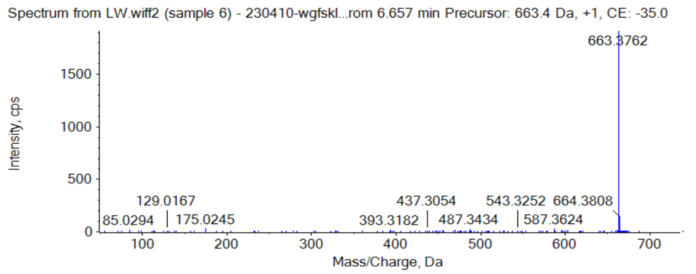

Acquired / Theoretical MS

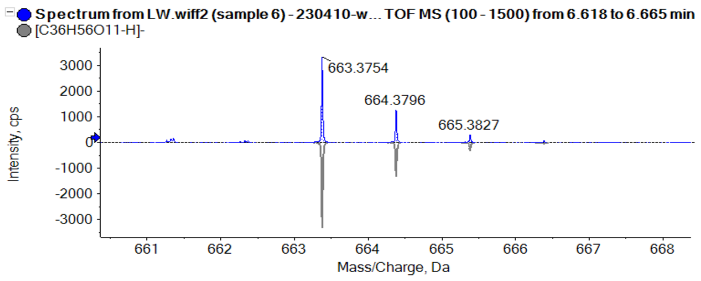

Fig. S44 Detail MS, MS/MS information of compound 48

Retention Time: 6.66 minutes
Precursor m/z : 793.4380

Exp RT: 6.67 minutes
Analyte Name:
GM178

Collision Energy = 35 ± 15 eV

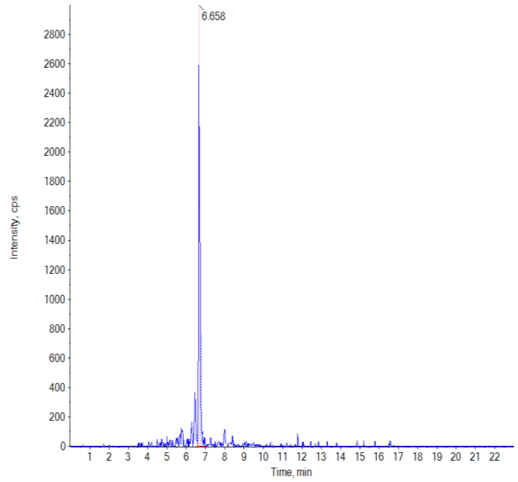

Acquired / Library MSMS

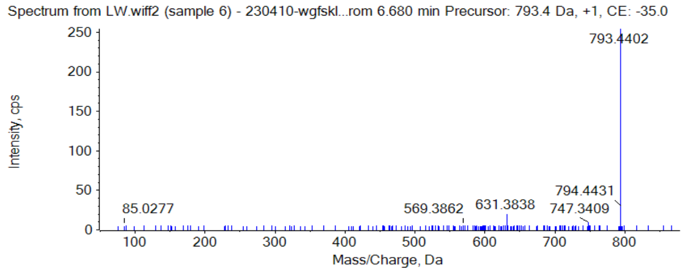

Acquired / Theoretical MS

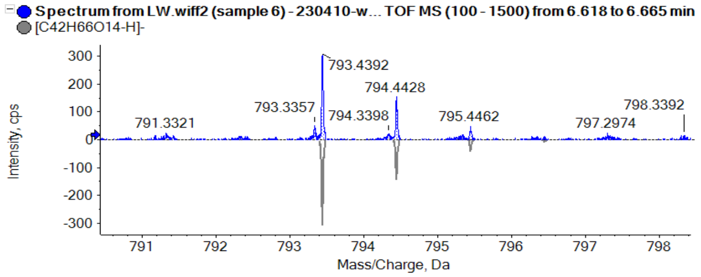

Fig. S45 Detail MS, MS/MS information of compound 49

Retention Time: 7.00 minutes
Precursor m/z : 871.3791

Exp RT: 7.05 minutes
Analyte Name: GM154

Collision Energy = 35 ± 15 eV

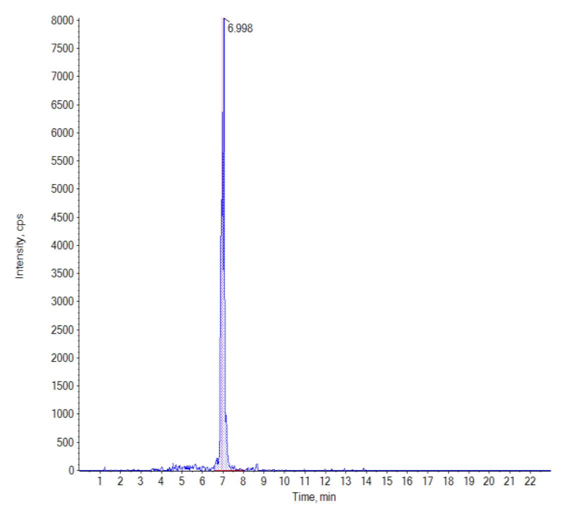

Acquired / Library MSMS

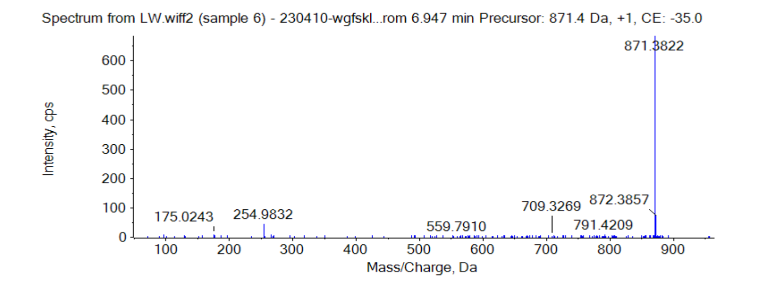

Acquired / Theoretical MS

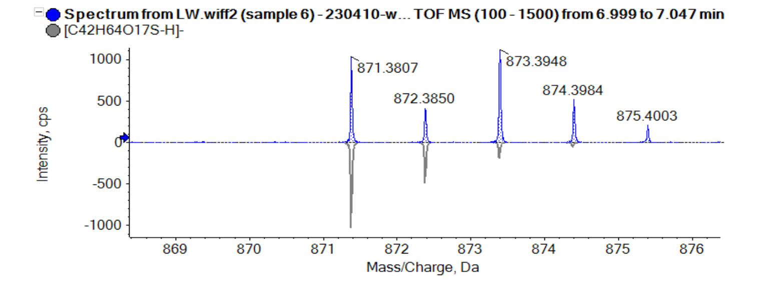

Fig. S46 Detail MS, MS/MS information of compound 50

Retention Time: 7.15 minutes
Precursor m/z : 261.1132

Exp RT: 7.14 minutes
Analyte Name: LMZ148

Collision Energy = 35 ± 15 eV

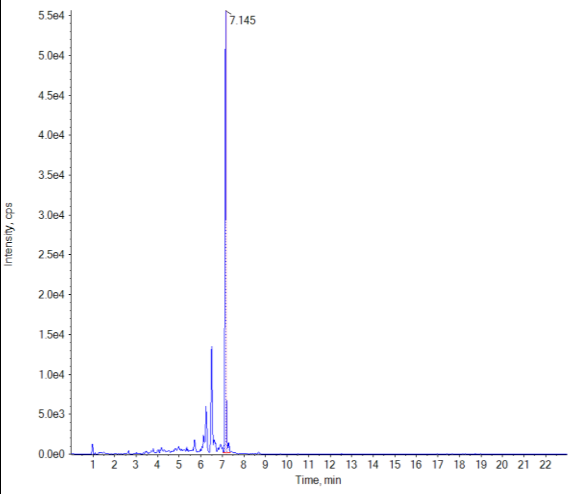

Acquired / Library MSMS

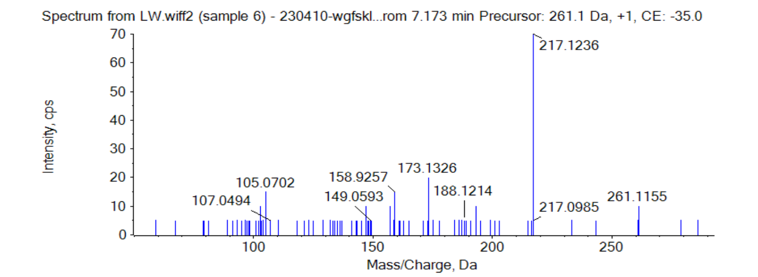

Acquired / Theoretical MS

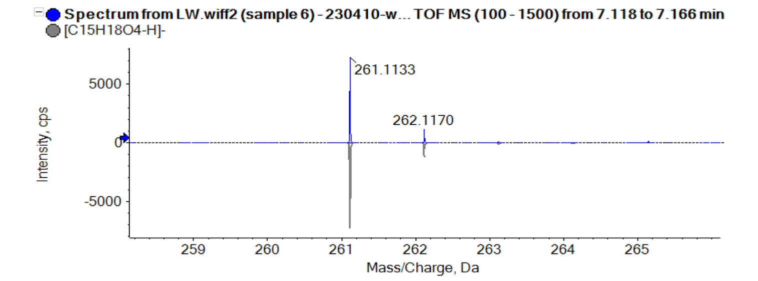

Fig. S47 Detail MS, MS/MS information of compound 51

Retention Time: 7.23 minutes
Precursor m/z : 873.3948

Exp RT: 7.24 minutes
Analyte Name: GM38

Collision Energy = 35 ± 15 eV

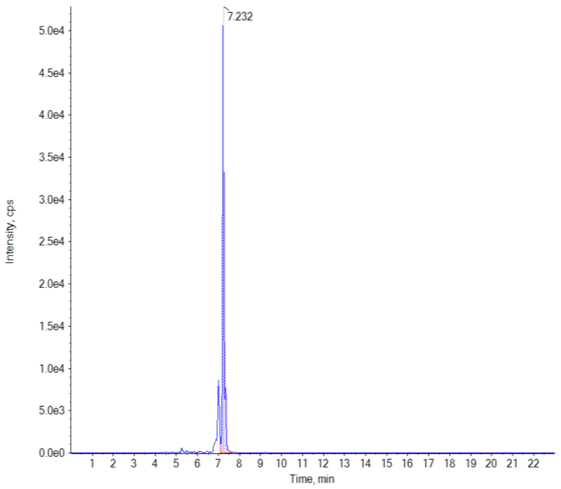

Acquired / Library MSMS

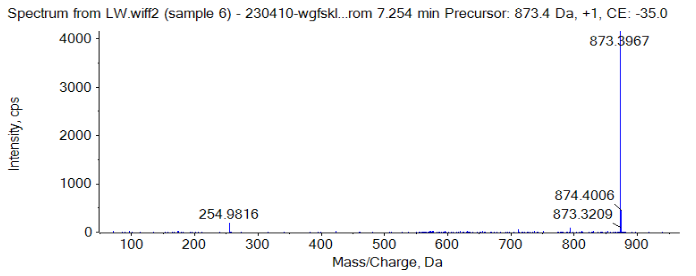

Acquired / Theoretical MS

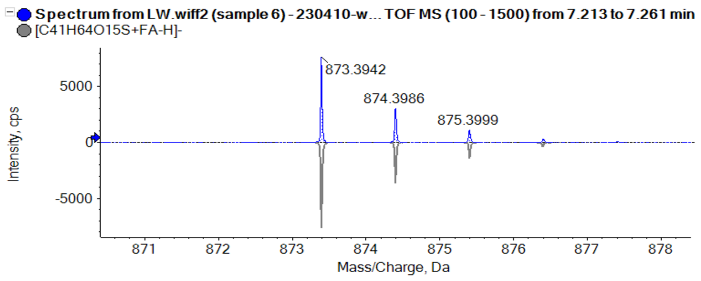

Fig. S48 Detail MS, MS/MS information of compound 52.

Retention Time: 7.25 minutes
Precursor m/z : 507.2388

Exp RT: 7.26 minutes
Analyte Name: CEC45

Collision Energy = 35 ± 15 eV

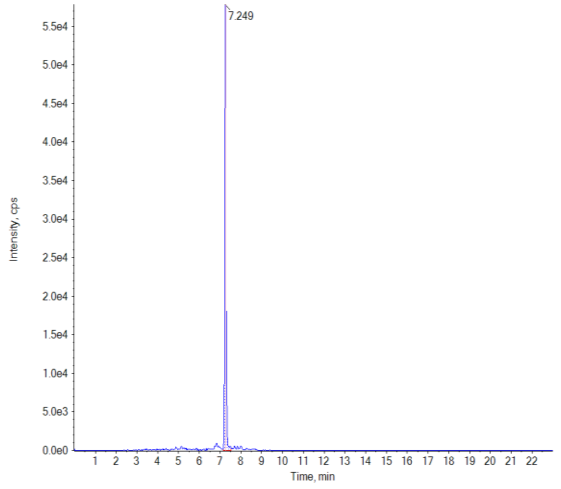

Acquired / Library MSMS

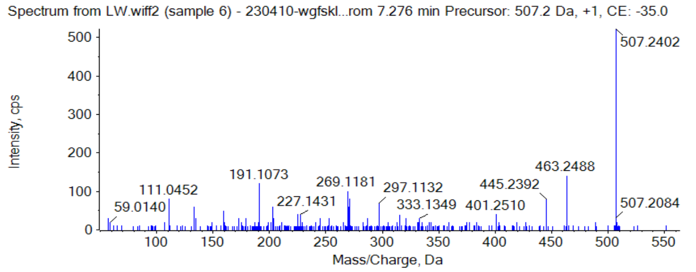

Acquired / Theoretical MS

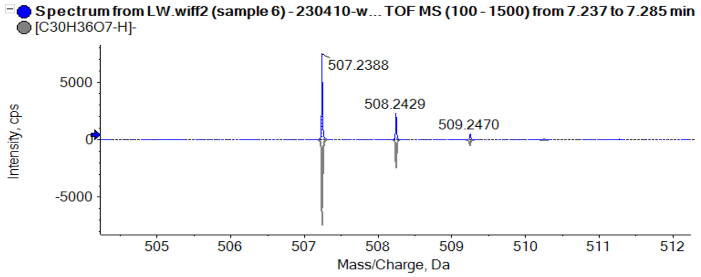

Fig. S49 Detail MS, MS/MS information of compound 53

Retention Time: 8.77 minutes
Precursor m/z : 727.3369

Exp RT: 8.78 minutes
Analyte Name: GM177

Collision Energy = 35 ± 15 eV

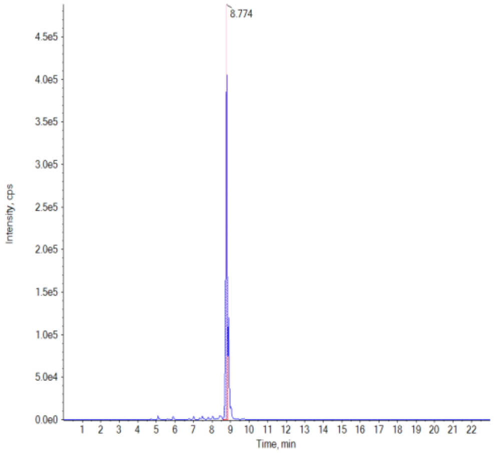

Acquired / Library MSMS

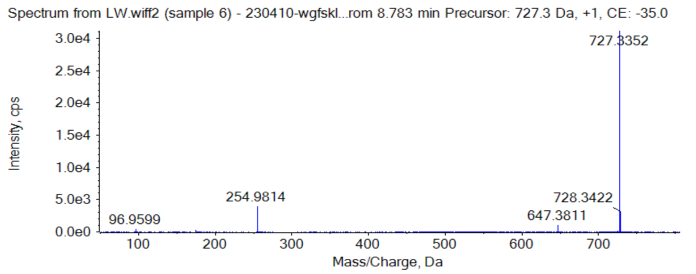

Acquired / Theoretical MS

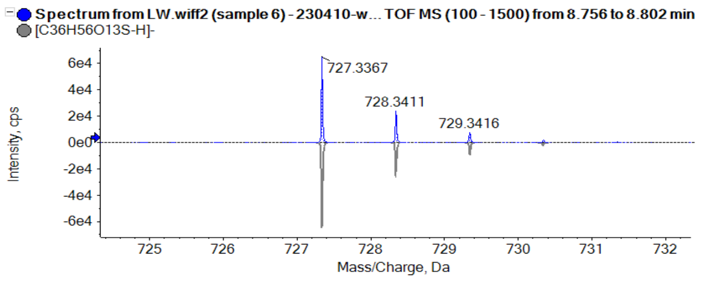

Fig. S50 Detail MS, MS/MS information of compound 54

Retention Time: 14.03 minutes
Precursor m/z : 253.2173

Exp RT: 14.02 minutes
Analyte Name: CEC54

Collision Energy = 35 ± 15 eV

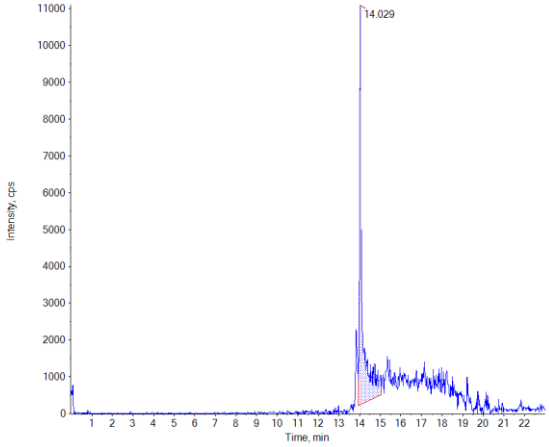

Acquired / Library MSMS

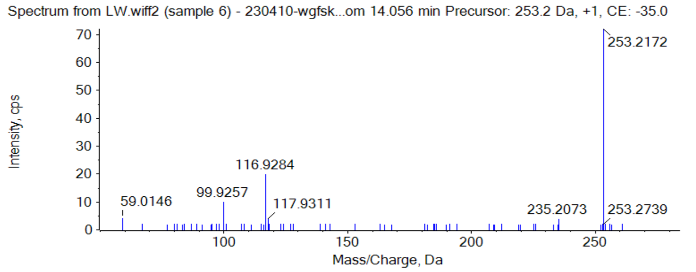

Acquired / Theoretical MS

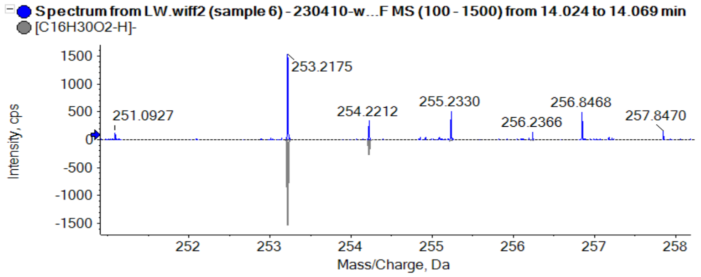

Fig. S51 Detail MS, MS/MS information of compound 55

Retention Time: 15.32 minutes
Precursor m/z : 255.2330

Exp RT: 15.33 minutes
Analyte Name: LMZ138

Collision Energy = 35 ± 15 eV

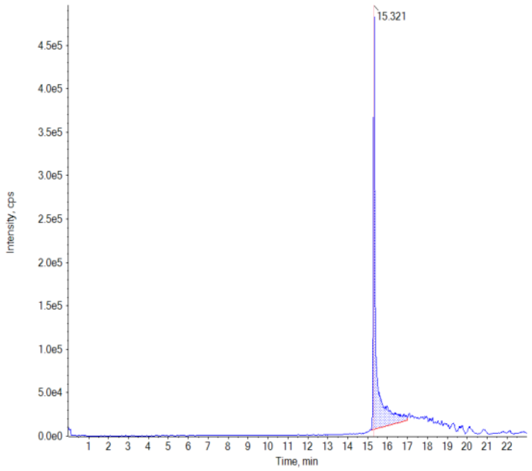

Acquired / Library MSMS

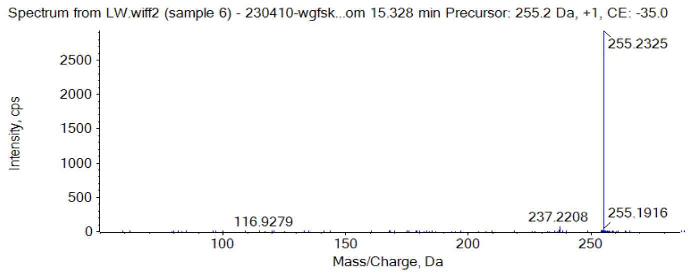

Acquired / Theoretical MS

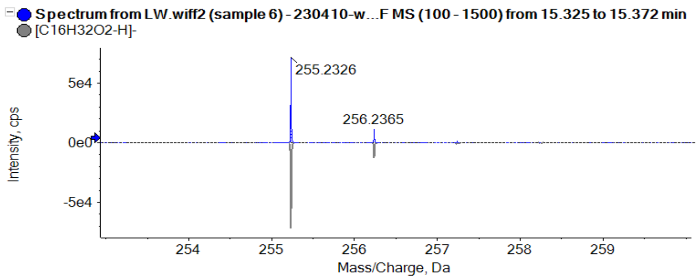

Fig. S52 Detail MS, MS/MS information of compound 56

Retention Time: 17.15 minutes
Precursor m/z : 283.2643

Exp RT: 17.14 minutes
Analyte Name: CEC56

Collision Energy = 35 ± 15 eV

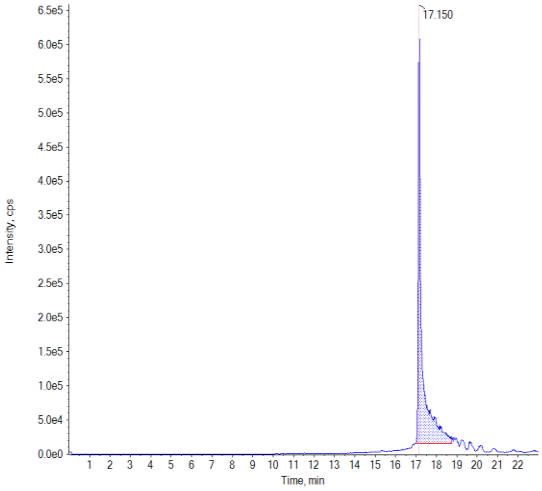

Acquired / Library MSMS

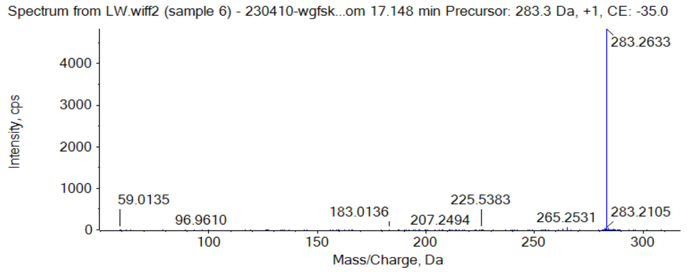

Acquired / Theoretical MS

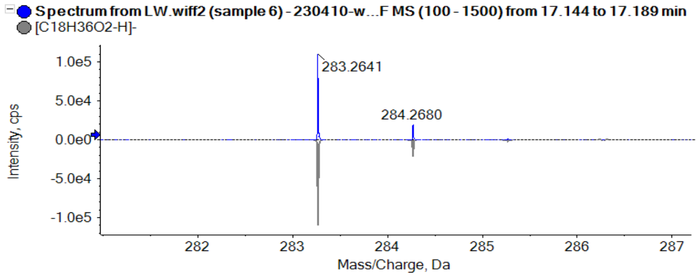

Fig. S53 Detail MS, MS/MS information of compound 57
